# Genome- and Exome-Wide Association Studies Revealed Candidate Genes Associated with DaTscan Imaging Features

**DOI:** 10.1155/2023/2893662

**Published:** 2023-08-23

**Authors:** Arash Yaghoobi, Homa Seyedmirzaei, Moein Ala

**Affiliations:** ^1^Institute for Research in Fundamental Sciences (IPM), School of Biological Sciences, Tehran, Iran; ^2^Interdisciplinary Neuroscience Research Program (INRP), Tehran University of Medical Sciences, Tehran, Iran; ^3^Universal Scientific Education and Research Network (USERN), Tehran, Iran; ^4^Experimental Research Center, School of Medicine, Tehran University of Medical Sciences, Tehran, Iran

## Abstract

**Introduction:**

Despite remarkable progress in identifying Parkinson's disease (PD) genetic risk loci, the genetic basis of PD remains largely unknown. With the help of the endophenotype approach and using data from dopamine transporter single-photon emission computerized tomography (DaTscan), we identified potentially involved genes in PD.

**Method:**

We conducted an imaging genetic study by performing exome-wide association study (EWAS) and genome-wide association study (GWAS) on the specific binding ratio (SBR) of six DaTscan anatomical areas between 489 and 559 subjects of Parkinson's progression markers initiative (PPMI) cohort and 83,623 and 36,845 single-nucleotide polymorphisms (SNPs)/insertion-deletion mutations (INDELs). We also investigated the association of cerebrospinal fluid (CSF) protein concentration of our significant genes with PD progression using PPMI CSF proteome data.

**Results:**

Among 83,623 SNPs/INDELs in EWAS, one SNP (rs201465075) on 1 q32.1 locus was significantly (*P* value = 4.03 × 10^−7^) associated with left caudate DaTscan SBR, and 33 SNPs were suggestive. Among 36,845 SNPs in GWAS, one SNP (rs12450112) on 17 p.12 locus was significantly (*P* value = 1.34 × 10^−6^) associated with right anterior putamen DaTscan SBR, and 39 SNPs were suggestive among which 8 SNPs were intergenic. We found that rs201465075 and rs12450112 are most likely related to *IGFN1* and *MAP2K4* genes. The protein level of *MAP2K4* in the CSF was significantly associated with PD progression in the PPMI cohort; however, proteomic data were not available for the *IGFN1* gene.

**Conclusion:**

We have shown that particular variants of *IGFN1* and *MAP2K4* genes may be associated with PD. Since DaTscan imaging could be positive in other Parkinsonian syndromes, caution should be taken when interpreting our results. Future experimental studies are also needed to verify these findings.

## 1. Introduction

PD is a neurodegenerative disease affecting 8–18 subjects out of 100,000 individuals annually [1]. It is characterized by tremor, dyskinesia, and rigidity [2]. The diagnosis of PD is based on clinical findings, and patients usually experience a prodromal phase before the definite diagnosis [3]. The prodromal phase mainly manifests with nonmotor symptoms such as constipation, hyposmia, REM-sleep behavior disorder, depression, anxiety, and cognitive impairment [3]. The pathological hallmark of PD is the aggregation of misfolded *α*-synuclein, also known as Lewy body, resulting in the loss of dopaminergic neurons in the substantia nigra [4]. The diagnosis of PD is not usually made until at least 30% of dopaminergic neurons and 50–60% of their axon terminals in substantia nigra are lost [5].

The exact etiology of PD is not yet fully understood; however, a higher incidence of PD in monozygotic compared to dizygotic twins and in people with a family history of PD than in those without a family history of PD highlights the prominent role of genetics in PD [6, 7]. Genetic studies have revealed several monogenic causes of PD, such as *SNCA*, *PRKN*, *LRRK2*, *PINK1*, *DJ-1*, *VPS35*, and *GBA* [8]. Recently, GWAS and EWAS have indicated the involvement of numerous genetic polymorphisms in PD [9]. Despite all these findings, the overall estimated heritability of PD is about 60%, and the known genetic variants do not account for all of it [10, 11].

An alternative approach to better identify the susceptibility genes for complex traits is the endophenotype approach [12]. An endophenotype refers to a biological or psychological feature of a disease believed to be in the causal chain between genetic backgrounds and diagnosable symptoms of the disease [13]. Neuroimaging endophenotypes have been widely used in neurological disorders such as PD. Neuroimaging endophenotypes provide quantitative measures of the brain structure or function that index genetic liability for a neurological condition [14]. In the substantia nigra of patients with PD, these endophenotypes can appear as reduced fractional anisotropy in diffusion tensor imaging, increased echogenicity in transcranial sonography, and decreased signal in the dorsolateral segment in magnetic resonance imaging (MRI) [15]. However, more specific and sensitive neuroimaging tools are needed to assess brain changes in PD.

Dopamine transporter single-photon emission computerized tomography (DaTscan) is a relatively new imaging modality in PD. DaTscan utilizes ioflupane I-123 injection to visualize the striatal dopamine transporters [16]. Ioflupane I-123 is derived from cocaine compounds and binds to dopamine active transporter (DAT), thereby providing a specific binding ratio (SBR) [17]. Due to its high sensitivity (84.4%) and specificity (96.2%), DaTscan has been used to diagnose PD and differentiate it from other neurodegenerative disorders in probable cases [18]. It also plays a role in detecting the preclinical phase of PD, as a longitudinal study has shown that SBR decline begins before the manifestation of motor symptoms and continues to decrease during disease progression [19].

Given the information above, we hypothesized that the genetic association study combined with neuroimaging findings from DaTscan as endophenotypes can reveal genetic basis of PD more accurately compared to case-control studies. Moreover, we aimed to conduct our genomic analyses on a mixed population (PD cases, prodromal patients, and healthy controls) because we believed that increasing the phenotypic variance increases the power of our study. Given the information above, we hypothesized that the underlying genetic basis of PD may be associated with neuroimaging findings from DaTscan. Compared with noncoding variants, exomes' variants can better prioritize the putative causal genes in PD [20]. As a result, we aimed to explore the possible associations between striatal DaTscan findings and SNPs by conducting EWAS and GWAS on data from Parkinson's progression markers initiative (PPMI) cohort.

## 2. Method

### 2.1. Study Design

We used data from the PPMI cohort in our study. In brief, PPMI is a longitudinal, observational, and multicenter study. The primary goal of the PPMI cohort was to assess clinical and neuroimaging features and genetic and biological markers across all stages of PD. The PPMI cohort began in 2010, and after an enrollment gap, the cohort reinitiated recruitment in 2020. The study included patients with PD, patients in the prodromal stage of PD, and healthy controls. All samples were collected using the relevant guidelines. All subjects signed the consent form, and the Regional Ethics Committees approved this cohort. The details of this cohort and its protocol can be found on its website (https://www.ppmi-info.org/) or through Supplementary Material 1. By filling out the data access request form and specifying our research purpose, we were allowed to access the database of this cohort in the LONI database (https://ida.loni.usc.edu/).

### 2.2. Participants

We used the ppmi_wes_645_cohort_vcf.tar, PPMI_NEUROX_Nov11th2013.zip, DaTScan_Analysis.csv, Age_at_visit.csv, and Demographics.csv files, which contained whole-exome genetic polymorphism data of 654 subjects, whole-genome SNP data of 619 subjects, six neuroimaging features from DaTscan in 2930 visits of participants, and participants' age and demographic information in their screening visits. We chose screening visits of PPMI cohort patients for our study; because based on inclusion and exclusion criteria of PPMI cohort, the DaTscan values of patients were least confounded by disease progression or anti-Parkinson's drugs.

### 2.3. Quality Control

We performed different stages of quality control on whole-exome sequence data before performing EWAS. In summary, exome sequencing was performed on whole blood-derived DNA samples following the PPMI Research Biomarkers Laboratory Manual using Illumina Nextera Rapid Capture Expanded Exome kit in 2015. Nextera Expanded Exome targets 201,121 exons, UTRs, and miRNA and covers 95.3% of Refseq exome. More details about the exome data in the vcf file can be found on the cohort website. There were data about 707,050 genetic polymorphisms, including SNPs and INDELs of 645 subjects. We first converted the .vcf file to the PLINK binary files with bed suffix using PLINK 1.9 software (https://www.coggenomics.org/plink/). Quality control of the exomic data was performed using PLINK 1.9 software. All SNPs of the sex chromosomes were excluded. SNPs with minor allele frequency (MAF) of less than 5%, missing genotype rate of less than 95%, and Hardy–Weinberg equilibrium (HWE) *P* value of less than 0.000001 were excluded. Participants with a missing genotype rate of less than 95% and a heterozygosity rate of more than three standard deviations from the mean value were excluded. In the next step, the exomic data satisfying the quality control were merged with the neuroimaging features in screening visits and demographic characteristics.

We used the NeuroX SNP data of the original cohort to perform GWAS. In summary, SNP genotyping was performed using the Illumina NeuroX array. The NeuroX array is an Illumina Infinium iSelect HD custom genotyping array containing 267,607 Illumina standard content exomic variants and 24,706 custom variants designed for studying neurological diseases. There were data of 267,607 SNPs from 619 subjects. All quality control steps were performed similarly to the exomic data. Finally, the genomic data satisfying the quality control were merged with data from DaTscan.

### 2.4. DaTscan

DaTscan was performed in PPMI imaging centers. The imaging protocol of PPMI is available at http://www.ppmi-info.org/study-design/research-documents-and-sops/. Before injecting ioflupane I-123, females with possible pregnancy underwent a urine pregnancy test. Spatial normalization for consistent orientation on DaTscan, reconstruction, and attenuation correction was conducted according to PPMI protocol. Count densities were extracted from striatal regions of interest, including the caudate nucleus and putamen, and the occipital cortex was considered the reference region. The regional SBR was calculated using the following formula: (SBR) = (striatal region)/(occipital) − 1 [21]. In our study, the SBR scores of subjects at the screening visit were used for the analysis.

### 2.5. EWAS and GWAS

We used GCTA 1.94.1 software (https://yanglab.westlake.edu.cn/software/gcta/#Download) to perform EWAS and GWAS. We used a mixed linear model with the --mlma command. Mixed statistical models were used and corrected for the following confounding factors in genome-wide association analyses: genetic relatedness and population structure (ethnicity). The details of the GCTA software and mixed model association methods have been described elsewhere [22, 23]. The mathematical equation of this statistical model is as follows.

*y* = *a* + *bx* + *g* + *e*, where *y* is the phenotype (DaTscan features in our study), *a* is the mean term, *b* is the additive effect (fixed effect) of the candidate SNP to be tested for association, and *x* is the SNP genotype indicator variable coded as 0, 1, or 2. *g* is the polygenic effect (random effect), i.e., the accumulated effect of all SNPs (as captured by the genetic relatedness matrix (GRM) calculated using all SNPs), and *e* is the residual. We used age and gender as covariates. Using the --mlma-no-preadj-covar option, covariates were fitted together with the SNP for the association test. For ease of computation, the genetic variance, var(g), was estimated based on the null model, i.e., *y* = *a* + *g* + *e*, and then fixed while measuring for the association between each SNP and the trait. Using the Bonferroni correction method, the suggestive and significant thresholds for *Pvalue*s were calculated based on the number of SNPs/INDELs used in our study. After performing EWAS and GWAS on six neuroimaging endophenotypes, we computed the *λ*-statistic to evaluate the degree of genomic inflation for adjusting population stratification. Thereafter, we highlighted the observed versus expected *Pvalues* in the Q-Q plot by the qqman *R* package in *R* software version 4.2.2. We also used the qqman *R* package for generating the GWAS Manhattan plot.

### 2.6. Variant Annotation and Functional Fine Mapping

The positional gene mapping for all suggestive and significant SNPs/INDELs was performed using the UCSC genome browser website (https://genome.ucsc.edu/) based on the version of GRCh37/hg19. Using the Genotype-Tissue Expression (GTEx) version 8 (V8) data GTEx website (https://gtexportal.org/home/index.html), top genes with a significant eQTL *P* value related to the SNPs/INDELs in 3 tissues (caudate nucleus, putamen nucleus, and substantia nigra) were identified for functional annotation. Finally, information about the association of identified genes with PD or PD-related phenotypes was evaluated on the GWAS catalog website (https://www.ebi.ac.uk/gwas/) and the Genecards website (https://www.genecards.org/) to identify novel candidate genes potentially associated with PD.

### 2.7. Replication of Significant Genes with CSF Proteome Data

In order to confirm the potential role of statistically significant genes identified in EWAS and GWAS, we investigated whether the CSF concentration of proteins produced by these genes is associated with PD progression. We used ordinal regression with PD status as the response variable (control = 0, prodromal = 1, and PD = 2) and age and gender as covariates. *P* values less than 0.05 were considered statistically significant.

### 2.8. CSF Proteome Data

Proteomics data from the CSF of patients with PD and healthy volunteers were measured using the SOMAscan platform. The data were quality controlled by removing outlier samples, calibrators, buffer, and nonhuman SOMAmers. The measured values were hybridization normalized, plate scaled, median normalized intraplate, and calibrated at SomaLogic's side, then log 2 transformed, median normalized interplates, and batch corrected at the plate level. More details about the quality control steps of proteomic data can be found on the PPMI website.

### 2.9. Results

In the exomic data, 557,019 SNPs/INDELs with MAF of less than 5%, 60,100 SNPs/INDELs with a missing genotype rate of less than 95%, 6,212 SNPs/INDELs with Hardy–Weinberg *Pvalue* of less than 0.000001, and 96 SNPs/INDELs on sex chromosomes were excluded. In addition, 95 subjects with less than 95% missing genotype rates and 13 participants with a heterozygosity rate of more than three standard deviations from the mean were excluded. Finally, the genetic data of 537 participants and 83,623 exomic SNPs/INDELs passed all steps of quality control. Among 537 participants, data from DaTscan and demographic characteristics were available for 489 participants. Among the 489 participants, there were 124 healthy subjects, 317 patients with PD, and 48 subjects from the SWEDD cohort (patients with symptoms of PD and normal DaTscan). The mean age of participants was 61.23 ± 10.08 years. Finally, EWAS for six neuroimaging endophenotypes was performed on 489 subjects (318 males and 171 females). Based on the Bonferroni correction method, the statistically significant and suggestive threshold in our EWAS were *P* value <5.97 × 10^−7^ and 1.19 × 10^−4^ <*P* value <5.97 × 10^−7^, respectively. The *λ*-statistics indicating the degree of genomic inflation was low in our 12 analyses. Manhattan plots and Q-Q plots of six endophenotypes are shown in Supplementary Material 2.

In the NeuroX genotyping data, 217,443 SNPs with MAF of less than 5%, 12,461 SNPs with a missing genotype rate of less than 95%, 191 SNPs with Hardy–Weinberg *Pvalue* of less than 0.000001, and 667 SNPs on sex chromosomes were excluded. In addition, 13 subjects with less than 95% missing genotype rates and 2 participants with a heterozygosity rate of more than three standard deviations from the mean were excluded. Finally, the genetic data of 606 participants and 36,845 SNPs passed all steps of quality control. Among 606 participants, data from DaTscan and demographic characteristics were available for 559 participants. Among 559 participants, there were 148 healthy subjects, 359 patients with PD, and 52 subjects from the SWEDD cohort (patients with symptoms of PD and normal DaTscan). The mean age of participants was 61.06 ± 10.34 years. Finally, GWAS for six neuroimaging endophenotypes was performed on 559 subjects (365 males and 194 females). Based on the Bonferroni correction method, the statistically significant and suggestive threshold in our GWAS were *P* value <1.35 × 10^−6^ and 2.71 × 10^−4^ <*P* value <1.35 × 10^−6^, respectively. The *λ*-statistic indicating the degree of genomic inflation was low in our six analyses. Manhattan plots and Q-Q plots of six endophenotypes are shown in Supplementary Material 3.

Among 83,623 SNPs/INDELs in EWAS, one SNP (rs201465075) reached the statistically significant threshold (*P* value = 4.03 × 10^−7^) associated with left caudate DaTscan SBR and 33 SNPs were considered suggestive. Positional gene mapping identified 30 candidate genes associated with the DaTscan features. The results of all SNPS with their mapped genes are shown in Table 1.

Among 36,845 SNPs in GWAS, one SNP (rs12450112) reached the statistically significant threshold (*P* value = 1.34 × 10^−6^) associated with right anterior putamen DaTscan SBR, and 39 SNPs were suggestive among which 8 SNPs were intergenic. To perform positional gene mapping for intergenic SNPs, two nearest genes on the left and right sides of the SNP were reported. The complete results of GWAS are shown in Table 2.

Among all suggestive and significant SNPs/INDELs in EWAS and GWAS, 16 SNPs were eQTL for 12 genes in three tissues relevant to PD pathology (caudate nucleus, putamen nucleus, and substantia nigra): *ANKRD65*, *B3GALT6*, *OBSCN*, *WNT3A*, *CCDC25*, *ZNF471*, *AC007228*.*11*, *IDUA*, *KRI1*, *UBE3D*, *GAS2L1P2*, and *VPS28*. The complete data of mentioned genes with their eQTLs are shown in Table 3.

### 2.10. CSF Proteome Analysis

One of our 2 significant SNPs was mapped to the *IGFN1* gene. The other significant SNP was near the *MAP2K4* gene. We hypothesized that the concentration of protein products of these 2 genes in CSF may be associated with PD progression. Only the CSF concentration of MAP2K4 protein was available in the PPMI CSF proteome dataset. The CSF concentration of MAP2K4 and demographic information were extracted for 1,156 subjects. The mean age of subjects was 61.64 ± 9.32 years. There were 637 males and 517 females. There were 185 healthy subjects, 354 patients in the prodromal phase of PD, and 617 patients with PD in our analysis. The mean CSF concentration of MAP2K4 among healthy subjects, patients in the prodromal phase of PD, and patients with PD were 7.82 ± 0.49, 7.79 ± 0.53, and 7.95 ± 0.59, respectively. The mean CSF concentration of MAP2K4 among all subjects was 7.88 ± 0.56. There was a statistically significant association between the concentration of MAP2K4 in CSF and PD progression across the PD spectrum (*P* value = 0.001).

## 3. Discussion

Using data from the PPMI cohort, we performed EWAS and GWAS and identified two loci, 1q32.1 and 17p12, at *IGFN1* gene and near *MAP2K4* gene, with a significant association with striatum DaTscan SBR which may have implications in PD pathology. We also found several suggestive genes (Table 4) associated with DaTscan SBR. Furthermore, the CSF proteome analysis showed that increased CSF concentration of MAP2K4 is associated with PD progression.

Based on The Human Protein Atlas, immunoglobulin-like and fibronectin type III domain containing 1 (*IGFN1*) is highly expressed in the muscular tissue and has low expression in the central nervous system (CNS) and basal ganglia [24]. *IGFN1* is essential for myoblast fusion and differentiation [25]. *IGFN1* is upregulated during muscle denervation and interacts with eukaryotic translation elongation factor 1A (eEF1A), thereby downregulating protein synthesis during muscle denervation [26]. eEF1A2 knockdown has been associated with impaired autophagy, mitochondrial dysfunction, *α*-synuclein deposition, and apoptosis in the 1-methyl-4-phenylpyridinium ion (MPP^+^)-induced cellular model of PD [76]. Giri et al. indicated that loss of function in the *IGFN1* gene is significantly associated with Parkinson's disease [27]. Nikonova et al. compared differentially expressed genes between kinase hyperactive G2019S transgenic mice and mice with knockout of leucine-rich repeat kinase 2 (*LRRK2*), a common genetic cause of both autosomal dominant familial and sporadic PD. They indicated that *IGFN1* gene was one of the differentially expressed genes between *LRRK2* knockout mice and kinase hyperactive G2019S transgenic mice [77]. Lavin et al. found that rehabilitative training enhances *IGFN1* expression in the skeletal muscle of patients with PD; however, data are scarce about its status in the CNS [78]. In our analysis, rs201465075 was significantly associated with left caudate SBR. The rs201465075 is a missense variant in *IGFN1* gene. It may alter the *IGFN1* protein structure. There are no data about the effect of this SNP on gene expression of nearby genes.

The *MAP2K4* gene, also known as *MKK4*, encodes mitogen-activated protein kinase 4, a member of the mitogen-activated protein kinase (*MAPK*) family [79]. This family of proteins is an integration point for intracellular signaling pathways and has been implicated in various cellular processes such as proliferation, differentiation, and transcription regulation [80]. The *MAP2K4* gene has been previously implicated in the pathogenesis of Alzheimer's disease [81]. Chen et al. showed that the G2019S mutation of LRRK2 enhanced its kinase activity and led to overphosphorylation of MAP2K4 [82]. LRRK2-mediated overphosphorylation of MAP2K4 upregulated and activated proapoptotic factors such as Fas ligand, caspase-9, caspase-8, and caspase-3 in the dopaminergic neurons of substantia nigra in transgenic mice, resulting in apoptotic neuronal death [82]. However, there were not any subjects with LRRK2 mutation in GWAS and EWAS samples.

It was also observed that MAP2K4 activated c-Jun N-terminal kinase (JNK)-mediated apoptosis in the MPP^+^-induced cellular model of PD [83]. Consistently, inhibition of JNK attenuated MAP2K4-mediated neuronal death [83, 84]. Interestingly, Shakespear et al. found that miR-200a-3p targets 3′-UTR of *MAP2K4* gene/MKK4 mRNA and downregulates MAP2K4 mRNA and protein expressions, thereby preventing apoptosis of MPP^+^-treated SH-SY5Y cells [85]. In our study, rs12450112 near to *MAP2K4* gene was significantly associated with right anterior putamen SBR. Due to lack of data about the effect of this SNP on gene expression, we hypothesized that MAP2K4 is the potential causal gene involved in PD pathology. Interestingly, CSF proteome analysis revealed that CSF concentration of MAP2K4 increases across the PD spectrum. So, *MAP2K4* gene may be a potential causal gene in PD pathology. However, there are other genes such as myocardin (MYOCD), zinc finger protein 18 (ZNF18), dynein axonemal heavy chain 9 (DNAH9), and Rho GTPase activating protein 44 (ARHGAP44) near our significant SNP. Our significant SNP may also affect the expression level of these genes. Thus, further studies such as single cell-based integration of omic data or CRISPR-based experimental methods are needed to link our significant SNP to the target genes.

Our study has several limitations: There are no data regarding the effect of our two significant SNPs on gene expression or protein structure of related genes. Unfortunately, there is not any compelling evidence in various bioinformatics databases about the effect of our two significant SNPs on the expression level of nearby genes. No eQTLs were associated with our two significant SNPs in various external studies. Also, data on the protein level of *IGFN1* do not exist in the PPMI cohort database. Also, DaTscan can be positive in various types of Parkinsonian syndromes. Therefore, the significant genes identified in our analyses can be associated with other forms of Parkinsonian syndromes rather than PD. Although using mixed population in analyses increased the power of our study for detecting significant SNPs, our approach has important limitations due to our nonhomogenous samples regarding various disease statuses of subjects.

## 4. Conclusion

Analyzing data from the PPMI cohort, EWAS and GWAS identified two potential genes, *IGFN1* and *MAP2K4*, with a significant association with DaTscan SBR and PD. Furthermore, we found several suggestive genes (Table 4) for PD by EWAS and GWAS. Moreover, the CSF proteome data showed that the CSF concentration of MAP2K4 is associated with PD progression. More sample sizes should be used for future studies. The exact role of our significant SNPs and genes should be investigated in experimental and animal studies.

## Figures and Tables

**Table 1:** DaTscan EWAS summary statistics.

Chr	SNP	Effect allele	Effect allele frequency	Effect size	SE	Mapped gene	DaTscan anatomical feature	*P* value
1	rs10864219	A	0.49	0.19	0.04	USH2A	Right caudate	2.18 × 10^−5^

1	rs78555129	G	0.10	0.29	0.07	C1QTNF12	Right caudate	0.00010

1	rs199737720	G	0.06	0.46	0.10	*IGFN1*	Left caudate	8.63 × 10^−6^
				0.42	0.10		Left anterior putamen	8.89 × 10^−5^

1	rs201465075	G	0.08	0.46	0.09	*IGFN1*	Left caudate	4.03 × 10^−7^
				0.35	0.09		Right putamen	8.54 × 10^−5^
				0.41	0.09		Left putamen	8.83 × 10^−6^
				0.37	0.09		Right anterior putamen	6.48 × 10^−5^
				0.43	0.09		Left anterior putamen	5.12 × 10^−6^

2	rs112724571	A	0.05	−0.44	0.11	TMEM169	Left caudate	9.57 × 10^−5^

3	rs41271391	T	0.12	−0.29	0.07	CD80	Left caudate	5.60 × 10^−5^

3	rs16829980	G	0.12	−0.29	0.07	CD80	Left caudate	5.60 × 10^−5^

3	rs2305035	A	0.22	0.22	0.05	CBLB	Left putamen	0.000112

4	rs17612630	A	0.20	0.25	0.06	DDX60L	Right putamen	2.93 × 10^−5^
				0.24	0.06		Right anterior putamen	0.00010

4	rs6964	A	0.31	−0.21	0.05	GAK	Left anterior putamen	4.71 × 10^−5^

5	rs6873053	A	0.08	0.35	0.09	TIMD4	Right putamen	9.33 × 10^−5^
				0.36	0.09		Left putamen	0.00010

6	rs41266301	C	0.10	−0.30	0.07	KIF25	Left putamen	8.81 × 10^−5^

6	rs3024466	A	0.16	0.27	0.06	F13A1	Right anterior putamen	3.85 × 10^−5^
				0.27	0.06		Left anterior putamen	3.85 × 10^−5^

6	rs1050783	T	0.16	0.27	0.06	F13A1	Right anterior putamen	3.15 × 10^−5^
				0.27	0.06		Left anterior putamen	3.00 × 10^−5^

7	rs41280648	A	0.05	−0.44	0.10	OGDH	Right caudate	4.40 × 10^−5^

7	rs2906997	G	0.06	0.38	0.09	ZP3	Left caudate	0.000117

7	rs3800769	C	0.42	−0.18	0.04	SLC13A4	Left putamen	0.00010
				−0.21	0.04		Left anterior putamen	1.98 × 10^−5^

8	rs2451153	A	0.34	−0.20	0.05	SAMD12	Right caudate	8.29 × 10^−5^

8	rs2514743	C	0.35	−0.19	0.05	SAMD12	Right caudate	0.00010

8	rs2280838	C	0.44	−0.17	0.04	SLC39A4	Right putamen	0.00010

9	rs15394	C	0.15	−0.26	0.06	CTSV	Right caudate	8.07 × 10^−5^

9	rs62560873	A	0.14	−0.26	0.06	GAS2L1P2	Right caudate	9.54 × 10^−5^
				−0.28	0.06		Right putamen	2.69 × 10^−5^
				−0.27	0.07		Left putamen	7.70 × 10^−5^
				−0.28	0.07		Right anterior putamen	5.84 × 10^−5^
				−0.29	0.07		Left anterior putamen	4.30 × 10^−5^

9	rs16924211	G	0.14	−0.29	0.06	GAS2L1P2	Right caudate	9.81 × 10^−6^
				−0.29	0.06		Left caudate	1.59 × 10^−5^
				−0.29	0.06		Right putamen	8.81 × 10^−6^
				−0.28	0.06		Left putamen	3.38 × 10^−5^
				−0.30	0.06		Right anterior putamen	1.10 × 10^−5^
				−0.30	0.07		Left anterior putamen	1.40 × 10^−5^

12	rs12320366	T	0.13	0.28	0.06	KERA	Right caudate	3.61 × 10^−5^

12	rs11615432	G	0.08	0.34	0.08	GALNT8/KCNA6	Right putamen	6.80 × 10^−5^

12	rs7955650	C	0.05	0.44	0.10	TBC1D15	Left putamen	2.65 × 10^−5^
				0.42	0.10		Left anterior putamen	7.46 × 10^−5^

13	rs41288262	C	0.09	−0.32	0.08	POU4F1	Left caudate	6.73 × 10^−5^
				−0.31	0.09		Left putamen	0.00010

13	rs61947037	C	0.43	−0.20	0.05	PARP4	Right anterior putamen	6.27 × 10^−5^

18	rs13732	T	0.46	−0.18	0.04	FECH	Right caudate	0.000117

19	rs10401255	C	0.05	0.47	0.10	ZNF586	Left caudate	1.18 × 10^−5^
				0.51	0.10		Right putamen	1.01 × 10^−6^
				0.53	0.11		Left putamen	1.59 × 10^−6^
				0.51	0.10		Right anterior putamen	1.94 × 10^−6^
				0.50	0.11		Left anterior putamen	4.99 × 10^−6^

19	rs16027	T	0.09	0.33	0.08	CACNA1A	Right putamen	6.10 × 10^−5^

19	rs56283800	T	0.07	0.34	0.08	CYP2A6	Right putamen	7.64 × 10^−5^

19	rs2270683	A	0.11	0.31	0.07	DACT3	Right putamen	3.13 × 10^−5^
				0.32	0.07		Right anterior putamen	2.53 × 10^−5^

21	rs2839531	T	0.07	0.37	0.09	RSPH1	Left anterior putamen	8.11 × 10^−5^

**Table 2:** DaTscan GWAS summary statistics.

Chr	SNP	Effect allele	Effect allele frequency	Effect size	SE	Mapped gene	DaTscan anatomical feature	*P* value
1	exm-rs2749097	C	0.18	−0.22	0.05	PGM1-ROR1	Right putamen	1.97 × 10^−4^
				−0.22	0.05		Left putamen	2.06 × 10^−4^
				−0.23	0.06		Right anterior putamen	1.48 × 10^−4^

1	exm2250721_ver4	A	0.30	0.19	0.04	PROX1-SMYD2	Right putamen	7.57 × 10^−5^
	rs1147673			0.18	0.04		Right anterior putamen	2.14 × 10^−4^

1	exm1952	G	0.10	0.27	0.07	C1QTNF12	Right caudate	1.11 × 10^−4^
	rs78555129							

1	exm1971	A	0.09	0.27	0.07	C1QTNF12	Right caudate	1.87 × 10^−4^
	rs12093154							

1	exm156096 rs1188732	T	0.31	−0.18	0.04	OBSCN	Right caudate	2.33 × 10^−4^
				−0.18	0.05		Left caudate	2.23 × 10^−4^

1	exm2250840 rs12035900	T	0.31	−0.18	0.04	OBSCN	Right caudate	2.17 × 10^−4^
				−0.18	0.05		Left caudate	1.92 × 10^−4^

2	exm2269055 rs6746533	T	0.37	0.17	0.04	FSHR	Right putamen	2.22 × 10^−4^
				0.17	0.04		Right anterior putamen	2.08 × 10^−4^

3	NeuroX_rs3804958	A	0.30	−0.18	0.05	GRM7	Right anterior putamen	2.55 × 10^−4^

3	exm2269501 rs2289746	T	0.35	0.20	0.04	CBLB	Right caudate	8.57 × 10^−6^
				0.18	0.04		Left caudate	6.12 × 10^−5^
				0.19	0.04		Right putamen	4.00 × 10^−5^
				0.20	0.04		Left putamen	2.13 × 10^−5^
				0.21	0.04		Right anterior putamen	1.20 × 10^−5^
				0.22	0.04		Left anterior putamen	7.47 × 10^−6^

3	exm-rs9657904	C	0.21	0.20	0.05	CBLB	Right caudate	1.05 × 10^−4^

4	NeuroX_rs3736087	A	0.19	−0.22	0.05	GAK	Left caudate	1.37 × 10^−4^
				−0.23	0.05		Left putamen	7.97 × 10^−5^
				−0.23	0.06		Left anterior putamen	9.90 × 10^−5^

4	NeuroX_rs3775119	A	0.17	−0.21	0.06	GAK	Left caudate	2.66 × 10^−4^
				−0.24	0.06		Left putamen	6.66 × 10^−5^
				−0.23	0.06		Left anterior putamen	1.35 × 10^−4^

6	NeuroX_rs2798641	T	0.20	0.19	0.05	ARMC2	Right caudate	2.34 × 10^−4^
				0.20	0.05		Right anterior putamen	2.47 × 10^−4^

6	exm563164 rs4706980	A	0.09	0.29	0.07	DOP1A	Left putamen	1.68 × 10^−4^
				0.29	0.08		Left anterior putamen	2.43 × 10^−4^

7	NeuroX_rs12704813	G	0.26	0.18	0.04	PDK4-DYNC1I1	Right caudate	1.05 × 10^−4^

7	NeuroX_rs12704814	A	0.24	0.19	0.04	PDK4-DYNC1I1	Right caudate	8.65 × 10^−5^
				0.18	0.04		Left caudate	1.85 × 10^−4^

8	exm692355 rs3779620	C	0.11	0.27	0.07	PBK	Right caudate	1.04 × 10^−4^
				0.27	0.07		Right putamen	1.58 × 10^−4^
				0.27	0.07		Left putamen	1.95 × 10^−4^
				0.31	0.07		Right anterior putamen	2.50 × 10^−5^

8	exm717867 rs4870723	C	0.47	−0.17	0.04	COL14A1	Right caudate	1.12 × 10^−4^
				−0.17	0.04		Left caudate	9.30 × 10^−5^
				−0.17	0.04		Right putamen	1.87 × 10^−4^
				−0.18	0.04		Right anterior putamen	6.64 × 10^−5^
				−0.17	0.04		Left anterior putamen	2.01 × 10^−4^

8	exm732337 rs7830832	A	0.43	−0.16	0.04	TONSL	Left putamen	2.45 × 10^−4^

8	exm732383 rs2229315	C	0.44	−0.16	0.04	TONSL	Left putamen	1.79 × 10^−4^

8	exm-rs10481151	C	0.09	−0.32	0.07	FAM91A1-FER1L6	Right putamen	2.56 × 10^−5^
				−0.33	0.07		Right anterior putamen	1.95 × 10^−5^

8	exm692255 rs4732748	T	0.10	0.28	0.07	ESCO2	Right anterior putamen	2.21 × 10^−4^

11	exm959224 rs80264810	A	0.06	0.34	0.08	TMPRSS13	Right caudate	9.34 × 10^−5^

11	exm2271458 rs1384510	C	0.31	0.18	0.04	OR5P3-OR10A6	Left caudate	1.15 × 10^−4^

11	NeuroX_rs1349820	A	0.22	0.21	0.05	DLG2	Left caudate	8.07 × 10^−5^

12	exm2264471 rs7314355	C	0.44	−0.17	0.04	SLC16A7-TAFA2	Right anterior putamen	2.68 × 10^−4^

13	exm-rs9536591	C	0.48	0.16	0.04	LINC00458	Left caudate	1.30 × 10^−4^

13	exm2267525 rs9568968	A	0.47	0.15	0.04	LINC00458	Left caudate	2.11 × 10^−4^

13	exm-rs1413191	T	0.19	0.20	0.05	GPC5	Right putamen	2.38 × 10^−4^
				0.21	0.05		Right anterior putamen	2.30 × 10^−4^

17	exm2268030 rs714407	A	0.16	0.23	0.05	PIK3R5	Left caudate	7.95 × 10^−5^
				0.22	0.06		Left anterior putamen	2.01 × 10^−4^

17	exm1278142 rs222747	C	0.24	−0.19	0.05	TRPV1	Right putamen	1.64 × 10^−4^
				−0.21	0.05		Left putamen	2.55 × 10^−5^
				−0.19	0.05		Right anterior putamen	2.06 × 10^−4^
				−0.22	0.05		Left anterior putamen	2.55 × 10^−5^

17	exm1278123 rs224534	A	0.36	−0.17	0.04	TRPV1	Left putamen	2.07 × 10^−4^
				−0.17	0.04		Left anterior putamen	1.90 × 10^−4^

17	exm2260619 rs12450112	G	0.27	0.22	0.05	MAP2K4-MYOCD	Right caudate	7.36 × 10^−6^
				0.24	0.05		Right putamen	3.86 × 10^−6^
				0.19	0.05		Left putamen	1.72 × 10^−4^
				0.25	0.05		Right anterior putamen	1.34 × 10^−6^
				0.20	0.05		Left anterior putamen	1.07 × 10^−4^

19	exm1426190 rs3087689	G	0.27	0.21	0.05	KRI1	Left caudate	3.30 × 10^−5^
				0.20	0.05		Left anterior putamen	2.35 × 10^−4^

19	exm1511688 rs11667052	T	0.23	−0.20	0.05	ZNF471	Left caudate	1.28 × 10^−4^

19	exm1511712 rs3752176	A	0.44	−0.16	0.04	ZNF471	Right caudate	2.10 × 10^−4^
				−0.18	0.04		Left caudate	2.69 × 10^−5^
				−0.18	0.04		Left anterior putamen	1.08 × 10^−4^

19	exm1454888 rs2651079	T	0.48	0.16	0.04	ZNF792	Left putamen	2.36 × 10^−4^

20	exm2264705 rs527158	A	0.42	0.17	0.04	RAB22A	Left putamen	1.58 × 10^−4^

**Table 3:** Top eQTLs in one of three PD-related tissues (substantia nigra, caudate nucleus, and putamen nucleus) with their positional and functional mapped genes.

Chr	SNP/indel	Mapped gene	Top gene with significant eQTL *P* value
1	exm1952 rs78555129	C1QTNF12	ANKRD65 and B3GALT6

1	exm1971 rs12093154	C1QTNF12	ANKRD65 and B3GALT6

1	exm156096 rs1188732	OBSCN	OBSCN and WNT3A

1	exm2250840 rs12035900	OBSCN	OBSCN and WNT3A

4	NeuroX_rs3736087	GAK	IDUA

4	NeuroX_rs3775119	GAK	IDUA

6	exm563164 rs4706980	DOP1A	UBE3D

8	exm692355 rs3779620	PBK	CCDC25

8	exm692255 rs4732748	ESCO2	CCDC25

8	rs2280838	SLC39A4	VPS28

9	rs15394	CTSV	GAS21LP2

9	rs62560873	GAS21LP2	GAS21LP2

9	rs16924211	GAS21LP2	GAS21LP2

19	exm1511712 rs3752176	ZNF471	ZNF471 and AC007228.11

19	exm1511688 rs11667052	ZNF471	ZNF471 and AC007228.11

19	exm1426190 rs3087689	KRI1	KRI1

**Table 4:** Some of the suggestive genes with their possible roles in Parkinson's disease.

Gene	Original role	Possible role in Parkinson's disease	Expression level in basal ganglia based on The Human Protein Atlas	References
*IGFN1*	Synaptic assembly in the muscular tissue	Autophagy and protein synthesis	Low	[24–27]
TMEM169	Integral component of the membrane	Intracellular trafficking	High	[22, 23, 28]
TMEM9	Lysosomal acidification	Autophagic degradation of damaged organelles and protein aggregates	High	[22, 29–31]
SLC13A4	Transmembrane transporter	Specific variants may be involved in early-onset Parkinson's disease	High	[32, 33]
SLC39A4	Transmembrane transporter	Specific variants may be involved in early-onset Parkinson's disease	Low	[32, 33]
CACNA1A	Calcium channel	Trinucleotide expansion in CACNA1A causes slowly progressive, late-onset cerebellar ataxia, and parkinsonism	High	[34, 35]
OGDH	Krebs cycle	Its loss impairs autophagy and leads to neurodegeneration	High	[36, 37]
TBC1D15	Mitochondria-lysosome contact and autophagy	Impaired autophagy and mitochondria-lysosome contact	High	[38, 39]
F13A1	Coagulation	F13A1 was significantly associated with Alzheimer's disease, MCI, and Parkinson's disease, without a clear mechanism	Low	[40, 41]
CD80	A costimulatory molecule in T cell activation	Promoting T cell infiltration, inflammation, and neurodegeneration	Low	[42, 43]
CBLB	Proto-oncogene	It prevents JNK-mediated neuronal death	High	[44, 45]
*FECH*	Heme synthesis by adding ferrous to protoporphyrin IX	Heme can form complexes with *α*-synuclein, and excessive heme can cause neurodegeneration	High	[46–48]
GAK	Regulating clathrin-coated vesicle trafficking and *α*-synuclein overexpression	Maybe, by regulating vesicle trafficking and *α*-synuclein expression	High	[49–51]
KIF25	A microtubule-dependent motor protein	Impaired microtubule trafficking can lead to mitochondrial dysfunction and neurodegeneration	High	[52–54]
SAMD12	Involved in transmembrane receptor protein tyrosine kinase signaling pathway	N/A	High	[55, 56]
ZNF586	Involved in protein-protein and protein-nucleic acid interaction	Maybe, involved in neurodegenerative diseases	High	[57, 58],
CYP2A6	Metabolizing nicotine	Accelerating neurodegeneration by removing nicotine	Low	[59–61]
DACT3	Inhibition of autophagy and WNT/catenin signaling pathway	Inhibiting *α*-synuclein removal and contributing to neurodegeneration	High	[62–65]
GALNT8	O-linked glycosylation	Maybe, in neurogenesis and neurodegeneration	High	[66–68]
*DLG2*	Involved in synaptic function	Maybe, accelerates motor symptoms	Moderate-to-high	[69, 70]
GPC5	Cell membrane proteoglycan involved in growth and cell division	Maybe, differentiation of dopaminergic neurons	High	[71]
TRPV1	A cell surface receptor and ion channel	*α*-Synuclein removal and prevention of neurodegeneration	High	[72, 73]
GRM7	Involved in glutamatergic neurotransmission	Improving motor dysfunction in PD	High	[74, 75]

## Data Availability

Data from the PPMI cohort were used for analysis in this study. Data can be obtained from their website.

## Supplementary Materials

Supplementary Materials Supplementary Material 1: the detailed protocol of PPMI cohort. Supplementary Material 2: Q-Q plot and Manhattan plot of right putamen, right caudate, right anterior putamen, left putamen, left caudate, and left anterior putamen DaTscan EWAS. Supplementary Material 3: Q-Q plot and Manhattan plot of right putamen, right caudate, right anterior putamen, left putamen, left caudate, and left anterior putamen DaTscan GWAS.
