# Supplementary material for: Genome- and Exome-Wide Association Studies Revealed Candidate Genes Associated with DaTscan Imaging Features
Source: Parkinsons Dis. 2023 Aug 23;2023:2893662. doi: 10.1155/2023/2893662 (PMC10468272; doi:10.1155/2023/2893662)

DocuSign Envelope ID: 706F2DD8-8346-4731-A419-FF8E7C48DF97

PROTOCOL

Title: The Parkinson’s Progression Markers Initiative (PPMI) Clinical -

Establishing a Deeply Phenotyped PD Cohort

Sponsor: Michael J. Fox Foundation

Principal Investigators: Kenneth Marek, MD

Caroline Tanner, MD

Protocol Number:

Date of Protocol:

002

February 1, 2021

Final Version: 1.2

PPMI

Version Date: 01Feb2021

Page 1 of 55


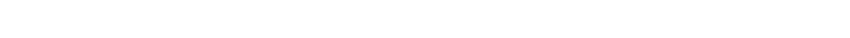


DocuSign Envelope ID: 706F2DD8-8346-4731-A419-FF8E7C48DF97

PROTOCOL APPROVAL

Amendment 2, Version 1.2 dated February 1, 2021

The Parkinson’s Progression Markers Initiative (PPMI) Clinical

2/3/2021

Kenneth Marek, MD

Principal Investigator

Date

2/3/2021

Sohini Chowdhury

Date

Michael J Fox Foundation (Sponsor)

PPMI

Page 2 of 55

Version Date: 01Feb2021


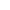

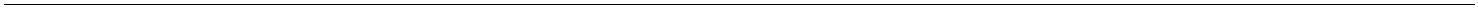

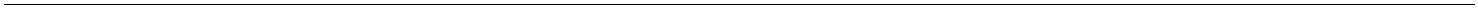

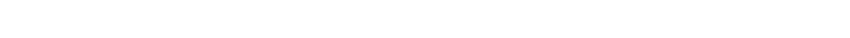

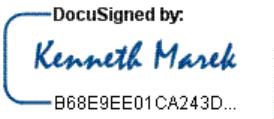

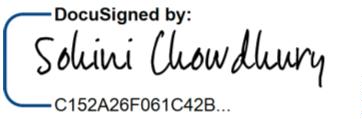


DocuSign Envelope ID: 706F2DD8-8346-4731-A419-FF8E7C48DF97

TABLE OF CONTENTS

[PROTOCOL APPROVAL .......................................................................................................... 2](#br2)

[1](#br6)

[PURPOSE OF STUDY............................................................................................... 6](#br6)

[1](#br6)

[1](#br7)

[.1](#br6)

[.2](#br7)

[Primary Objectives of PPMI Clinical.......................................................................... 6](#br6)

[Secondary Objectives.................................................................................................. 7](#br7)

[2](#br7)

[3](#br7)

[STUDY OUTCOMES................................................................................................. 7](#br7)

[BACKGROUND AND RATIONALE ....................................................................... 7](#br7)

[3](#br7)

[3](#br10)

[.1](#br7)

[.2](#br10)

[Background for PPMI Clinical.................................................................................... 7](#br7)

[Rationale for PPMI Clinical...................................................................................... 10](#br10)

[4](#br10)

[5](#br11)

[6](#br11)

[STUDY DESIGN...................................................................................................... 10](#br10)

[STUDY COHORTS.................................................................................................. 11](#br11)

[RECRUITMENT METHODS .................................................................................. 11](#br11)

[6](#br12)

[6](#br12)

[.1](#br12)

[.2](#br12)

[Prodromal Participants – Path to PPMI Clinical....................................................... 12](#br12)

[Identifying Participants with Genetic Variants ......................................................... 12](#br12)

[7](#br13)

[PARTICIPANT ELIGIBLITY.................................................................................. 13](#br13)

[7](#br13)

[7](#br14)

[7](#br15)

[7](#br16)

[7](#br17)

[.1](#br13)

[.2](#br14)

[.3](#br15)

[.4](#br16)

[.5](#br17)

[Healthy Controls (HC) .............................................................................................. 13](#br13)

[Parkinson Disease (PD)............................................................................................. 14](#br14)

[Parkinson Disease (PD) with LRRK2 or GBA mutation.......................................... 15](#br15)

[Parkinson Disease (PD) with SNCA or rare genetic variant..................................... 16](#br16)

[Prodromal .................................................................................................................. 17](#br17)

[8](#br18)

[OBTAINING INFORMED CONSENT ................................................................... 18](#br18)

[8](#br18)

[8](#br19)

[8](#br20)

[8](#br20)

[.1](#br18)

[.2](#br19)

[.3](#br20)

[.4](#br20)

[Consent Process......................................................................................................... 18](#br18)

[Identification of Research Proxy............................................................................... 19](#br19)

[Permission to be Contacted for Follow Up of Persons with Neurologic Disease..... 20](#br20)

[Permission to be Contacted from Pathology Core .................................................... 20](#br20)

[9](#br21)

[1](#br22)

[PARTICIPANT INFORMATION AND STUDY ID............................................... 21](#br21)

[9](#br21)

[9](#br21)

[9](#br21)

[.1](#br21)

[.2](#br21)

[.3](#br21)

[Participant Profile Information.................................................................................. 21](#br21)

[Participant ID Number .............................................................................................. 21](#br21)

[Globally Unique Identifier (GUID) Number............................................................. 21](#br21)

[0](#br22)

[STUDY VISIT PROCEDURES ............................................................................... 22](#br22)

[1](#br22)

[1](#br23)

[1](#br24)

[1](#br26)

[1](#br27)

[1](#br27)

[1](#br27)

[0.1](#br22)

[0.2](#br23)

[0.3](#br24)

[0.4](#br26)

[0.5](#br27)

[0.6](#br27)

[0.7](#br27)

[Active PPMI Transitioning Participants.................................................................... 22](#br22)

[Healthy Control, PD and PD Genetic Cohort Visits ................................................. 23](#br23)

[Prodromal Cohort Visits............................................................................................ 24](#br24)

[Need for PD Therapy Visit........................................................................................ 26](#br26)

[Premature Withdrawal Visit...................................................................................... 27](#br27)

[Unscheduled Visits.................................................................................................... 27](#br27)

[Out of Clinic Annual Visits....................................................................................... 27](#br27)

PPMI

Version Date: 01Feb2021

Page 3 of 55


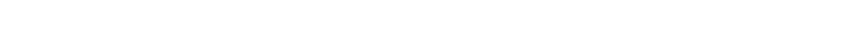


DocuSign Envelope ID: 706F2DD8-8346-4731-A419-FF8E7C48DF97

[1](#br28)

[1](#br28)

[1](#br28)

[2](#br28)

[CLINICAL ASSESSMENTS.................................................................................... 28](#br28)

[SAFETY ASSESSMENTS....................................................................................... 28](#br28)

[1](#br28)

[1](#br28)

[1](#br28)

[2.1](#br28)

[2.2](#br28)

[2.3](#br28)

[Medical Conditions Review, Physical and Neurological Examination .................... 28](#br28)

[Vital Signs/Weight/Height ........................................................................................ 28](#br28)

[Clinical Laboratory Tests .......................................................................................... 28](#br28)

[1](#br29)

[3](#br29)

[BIOLOGIC RESEARCH SAMPLING..................................................................... 29](#br29)

[1](#br29)

[1](#br29)

[1](#br29)

[1](#br30)

[3.1](#br29)

[3.2](#br29)

[3.3](#br29)

[3.4](#br30)

[Blood Samples........................................................................................................... 29](#br29)

[Urine.......................................................................................................................... 29](#br29)

[Lumbar Puncture / Cerebral Spinal Fluid (CSF)....................................................... 29](#br29)

[Skin Biopsy ............................................................................................................... 30](#br30)

[1](#br30)

[1](#br31)

[4](#br30)

[IMAGING ................................................................................................................. 30](#br30)

[1](#br30)

[1](#br31)

[4.1](#br30)

[4.2](#br31)

[Dopamine Transporter SPECT Imaging ................................................................... 30](#br30)

[Magnetic Resonance Imaging (MRI)........................................................................ 31](#br31)

[5](#br31)

[RISKS TO PARTICIPANTS.................................................................................... 31](#br31)

[1](#br31)

[1](#br31)

[1](#br31)

[1](#br32)

[1](#br32)

[1](#br32)

[5.1](#br31)

[5.2](#br31)

[5.3](#br31)

[5.4](#br32)

[5.5](#br32)

[5.6](#br32)

[Blood Sampling......................................................................................................... 31](#br31)

[MRI ........................................................................................................................... 31](#br31)

[DaTscan Imaging ...................................................................................................... 31](#br31)

[Lumbar Puncture ....................................................................................................... 32](#br32)

[Skin Biopsy ............................................................................................................... 32](#br32)

[Disclosure of Genetic Information............................................................................ 32](#br32)

[1](#br32)

[1](#br33)

[1](#br33)

[6](#br32)

[REFERRALS IN THE CASE OF CLINICALLY RELEVANT FINDINGS .......... 32](#br32)

[POTENTIAL BENEFITS TO PARTICIPANTS...................................................... 33](#br33)

[CONCOMITANT MEDICATIONS......................................................................... 33](#br33)

[7](#br33)

[8](#br33)

[1](#br33)

[1](#br33)

[8.1](#br33)

[8.2](#br33)

[Use of Concomitant Medications.............................................................................. 33](#br33)

[Initiation of PD Medication....................................................................................... 33](#br33)

[1](#br33)

[2](#br33)

[2](#br34)

[2](#br34)

[2](#br34)

[9](#br33)

[PARTICIPATION IN CLINICAL TRIALS............................................................. 33](#br33)

[COSTS FOR PARTICIPATION............................................................................... 33](#br33)

[PAYMENT AND REIMBURSEMENT FOR PARTICIPATION........................... 34](#br34)

[PARTICIPANT WITHDRAWALS.......................................................................... 34](#br34)

[ADVERSE EVENTS ................................................................................................ 34](#br34)

[0](#br33)

[1](#br34)

[2](#br34)

[3](#br34)

[2](#br34)

[3.1](#br34)

[3.2](#br34)

[3.3](#br35)

[3.4](#br36)

[3.5](#br36)

[Adverse Event Reporting Requirements ................................................................... 34](#br34)

[Serious Adverse Event Reporting Requirements ...................................................... 34](#br34)

[Adverse Event Definitions ........................................................................................ 35](#br35)

[Assessing Relationship of Adverse Events ............................................................... 36](#br36)

[Assessing Intensity/Severity of Adverse Event......................................................... 36](#br36)

[2](#br34)

[2](#br35)

[2](#br36)

[2](#br36)

[2](#br36)

[2](#br37)

[4](#br36)

[SIGNIFICANT STUDY EVENTS ........................................................................... 36](#br36)

[STUDY MONITORING AND SITE MANAGEMENT.......................................... 37](#br37)

[5](#br37)

PPMI

Version Date: 01Feb2021

Page 4 of 55


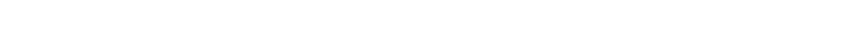


DocuSign Envelope ID: 706F2DD8-8346-4731-A419-FF8E7C48DF97

[2](#br37)

[2](#br37)

[2](#br38)

[6](#br37)

[7](#br37)

[8](#br38)

[PRIVACY AND CONFIDENTIALITY................................................................... 37](#br37)

[DATA AND SAMPLE SHARING AND STORAGE FOR FUTURE USE............ 37](#br37)

[ANALYSIS PLAN.................................................................................................... 38](#br38)

[2](#br38)

[2](#br39)

[8.1](#br38)

[8.2](#br39)

[Primary Objectives.................................................................................................... 38](#br38)

[Secondary Objectives................................................................................................ 39](#br39)

[2](#br41)

[3](#br44)

[3](#br46)

[3](#br48)

[3](#br50)

[3](#br52)

[3](#br54)

[9](#br41)

[REFERENCES.......................................................................................................... 41](#br41)

[APPENDIX 1 – Healthy Control Schedule Years 0-5.............................................. 44](#br44)

[APPENDIX 2 – Prodromal Schedule Years 0 – 5 .................................................... 46](#br46)

[APPENDIX 3 – PD / PD Genetic Schedule Years 0 – 5 .......................................... 48](#br48)

[APPENDIX 4 – Healthy Control Schedule Years 6-13............................................ 50](#br50)

[APPENDIX 5 – Prodromal Schedule Years 6-13..................................................... 52](#br52)

[APPENDIX 6 - PD / PD Genetic Schedule Years 6 - 13.......................................... 54](#br54)

[0](#br44)

[1](#br46)

[2](#br48)

[3](#br50)

[4](#br52)

[5](#br54)

PPMI

Version Date: 01Feb2021

Page 5 of 55


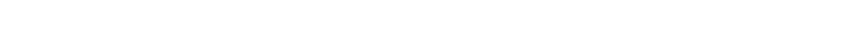


DocuSign Envelope ID: 706F2DD8-8346-4731-A419-FF8E7C48DF97

1

PURPOSE OF STUDY

The Parkinson Progression Marker Initiative (PPMI) is a longitudinal, observational, multi-

center natural history study to assess progression of clinical features, digital outcomes, and

imaging, biologic and genetic markers of Parkinson’s disease (PD) progression in study

participants with manifest PD, prodromal PD, and healthy controls. The overall goal of

PPMI is to identify markers of disease progression for use in clinical trials of therapies to

reduce progression of PD disability.

PPMI is a broad program, expanding the goals of the original PPMI study, that includes this

PPMI Clinical protocol, as well as the PPMI Remote, PPMI Digital App and PPMI Online

protocols. Participants in PPMI may be asked to be enrolled in other PPMI program

protocols, but depending on their method of recruitment, participants may be enrolled

sequentially in varying order, as appropriate. PPMI participants may also be asked to

participate in additional PPMI companion studies (as they are developed), which may only

involve a subset of PPMI participants based on their cohort designation and/or site location.

1

.1 Primary Objectives of PPMI Clinical

The primary objectives include to:

a. Establish standardized protocols for acquisition, transfer and analysis of clinical, digital,

imaging, biologic and genetic data that can be used by the PD research community. This

protocol will build on the existing PPMI infrastructure.

b. Develop a comprehensive and uniformly acquired clinical, digital and imaging dataset

and repository of biological and genetic samples that would be available to the PD

research community to test hypotheses of the underlying molecular pathobiology of PD,

enable modeling of PD progression to identify clinical and/or data driven PD

progression sub-sets, and inform studies testing PD therapeutics (for examples, clinical

trials targeting synuclein, LRRK2, GBA as well as other targets)

c. Use clinical and biological data to estimate the mean rates of change and the variability

around the mean of clinical, digital, imaging, biological and genetic outcomes in study

participants with PD diagnosis (including patients with a LRRK2, GBA, SNCA or rare

genetic mutations (such as Parkin or Pink1) and individuals with prodromal Parkinson

disease (including individuals with RBD, olfactory loss, LRRK2, GBA, SNCA or rare

genetic variants (such as Parkin or Pink1) and/or other risk factors for PD with and

without dopamine transporter (DAT) deficit and in healthy participants.

d. Confirm existing and identify novel clinical, digital, imaging, biologic and genetic PD

progression markers to identify quantitative individual measures or combinations of

measures that demonstrate optimum interval change in study participants with PD

diagnosis (including patients with a LRRK2, GBA, SNCA or rare genetic variants (such

as Parkin or Pink1)) and individuals with prodromal Parkinson disease (including

individuals with RBD, olfactory loss, a LRRK2, GBA, SNCA or rare genetic variants

(such as Parkin or Pink1) and/or other risk factors for PD with and without DAT deficit

in comparison to healthy controls or in sub-sets of study participants with PD diagnosis

or prodromal PD defined by baseline assessments, progression milestones and/or rate of

clinical, digital, imaging, biologic and genetic change, or other measures.

e. Evaluate the probability of phenoconversion to PD for individuals with prodromal PD

enrolled in the prodromal cohorts (including individuals with RBD, olfactory loss, a

PPMI

Page 6 of 55

Version Date: 01Feb2021


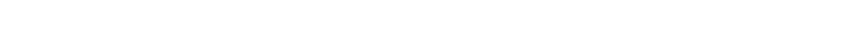


DocuSign Envelope ID: 706F2DD8-8346-4731-A419-FF8E7C48DF97

LRRK2, GBA, SNCA or rare genetic variants (such as Parkin or Pink1) and/ or other

risk factors for PD with and without DAT deficit).

1

.2 Secondary Objectives

The secondary objectives include the following:

a. Conduct preliminary clinical, digital, imaging, biologic and genetic markers verification

studies on promising biological markers in study subsets and/or using stored collected

samples.

b. Compare biomarker signatures for study participants with PD diagnosis without known

genetic mutation to those with known genetic mutation (including LRRK2, GBA,

SNCA or rare genetic variants (such as Parkin or Pink1)).

c. Compare biomarker signatures in study participants with PD diagnosis to individuals

with prodromal PD enrolled in the prodromal cohorts (including individuals with RBD,

olfactory loss, LRRK2, GBA, SNCA or rare genetic variants (such as Parkin or Pink1)

and/or other risk factors for PD with and without DAT deficit).

d. Compare biomarker signature between prodromal PD subsets including individuals with

RBD, olfactory loss, LRRK2, GBA, SNCA or rare genetic variants (such as Parkin or

Pink1) and/or other risk factors for PD with and without DAT deficit.

e. Develop and test risk paradigms to establish the sequence of early prodromal events

(clinical, imaging, biologic changes) in individuals with prodromal PD enrolled in the

prodromal cohorts (including individuals with RBD, olfactory loss, LRRK2, GBA,

SNCA or rare genetic variants (such as Parkin or Pink1) and/or other risk factors for PD

with and without DAT deficit) including testing early signal of risk in the associated

PPMI Online and PPMI Remote studies.

2

STUDY OUTCOMES

Key PPMI outcomes will be longitudinal change in clinical (motor and non-motor) scales

(e.g., MDS-UPDRS, MoCA), Patient Reported Outcomes (PROs) and digital outcomes,

quantitative imaging (DAT, SBR, and MRI midbrain melanin), and biologic measures of

synuclein, lysosomal function, and analytes related to neurodegeneration (e.g., neurofilament

light chain inflammation). Detailed demographic, clinical and biological data will be

collected to test specific hypotheses in subsequent analyses and other associated protocols.

In addition, data quality metrics including compliance with study procedures, quality metrics

related to biosamples and completeness of data collection will be monitored on an ongoing

basis.

3

BACKGROUND AND RATIONALE

3

.1 Background for PPMI Clinical

The defining motor features of Parkinson disease (PD) are characterized by their insidious

onset and inexorable but heterogenous progression. Reliable and well-validated biomarkers

to monitor PD progression would dramatically accelerate research into both PD etiology and

therapeutics. Much progress has been made in identifying and assessing PD biomarkers,

and yet no fully validated biomarker or set of biomarkers for PD are currently available.

Nonetheless there is increasing evidence that assessment of clinical, digital, imaging

outcomes and measurement of analytes from blood, cerebral spinal fluid (CSF), urine, and

PPMI

Page 7 of 55

Version Date: 01Feb2021


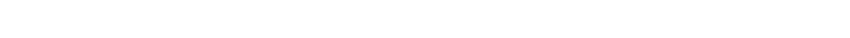


DocuSign Envelope ID: 706F2DD8-8346-4731-A419-FF8E7C48DF97

tissue has already begun to provide crucial tools for PD drug development and for

understanding the pathobiology of PD (1-3).

During the past decade, the PPMI study has established a longitudinal clinical and biomarker

data resource on approximately 1,500 participants including cohorts with idiopathic PD, PD

with genetic mutations, prodromal participants and healthy controls. PPMI is an

observational, international, multi-center study designed to establish biomarker defined

cohorts and to identify PD progression biomarkers to improve understanding of disease

etiology and course and to provide critical tools to enhance the likelihood of success of PD

therapeutic trials [(ClinicalTrials.gov](https://clinicaltrials.gov/ct2/home) NCT01141023). PPMI is a collaborative effort of PD

researchers with expertise in biomarker development, PD clinical study design and

implementation, bioinformatics, statistics, and data management. The study is a public-

private partnership of academic researchers, the Michael J Fox Foundation (MJFF) and

pharmaceutical, biotech, government and foundation partners.

The overall goal of PPMI is to examine clinical, imaging, genetic and biospecimen PD

progression markers that individually or in combination will rapidly demonstrate interval

change in PD patients in comparison to Healthy controls (HC) or in sub-sets of PD patients

defined by baseline assessments, genetic mutations, progression milestones and/or rate of

clinical, imaging or biospecimen change. PPMI has established standardized protocols for

acquisition, transfer and analysis of clinical, imaging, genetic and biospecimen data that can

be used by the PD research community. Importantly PPMI is committed to data and

biospecimen sharing. PPMI data are available to the research community on the PPMI

website as it is collected and there have been more than five million downloads of PPMI

data (as of Dec 2019). PPMI biospecimens are available by application to the PPMI

Biospecimen review committee with more than three hundred requests, as of December

2

019. All PPMI standardized protocols and PPMI data are available at [http://www.ppmi-](http://www.ppmi-info.org/)

[info.org](http://www.ppmi-info.org/) (4, 5).

PPMI is the most comprehensive natural history dataset of PD participants and serves,

according to its original purpose, as a key resource for drug development and understanding

of the clinical and biological features of PD progression. The study has demonstrated the

enormous value of comprehensive, longitudinal within subject biomarker assessment. PPMI

has developed a robust study infrastructure with well-developed study leadership and

governance, committed enrolling sites, and expert study cores (data, imaging,

biorepositories, bioinformatics, genetics) to ensure the ongoing collection and analysis of

study data. The study has developed and expanded methods to enroll biomarker defined

cohorts requiring dopamine imaging deficit for inclusion in the PD cohort, piloted methods

to establish prodromal cohorts of hyposmic and RBD participants, and has established a

novel centralized strategy to enroll participants with PD genetic mutations (6-8). PPMI has

also demonstrated the feasibility and safety of multicenter longitudinal collection of CSF

(9).

PPMI longitudinal data has and continues to be acquired and reported to inform clinical trials

for PD. PPMI data has detailed the progression of the MDS-UPDRS (both off and on PD

meds) and cognitive and behavioral outcomes enabling sample size estimation to detect

PPMI

Version Date: 01Feb2021

Page 8 of 55


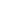

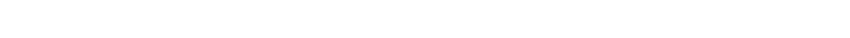


DocuSign Envelope ID: 706F2DD8-8346-4731-A419-FF8E7C48DF97

changes in progression due to therapeutic intervention (10-12), Predictors of key PPMI

outcomes and of need for PD therapy have also been evaluated (13, 14). Progression of

dopamine transporter (DAT) imaging has demonstrated a robust reduction in PD participants

and PPMI DAT eligibility data has contributed to its qualification by the EMA as an

enrichment biomarker (11, 15, 16). Longitudinal analysis of synuclein, amyloid and tau

from CSF has demonstrated a persistent reduction in synuclein and tau in PD participants

compared to healthy controls but without significant progression (17-19). Several other

analytes/pathways have also been assessed including neurofilament light chain,

catecholamines, and the lysosomal pathway providing additional data ([PPMI website)](https://www.ppmi-info.org/)

showing modest changes with progression with reports in press.

A key strength of PPMI is the within participant design so that multiple biomarkers are

assessed in each participant. This strategy has enabled studies identifying PD subsets based

several biomarkers and has allowed various multi-modal biomarkers to be compared. There

have been several efforts to develop biomarker derived subsets of PD to define risk and/or

disease progression to further understand the heterogeneity of PD. Combining genetics with

clinical and imaging biomarkers has resulted in a genetic risk score that may be helpful in

predicting PD and has led to additional studies combining whole genome sequencing, RNA

transcriptomics and clinical and imaging markers (20). DAT and MRI imaging have been

combined with clinical outcomes to explore PD subsets and PD pathobiology (21-23).

Combining clinical motor outcomes with behavioral and cognitive outcomes has provided

insight to the timing of non-motor PD disability and utility of current non-motor scales to

track early disease (24-27). Examining biomarkers in genetic cohorts has further identified

specific imaging and biologic markers that may distinguish those cohorts (28, 29). PPMI

offers the opportunity to examine multiple biomarker data streams and analysis strategies

for these data including unbiased data analysis approaches have identified possible PD

subsets and predictors of progression (30).

PPMI has also developed prodromal cohorts defined by olfaction, RBD or genetic mutation

to pilot longitudinal assessment of biomarker prodromal PD and establish biomarkers that

predict the development of motor parkinsonism. Overwhelming scientific data have

demonstrated that the molecular pathology of Parkinson’s disease begins long in advance of

clinical symptoms. Longitudinal densely phenotyped follow-up of individuals at high risk

to develop PD would enable both understanding of the progression of disease during the

prodromal period and could ultimately lead to the testing of therapies that might prevent the

onset of manifest motor PD. The Movement Disorder Society proposed criteria to define

prodromal PD for research (31, 32). Prior studies including the Parkinson Associated Risk

Syndrome (PARS) study and long-term RBD studies have further demonstrated that

prodromal PD participants with hyposmia or RBD with abnormal imaging have high risk of

the onset of motor PD within 3-5 years (33, 34). Pilot prodromal data from the ongoing

PPMI study has shown that about 35% of hyposmic and RBD participants with abnormal

DAT converted to motor PD within four years. Data from the unaffected LRRK2 and GBA

mutation carriers shows less than 10 % of participants with abnormal DAT, but mild increase

in motor and non-motor features compared to healthy subjects (35).

PPMI

Version Date: 01Feb2021

Page 9 of 55


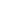

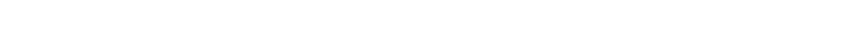


DocuSign Envelope ID: 706F2DD8-8346-4731-A419-FF8E7C48DF97

PPMI has been committed to open source data with rapid sharing of all PPMI data to the PD

community (36) . This data resource now includes clinical (motor and non-motor), digital,

imaging, and genetic data plus a robust biorepository including blood, CSF, urine and

induced pluripotent stem (IPS) cells.

3

.2 Rationale for PPMI Clinical

While the PPMI study has made substantial progress as outlined above, the program offers

the opportunity to expand and transform the use of biomarkers to test hypotheses of the

underlying molecular pathobiology of PD, enable modeling of PD progression to identify

clinical and/or biologic data driven PD progression sub-sets, and inform studies testing PD

therapeutics including clinical trials targeting synuclein, LRRK2, GBA and other targets.

There is a consensus that a new PPMI cohort is necessary to further develop and validate

biomarkers for PD progression and prodromal PD to enable therapeutic development.

Further advances in molecular genetics, neurobiology, imaging technology, wearable sensor

and remote assessment technology and radiochemistry have provided new tools that may be

useful in identification of such biomarkers for further studies of therapies that may slow or

prevent PD disability. The goal of this new initiative is to extend the current PPMI

consortium of academic centers, PD foundations, pharmaceutical and biotech companies.

government agencies, and active study participants, to establish and validate markers of PD

progression across the spectrum of disease from prodromal PD to more advanced disease.

In PPMI Clinical, established tools will also be further validated and new technologies

including neuroimaging modalities, digital biomarkers, biochemical markers in the CSF and

plasma, genetic markers, and early clinical disease markers will be investigated. We will

continue to standardize biomarker acquisition and assessment and to establish well-defined

quantitative biomarker outcomes that are consistent among many research sites and

laboratories. Core laboratories for biomarker analysis will be used for uniformity of analyses

and quality control. A major focus of this biomarker consortium will be to extend PPMI

infrastructure to new biomarkers and new cohorts, particularly those with prodromal PD.

Longitudinal data will include participant reported outcomes with an emphasis on \outcomes

that reflect participant function throughout the course of PD

This approach to biomarker development is ambitious and requires collaboration among

many in academics, industry, government, and the public sector. However, PPMI has

demonstrated that such an approach is feasible. PPMI has been successful in providing open

source data and fostering effective collaboration. The unmet need for therapeutics that slow

or prevent the disability of PD coupled with the enormous value of biomarkers to enable and

accelerate clinical studies highlights the need for this strategy to identify and validate

biomarkers of PD progression throughout the course of disease.

4

STUDY DESIGN

PPMI Clinical is a longitudinal, observational, multi-center natural history study to assess

progression of clinical features, digital outcomes, and imaging, biologic and genetic markers

of PD progression in study participants with PD diagnosis (including patients with a LRRK2,

GBA, SNCA or rare genetic variants and individuals with prodromal Parkinson disease

(including individuals with RBD, olfactory deficit, LRRK2, GBA, SNCA or rare genetic

PPMI

Page 10 of 55

Version Date: 01Feb2021


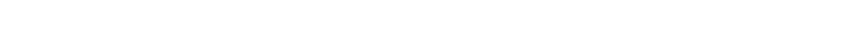


DocuSign Envelope ID: 706F2DD8-8346-4731-A419-FF8E7C48DF97

mutations (such as Parkin or Pink1) and/or other risk factors for PD with and without DAT

deficit and healthy controls.

All participants will be comprehensively assessed for a minimum of 5 years. Participants will

undergo clinical (motor, neuropsychiatric and cognitive) and imaging assessments, and will

donate biosamples including blood, urine, and cerebral spinal fluid (CSF) and skin biopsy.

Participants will also be asked to respond to targeted questionnaires and provide digital data

as part of the PPMI Online and PPMI Digital App protocols (under separate consent).

5

STUDY COHORTS

In PPMI Clinical up to 4,500 participants will be enrolled and followed longitudinally from

approximately 50-55 international clinical sites across a variety of cohorts as described below

(note that the cohorts enrolled might vary across sites).

1

. Current PPMI participants: All participants across all cohorts enrolled in PPMI will be

eligible to continue participation in PPMI. Participants may belong to one of the following

PPMI cohorts (healthy control, PD, PD with LRRK2 mutation, PD with GBA mutation,

PD with SNCA mutation or rare genetic variants, prodromal with LRRK2 mutation,

prodromal with GBA mutation, prodromal with SNCA mutation or rare genetic variants,

prodromal with hyposmia, prodromal with RBD). These participants will be invited to join

the cohort in PPMI that matches their original designation (n=up to 1150).

2

3

. Healthy controls (n=up to 120).

. Parkinson disease (PD) participants who are recently diagnosed and untreated (n= up to

7

00).

4

. PD manifesting gene carriers with a LRRK2 or GBA mutation (n=up to 250), SNCA, or

other rare genetic variant (such as Parkin or Pink1) (n= up to 60).

5

. Prodromal PD (at risk for PD) (n=up to 2220)

5

.1.1 Hyposmia (generalized risk) (n= up to 1260)

.1.2 RBD (n= up to 500)

.1.3 LRRK2 mutation (n= up to 200)

.1.4 GBA mutation (n= up to 200)

5

5

5

5

.1.5 SNCA or other rare genetic variants (n= up to 60)

6

RECRUITMENT METHODS

Participants in PPMI Clinical with PD and healthy controls will largely be identified by study

sites. Prodromal participants, as well as participants having PD with a genetic mutation, will

largely be identified through other resources and will be referred to PPMI clinical sites to be

considered for enrollment in this study; however, these participants may also be recruited

directly through the clinical site. Recruitment of these cohorts is described further below.

PPMI

Page 11 of 55

Version Date: 01Feb2021


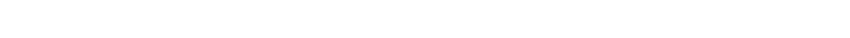


DocuSign Envelope ID: 706F2DD8-8346-4731-A419-FF8E7C48DF97

6

.1 Prodromal Participants – Path to PPMI Clinical

Potential prodromal participants may be eligible to participate in PPMI Clinical based on

participation in the PPMI Online protocol (information from online questionnaires assessing

general health and risk of PD), PPMI Remote (information from additional remote testing

including olfactory testing), or directly from a clinical site based on known PD risk, such as

possible REM behavior disorder or known genetic variants associated with PD risk.

Eligible individuals from PPMI Remote will be referred to PPMI clinical sites to consent to

participation in PPMI Clinical and undergo their prodromal DAT imaging screening visit.

Individuals identified by a clinical site with known risks may also be considered to consent

to participation in PPMI Clinical and undergo a Prodromal Screening visit.

All prodromal participants will be screened for hyposmia (UPSIT), either in PPMI Remote

or, if recruited by the clinical site, as part of the PPMI Clinical Prodromal Screening visit.

6

6

.2 Identifying Participants with Genetic Variants

Identifying participants with genetic variants will require very targeted recruitment.

.2.1 Prodromal participants with a genetic variant will be identified in large part through a

centralized recruitment process, as well as from clinical sites.

a) Centralized Recruitment – Indiana University

Individuals who are unaffected will be recruited by Indiana University centrally through

the PPMI screening process. Potential participants will be identified by digital targeting

using social media such as Facebook. These potential participants will undergo remote

screening and will undergo genetic testing (saliva kits) and telegenetic counseling (under

separate consent). These participants will be eligible to participate in other PPMI program

protocols, including PPMI Online, PPMI Remote, PPMI Digital App, and subsequently, if

eligible would be referred to a study site and further assessed for eligibility to enroll in

PPMI Clinical.

b) Clinical Site Recruitment of Unaffected Persons with Genetic Variants

Individuals who are unaffected may also be recruited from the clinical sites. In general,

these sites will have prior access to participants and families with genetic mutations due to

site interest and/or specific geographic location. Individuals being considered for

participation in PPMI Clinical at clinical sites who may have these genetic variants, but

have not previously undergone genetic testing, will undergo evaluation including genetic

testing and genetic counseling (under separate consent from the PPMI Clinical study).

Existing documentation of test results will be provided to the PPMI Genetic Coordination

Core for further review and confirmation of eligibility for inclusion in PPMI Clinical. If

approved, these participants would not require additional genetic testing. Individuals with

LRRK2, GBA, SNCA, or rare genetic variants would be referred back to the study site, or

another study site, and further assessed for eligibility to enroll in PPMI Clinical.

PPMI

Page 12 of 55

Version Date: 01Feb2021


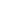

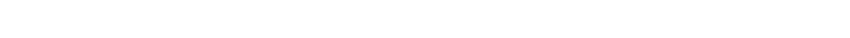


DocuSign Envelope ID: 706F2DD8-8346-4731-A419-FF8E7C48DF97

6

.2.2 Parkinson’s disease participants with a genetic mutation will be identified in large part

through a centralized recruitment process, as well as from clinical sites.

a) Centralized Recruitment – Indiana University

Individuals with PD and LRRK2 and/or GBA mutations will be recruited centrally by

Indiana University through the PPMI screening process. Potential participants will be

identified by digital targeting using social media such as Facebook. These potential

participants will undergo remote genetic testing (saliva kits) and telegenetic counseling

(under separate consent). If mutation positive, these participants will be referred to a study

site and further assessed for eligibility to enroll in PPMI Clinical. If mutation negative,

these participants will be referred to PPMI Online if not already enrolled.

b) Clinical Site Recruitment

Individuals with PD and LRRK2, GBA, SNCA, or rare genetic variants (such as Parkin or

Pink1 mutations), may also be recruited from the clinical sites. In general, these sites will

have prior access to participants and families with genetic mutations due to site experience

and/or specific geographic location. Individuals being considered for participation in

PPMI Clinical at clinical sites who may have these genetic variants, but have not previously

undergone genetic testing, will undergo evaluation including genetic testing and genetic

counseling (under separate consent from the PPMI Clinical study).

Existing

documentation of test results will be provided to the PPMI Genetic Coordination Core for

further review and confirmation of eligibility for inclusion in PPMI Clinical. If approved,

these participants would not require additional genetic testing. Individuals with LRRK2,

GBA, SNCA, or rare genetic variants would be referred back to the study site, or another

study site, and further assessed for eligibility to enroll in PPMI Clinical.

7

PARTICIPANT ELIGIBLITY

7

.1 Healthy Controls (HC)

Note: Active Healthy controls previously enrolled in PPMI do not require re-assessment of

eligibility criteria listed below for enrollment in PPMI Clinical. Active participants do need

to be able to provide informed consent for PPMI Clinical participation (includes use of a

designated research proxy).

7

.1.1. Inclusion Criteria (HC)

a) Male or female age 30 years or older at Screening visit.

b) Individuals taking any of the following drugs: alpha methyldopa, methylphenidate,

amphetamine derivatives or modafinil, must be willing and medically able to hold the

medication for at least 5 half-lives before DaTscan imaging.

c) Confirmation that participant is eligible based on Screening DaTscan imaging.

d) Able to provide informed consent.

e) Either is male, or is female and meets additional criteria below, as applicable:

•

Female of childbearing potential who is not pregnant, lactating, or planning

pregnancy during the study and has a negative pregnancy test on day of Screening

DaTscan imaging test prior to injection of DaTscan^TM^.

PPMI

Version Date: 01Feb2021

Page 13 of 55


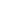

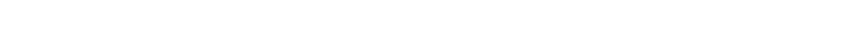


DocuSign Envelope ID: 706F2DD8-8346-4731-A419-FF8E7C48DF97

7

.1.2. Exclusion Criteria (HC)

a) First degree relative with PD (i.e., biologic parent, sibling, child).

b) Current or active clinically significant neurological disorder (in the opinion of the

Investigator).

c) Previously obtained MRI scan with evidence of clinically significant neurological

disorder (in the opinion of the Investigator).

d) Received any of the following drugs: dopamine receptor blockers (neuroleptics),

metoclopramide and reserpine within 6 months of Screening visit.

e) Current treatment with anticoagulants (e.g., coumadin, heparin, oral thrombin

inhibitors) that might preclude safe completion of the lumbar puncture.

f) Condition that precludes the safe performance of routine lumbar puncture, such as

prohibitive lumbar spinal disease, bleeding diathesis, or clinically significant

coagulopathy or thrombocytopenia.

g) Any other medical or psychiatric condition or lab abnormality, which in the opinion of

the investigator might preclude participation.

7

.2 Parkinson Disease (PD)

Note: Active PD participants previously enrolled in PPMI do not require re-assessment of

eligibility criteria listed below for enrollment in PPMI Clinical. Active participants do need

to be able to provide informed consent for PPMI Clinical participation (includes use of a

designated research proxy).

7

.2.1 Inclusion Criteria (PD)

a) Male or female age 30 years or older at Screening Visit.

b) A diagnosis of Parkinson disease for 2 years or less at Screening Visit.

c) Not expected to require PD medication within at least 6 months from Baseline.

d) Patients must have at least two of the following: resting tremor, bradykinesia,

rigidity (must have either resting tremor or bradykinesia); OR either asymmetric

resting tremor or asymmetric bradykinesia.

e) Hoehn and Yahr stage I or II at Baseline.

f) Individuals taking any of the following drugs: alpha methyldopa, methylphenidate,

amphetamine derivatives or modafinil, must be willing and medically able to hold

the medication for at least 5 half-lives before DaTscan imaging.

g) Confirmation that participant is eligible based on Screening DaTscan imaging.

h) Able to provide informed consent.

i) Either is male, or is female and meets additional criteria below, as applicable:

•

Female of childbearing potential who is not pregnant, lactating, or planning

pregnancy during the study and has a negative pregnancy test on day of

Screening DaTscan imaging test prior to injection of DaTscan^TM^.

7

.2.2 Exclusion Criteria (PD)

a) Currently taking levodopa, dopamine agonists, MAO-B inhibitors (e.g., selegiline,

rasagiline), amantadine or another PD medication.

b) Has taken levodopa, dopamine agonists, MAO-B inhibitors or amantadine within

6

0 days of Baseline visit.

PPMI

Version Date: 01Feb2021

Page 14 of 55


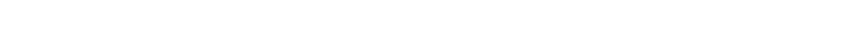


DocuSign Envelope ID: 706F2DD8-8346-4731-A419-FF8E7C48DF97

c) Has taken levodopa or dopamine agonists prior to Baseline visit for more than a

total of 90 days.

d) Atypical PD syndromes due to either drugs (e.g., metoclopramide, flunarizine,

neuroleptics) or metabolic disorders (e.g., Wilson’s disease), encephalitis, or

degenerative diseases (e.g., progressive supranuclear palsy).

e) A clinical diagnosis of dementia as determined by the investigator.

f) Previously obtained MRI scan with evidence of clinically significant neurological

disorder (in the opinion of the Investigator).

g) Received any of the following drugs: dopamine receptor blockers (neuroleptics),

metoclopramide and reserpine within 6 months of Screening visit.

h) Current treatment with anticoagulants (e.g., coumadin, heparin, oral thrombin

inhibitors) that might preclude safe completion of the lumbar puncture.

i) Condition that precludes the safe performance of routine lumbar puncture, such as

prohibitive lumbar spinal disease, bleeding diathesis, or clinically significant

coagulopathy or thrombocytopenia.

j) Any other medical or psychiatric condition or lab abnormality, which in the opinion

of the investigator might preclude participation.

7

.3 Parkinson Disease (PD) with LRRK2 or GBA mutation

Note: Active PD participants previously enrolled in PPMI do not require re-assessment of

eligibility criteria listed below for enrollment in PPMI Clinical. Active participants do need

to be able to provide informed consent for PPMI Clinical participation (includes use of a

designated research proxy).

7

.3.1 Inclusion Criteria (PD - LRRK2 or GBA)

a) Male or female age 30 years or older at Screening Visit.

b) A diagnosis of Parkinson disease for 2 years or less at Screening Visit.

c) Patients must have at least two of the following: resting tremor, bradykinesia,

rigidity (must have either resting tremor or bradykinesia); OR either asymmetric

resting tremor or asymmetric bradykinesia.

d) Hoehn and Yahr stage I or II at Baseline.

e) Confirmation of causative LRRK2 or GBA (willingness to undergo genetic testing

as part of genetic screening and be informed of genetic testing results, or approved

documentation of prior genetic testing results).

f) Individuals taking any of the following drugs: alpha methyldopa, methylphenidate,

amphetamine derivatives or modafinil, must be willing and medically able to hold

the medication for at least 5 half-lives before DaTscan imaging.

g) Confirmation that participant is eligible based on Screening DaTscan imaging.

h) Able to provide informed consent.

i) Either is male, or is female and meets additional criteria below, as applicable:

•

Female of childbearing potential who is not pregnant, lactating, or planning

pregnancy during the study and has a negative pregnancy test on day of

Screening DaTscan imaging test prior to injection of DaTscan^TM^.

PPMI

Version Date: 01Feb2021

Page 15 of 55


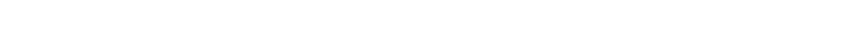


DocuSign Envelope ID: 706F2DD8-8346-4731-A419-FF8E7C48DF97

7

.3.2 Exclusion Criteria (PD - LRRK2 or GBA)

a) Received any of the following drugs: dopamine receptor blockers (neuroleptics),

metoclopramide and reserpine within 6 months of Screening visit.

b) Current treatment with anticoagulants (e.g., coumadin, heparin) that might preclude

safe completion of the lumbar puncture.

c) Condition that precludes the safe performance of routine lumbar puncture, such as

prohibitive lumbar spinal disease, bleeding diathesis, or clinically significant

coagulopathy or thrombocytopenia.

d) Any other medical or psychiatric condition or lab abnormality, which in the opinion

of the investigator might preclude participation.

7

7

.4 Parkinson Disease (PD) with SNCA or rare genetic variant

Note: Active PD participants previously enrolled in PPMI do not require re-assessment of

eligibility criteria listed below for enrollment in PPMI clinical. Active participants do need

to be able to provide informed consent for PPMI Clinical participation (includes use of a

designated research proxy).

.4.1 Inclusion Criteria (PD - SNCA or rare genetic variant (such as Parkin or Pink1))

a) Male or female age 30 years or older at Screening Visit.

b) Parkinson disease diagnosis at Screening Visit.

c) Patients must have at least two of the following: resting tremor, bradykinesia, rigidity

(must have either resting tremor or bradykinesia); OR either asymmetric resting tremor

or asymmetric bradykinesia.

d) Hoehn and Yahr stage I, II, or III at Baseline.

e) Confirmation of causative SNCA or rare genetic variant (such as Parkin or Pink1)

(willingness to undergo genetic testing as part of genetic screening and be informed of

genetic testing results, or approved documentation of prior genetic testing results).

f) Individuals taking any of the following drugs: alpha methyldopa, methylphenidate,

amphetamine derivatives or modafinil, must be willing and medically able to hold the

medication for at least 5 half-lives before DaTscan imaging.

g) Confirmation that participant is eligible based on Screening DaTscan imaging.

h) Able to provide informed consent.

i) Either is male, or is female and meets additional criteria below, as applicable:

•

Female of childbearing potential who is not pregnant, lactating, or planning

pregnancy during the study and has a negative pregnancy test on day of Screening

DaTscan imaging test prior to injection of DaTscan^TM^.

7

.4.2 Exclusion Criteria (PD - SNCA or rare genetic variant (such as Parkin or Pink1))

a) Received any of the following drugs: dopamine receptor blockers (neuroleptics),

metoclopramide and reserpine within 6 months of Screening visit.

b) Current treatment with anticoagulants (e.g., coumadin, heparin) that might preclude

safe completion of the lumbar puncture.

c) Condition that precludes the safe performance of routine lumbar puncture, such as

prohibitive lumbar spinal disease, bleeding diathesis, or clinically significant

coagulopathy or thrombocytopenia.

PPMI

Page 16 of 55

Version Date: 01Feb2021


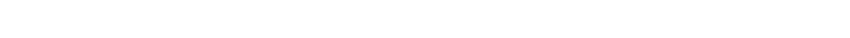


DocuSign Envelope ID: 706F2DD8-8346-4731-A419-FF8E7C48DF97

d) Any other medical or psychiatric condition or lab abnormality, which in the opinion of

the investigator might preclude participation.

7

.5 Prodromal

Note: Active Prodromal participants previously enrolled in PPMI do not require re-

assessment of eligibility criteria listed below for enrollment in PPMI Clinical. Active

participants do need to be able to provide informed consent for PPMI Clinical participation

(includes use of a designated research proxy).

The specific predictive eligibility criteria for participants recruited through PPMI Remote to

advance to PPMI Clinical will be iteratively optimized based on data collected from these

studies.

7

.5.1 Inclusion criteria (Prodromal)

For Screening:

a) Confirmation that participant is eligible based on centrally determined predictive

criteria including the University of Pennsylvania Smell Identification Test

(UPSIT).

•

For participants in PPMI Remote, referral to the clinical site confirms predictive

eligibility.

•

For participants identified by the clinical site, predictive criteria are based on

generalized risk such as first degree biologic relative, known risk of PD

including RBD, or known genetic variants associated with PD risk.

.

Additionally, confirmation of UPSIT eligibility during the Screening visit

prior to DaTscan.

b) Male or female age 60 years or older (except age 30 years or older for SNCA, or

rare genetic variants (such as Parkin or Pink1) participants).

c) Individuals taking any of the following drugs: alpha methyldopa, methylphenidate,

amphetamine derivatives or modafinil, must be willing and medically able to hold

the medication for at least 5 half-lives before DaTscan imaging.

d) Able to provide informed consent.

e) Either is male, or is female and meets additional criteria below, as applicable:

•

Female of childbearing potential who is not pregnant, lactating, or planning

pregnancy during the study and has a negative pregnancy test on day of

Screening DaTscan imaging test prior to injection of DaTscan^TM^.

For continuation to Baseline visit and ongoing follow-up:

f) Confirmation that participant is eligible based on *Screening DaTscan imaging.

*

Screening DaTscan imaging eligibility:

Based on the results of the DaTscan imaging test, Prodromal participants eligible to

continue their participation in PPMI Clinical will be asked to return for their PPMI

Clinical baseline visit. Neither the participant nor the site investigator will be made

aware of the participant’s DAT status during the study.

PPMI

Version Date: 01Feb2021

Page 17 of 55


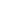

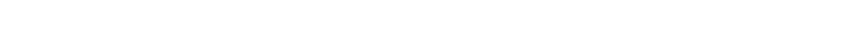


DocuSign Envelope ID: 706F2DD8-8346-4731-A419-FF8E7C48DF97

•

•

•

It is anticipated that approximately 6,000 participants will complete a screening

visit to undergo DAT imaging. Approximately 2,000 participants will be eligible

to continue their participation in PPMI Clinical (those not eligible to proceed will

remain in PPMI Remote, as applicable).

All participants with DAT deficit will be eligible to continue their participation in

PPMI Clinical. It is estimated that about 75% of eligible participants will have a

DAT deficit (defined by a hybrid of visual assessment and quantitative striatal

specific binding analysis).

Some participants without DAT deficit will also be eligible to continue their

participation in PPMI Clinical. These participants will be chosen based on DAT

binding that is reduced from age expected but it not outside the normal range and/or

from individuals with high-risk of PD including RBD, LRRK2, GBA, SNCA, or

rare genetic variants (such as Parkin or Pink1) that do not demonstrate DAT deficit.

It is estimated that about 25% of eligible participants will not have a DAT deficit.

It is anticipated that approximately 30% of the PPMI Clinical prodromal

participants with DAT deficit will phenoconvert to motor parkinsonism during a 3

to 5-year follow-up.

•

7

.5.2 Exclusion Criteria (Prodromal)

a) Clinical diagnosis of PD, other parkinsonism, or dementia.

b) Received any of the following drugs: dopamine receptor blockers (neuroleptics),

metoclopramide and reserpine within 6 months of Screening Visit.

c) Current treatment with anticoagulants (e.g. coumadin, heparin) that might preclude

safe completion of the lumbar puncture.

d) Condition that precludes the safe performance of routine lumbar puncture, such as

prohibitive lumbar spinal disease, bleeding diathesis, or clinically significant

coagulopathy or thrombocytopenia.

e) Any other medical or psychiatric condition or lab abnormality, which in the opinion

of the investigator might preclude participation.

8

OBTAINING INFORMED CONSENT

8

.1 Consent Process

The procedures and requirements of the study, together with any potential hazards/risks, and

the freedom to withdraw from participation in the study at any time, will be explained to each

potential participant as part of the consent process. The consent process will take place in a

space that allows for privacy and confidentiality and should allow for enough time for the

individual to consider participation and ask any questions. Consent will be obtained by the

study Investigator or delegated study staff, as applicable. When it is not possible for active

PPMI individuals transitioning into this protocol to have an in-person discussion of the PPMI

study (e.g., disease progression too advanced, health/safety reasons such as COVID-19), sites

may obtain informed consent remotely (e.g., by telephone or videoconference) after the

consent form has been provided to the potential participant (e.g., mail, email, e-sign

document). If the individual agrees to participation, the signed consent will be returned to

the site (e.g., mail, email, e-sign document) for signature by the person obtaining consent

before any research procedures begin. Each participant will sign such an informed consent

PPMI

Page 18 of 55

Version Date: 01Feb2021


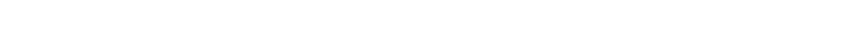


DocuSign Envelope ID: 706F2DD8-8346-4731-A419-FF8E7C48DF97

to document agreement to participate in the study, as well as to document HIPAA

authorization and compliance with GDPR regulation, as applicable. The signed informed

consent may be uploaded to a secure portal for remote monitoring.

It is the responsibility of the Investigator (or as delegated to the person obtaining consent) to

make sure that the participant understands what she/he is agreeing to and that informed

consent is obtained before the participant is involved in any protocol-defined procedures,

including screening procedures. Each participant will be provided a copy of the consent

form(s). There will be two consents for the PPMI Clinical prodromal cohort as they will

initially consent to the Screening visit and then, if eligible, will consent to the longitudinal

PPMI Clinical procedures. In addition to obtaining initial consent to participate,

Investigators must ensure ongoing consent as part of this longitudinal study (for example,

documentation at an annual study visit that the participant continues to understand the

procedures and requirements of the study).

8

.2 Identification of Research Proxy

There is the potential for development of cognitive impairment in participants over the course

of study participation. Therefore, in accordance with good clinical practices in ensuring each

participant’s ability to give ongoing informed consent, identification of a research proxy will

enable continued participation for participants whose ability to consent becomes

compromised. Identification of a research proxy through use of the Advance Directive for

Clinical Research Participation form, enables participants to clarify their preferences, thus

guiding the substitute decision maker and the Investigator. It is noted that the accepted term

and/or required directive for a designated substitute decision maker (also known as a Legally

Authorized Representative/LAR) may vary on a country/state/provincial basis.

During the initial consent process, or at any time during assessment of ongoing consent as

applicable, a participant may identify a substitute decision maker who will be permitted to

carry out the participant’s wishes regarding continued participation (or not) in PPMI Clinical

should the participant lose the ability to make his or her own decision. The site Investigator

will exercise clinical judgment and ascertain a participant’s ability to continue giving

informed consent. This ascertainment may include a discussion including review of the study

purpose, differences between research and clinical assessments, and the risks of study

participation. If deemed necessary by the Investigator, the participant will be approached

about contacting the person(s) named in the advance directive while the participant is still

capable of discussing the need to invoke the research proxy. Should the Investigator deem it

necessary to invoke the LAR, the designated individual will be contacted by telephone, if not

already present at the study visit, to discuss the next steps for determining the participant’s

continuing participation.

Designation of a LAR is voluntary; thus, identification of a substitute decision maker is not

required to participate in PPMI. However, if in the absence of a substitute decision maker

the Investigator deems a participant no longer able to provide ongoing consent, the

participant will be withdrawn from the study.

PPMI

Page 19 of 55

Version Date: 01Feb2021


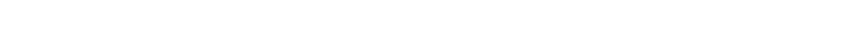


DocuSign Envelope ID: 706F2DD8-8346-4731-A419-FF8E7C48DF97

Documentation is required for completion of the Advance Directive, routine review of the

participant’s continuing ability to give informed consent at each visit, any discussion with

the participant’s substitute decision maker, as well as documentation of informed consent

(and assent of the participant) should a LAR be invoked.

8

.3 Permission to be Contacted for Follow Up of Persons with Neurologic Disease

The Follow Up of Persons with Neurologic Disease (FOUND) study (Caroline Tanner MD,

Principal Investigator, University of California-San Francisco (UCSF)) provides a parallel,

centralized system to prospectively collect vital status and disease progression information

from persons with parkinsonism, related disorders and healthy controls who are participating

in clinical research studies. Participation in FOUND complements in-person assessment,

enables continuity of follow up of individuals who complete or withdraw from a study, and

may also aid in PPMI study retention. Participation in FOUND will enable centralized

contact both during and after completion of PPMI, using convenient methods for systematic

data collection (e.g., regular mail, telephone, internet contacts).

During the initial consent process for PPMI Clinical, and as needed at subsequent follow up

visit, participants will be asked if their contact information may be shared with the FOUND

study team at UCSF. The participant’s decision will be documented in the PPMI informed

consent and the PPMI database. If a participant agrees, UCSF will be notified and will

proceed with contacting the individual to invite participation into FOUND. UCSF will share

with the referring sites their participants’ status in FOUND at regular intervals. PPMI

participants who have incomplete enrollment in FOUND will be asked by the site to discuss

this with the participant to identify if there are any issues impeding enrollment and address

any such issues. The data collected from the FOUND study will be uploaded into the PPMI

data repository at the Laboratory of Neuro Imaging (LONI), The Institute for Neuroimaging

and Informatics in Los Angeles, California, at regular intervals.

8

.4 Permission to be Contacted from Pathology Core

Post-mortem analysis of brain tissue is pivotal to Parkinson’s disease research, allowing

researchers to examine changes noted in the post-mortem brain tissue and correlate it with

changes in neuropsychological, imaging, and biomic data collected throughout the PPMI

Clinical study. However, there is limited availability to this type of tissue, leading to

organized efforts to facilitate brain donation planning through the PPMI Pathology Core.

The PPMI Pathology Core is a collaboration between Indiana University and Stanford

University. Indiana University is responsible for coordinating all logistics up-to death,

including obtaining consent, identifying a removal specialist, coordinating with clinical sites,

and interfacing with the decedent’s family. Indiana University also ensures the removal

specialist follows outlined removal and shipping guidelines to transfer the whole brain to the

Stanford team, while a small tissue sample is shipped to Indiana University for DNA

extraction. Stanford University is responsible for post-mortem activities including receiving

specimens, specimen dissection and preparation for embedding and processing, performing

neuropathological evaluation of tissue, coordinating clinicopathological case conferences

(CPCs), and long-term storage of brain tissue samples.

PPMI

Page 20 of 55

Version Date: 01Feb2021


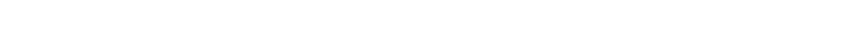


DocuSign Envelope ID: 706F2DD8-8346-4731-A419-FF8E7C48DF97

For clinical sites based in the United States, site coordinators will discuss the PPMI

Pathology Core with participants and provide them with an information at initial consent to

PPMI, or subsequent study visits as applicable. Participants will be asked to provide

permission to allow their contact information to be transferred to the Pathology Core team.

Participants may also contact the team at Indiana University directly to learn more about

enrollment. The Pathology Core team will contact participants to discuss tissue donation

further and answer questions. If participants are agreeable to continue with donation

planning, they will first be asked to sign a consent form that reflects their intent to donate

brain tissue and other relevant tissue upon death. This consent is approved by the Indiana

University IRB. After consent, the participant will provide additional information to help

with their local planning and coordination.

The Indiana and Stanford University teams will also provide support to international PPMI

sites that are interested in contributing to PPMI brain tissue donation activities. Stanford

University will work with neuropathologists at local sites to ensure the harmonization of

brain tissue collection and processing across all PPMI sites. Indiana University will help

establish workflows from consent to donation and ensure regulatory considerations are met

for participant inclusion in the PPMI Pathology Core.

The data collected across the Pathology Core will be collated by the team at Indiana

University and transferred to the PPMI data repository at the Laboratory for Neuro Imaging

(LONI), The Institute for Neuroimaging and Informatics in Los Angeles, California, at

regular intervals. It is possible that collected tissues may be distributed to approved

researchers for future analysis.

9

PARTICIPANT INFORMATION AND STUDY ID

9

9

.1 Participant Profile Information

When a participant provides consent to participate, the following required participant

identifiers will be collected in the electronic database capture (EDC) system: first name,

last name, email address, date of birth, and sex. In addition, the following (optional)

participant identifiers will be collected: middle name, city/municipality of birth and

country of birth.

.2 Participant ID Number

A Participant ID number will be assigned to all PPMI Clinical participants, if not

previously assigned under another PPMI program protocol. Active PPMI participants

transitioning into this PPMI protocol will keep their previously assigned PPMI ID

number, while newly enrolled participants will be assigned a new 6-digit ID number,

generated automatically by EDC. The PPMI Participant ID number will be used to

identify a participant on all study related documentation (e.g., clinical database,

biological specimens).

9

.3 Globally Unique Identifier (GUID) Number

Participant’s identifiers may be used to generate a GUID number. Use of this ID can

track an individual’s participation across multiple studies without storing any personally

PPMI

Version Date: 01Feb2021

Page 21 of 55


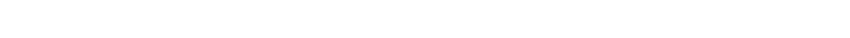


DocuSign Envelope ID: 706F2DD8-8346-4731-A419-FF8E7C48DF97

identifiable information. The protected system used to create the GUID uses an

algorithm of data element inputs, such as those collected in the participant’s profile (see

section 9.1 above) and produces an electronic “fingerprint” output. The system stores

only the “fingerprint” and clears the individual’s inputted data elements from memory.

The participant is then assigned a 9-digit Unique ID Number that is associated with their

electronic “fingerprint.”

1

0

STUDY VISIT PROCEDURES

Screening, Baseline and Annual study visits may occur over the period of more than one day

due to the complexity of the visits and resources required at the site. The date each

assessment was completed will be captured within the EDC system and will therefore reflect

whether a visit required a duration of more than one day to complete.

The Baseline visit should be completed within 60 days of the Screening visit. Follow up 6

month and annual visits should be completed with +45 days of the target visit date. Out of

window visits will not be considered a protocol deviation but will be monitored throughout

the study for each site.

Assessments that require completion by the Site Investigator (or trained designee)

include the following (it is the goal of the study that the clinical assessments be

conducted by the same individual throughout the study):

•

•

•

•

•

•

•

•

•

•

Informed Consent

Research proxy designation

Review Inclusion/Exclusion criteria

Neurological Examination

MDS-UPDRS Parts Ia, III, IV, MDS-UPDRS Repeat Part III, Hoehn & Yahr

Modified Schwab & England ADL

Features of Parkinsonism

Other Clinical Features

Primary Diagnosis

Cognitive Categorization

1

0.1 Active PPMI Transitioning Participants

Refer to the PPMI Schedule of Activities for the applicable cohort to determine the

activities to be conducted at each visit. Participants will continue from their original

PPMI study schedule into the next planned study visit under this protocol. Note that

additional “Transition Activities” must be completed as outlined in the Schedule of

Activities for all participants transitioning into this protocol at their first in-person

visit.

Active participants previously enrolled in PPMI will not require a Screening or Baseline

visit. Participants who agree to continue participation and transition into PPMI Clinical will

enroll into PPMI and complete the next planned study visit based on the last completed visit

in PPMI (or based on timing of site activation and participant’s visit schedule). The process

of obtaining informed consent, including an explanation of study activities, is described in

the cohort visits below and will be conducted prior to completing any PPMI Clinical study

PPMI

Page 22 of 55

Version Date: 01Feb2021


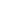

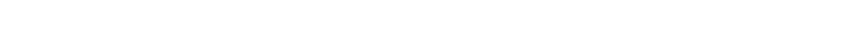


DocuSign Envelope ID: 706F2DD8-8346-4731-A419-FF8E7C48DF97

activities. Participants transitioned into PPMI Clinical will be followed for a minimum of 5

years, either in person or remotely according to the respective cohort’s schedule of activities.

Active PPMI participants choosing not to continue into PPMI Clinical protocol will be

tagged by the site as “Complete”.

1

0.2 Healthy Control, PD and PD Genetic Cohort Visits

1

0.2.1 HC, PD and PD Genetic Screening Visit

Refer to the PPMI Schedule of Activities for the applicable cohort to determine

the activities to be conducted at the Screening visit.

All newly enrolled participants in these cohorts will undergo a screening evaluation prior

to the Baseline visit. The Screening visit will take about 8 hours to complete (could occur

over more than one day).

During the informed consent process the following activities will also be described, as

applicable:

•

•

•

Discuss either start of participation or continued participation in PPMI Digital

Application study.

Discuss PPMI Online study expectation to provide ongoing response to participant

questionnaires.

An explanation of FOUND in PPMI will be given and participants will be asked

permission to have their contact information sent to the FOUND coordinating site at

UCSF so that UCSF study team can contact them about their interest in participation.

Participants may take time to review and complete at a subsequent visit.

An explanation of the PPMI Pathology Core will be given and participants will be

asked permission to have their contact information sent to the Pathology Core study

team. Participants may take time to review and complete at a subsequent visit.

An explanation of the purpose and procedures for identification of a substitute

decision maker (or research proxy) will be given. Participants may take time to

review and complete at a subsequent visit.

•

•

•

The investigator will review participants’ continuing ability to give informed consent

at each in-person visit and document this within the EDC system.

1

0.2.2 HC, PD and PD Genetic Baseline Visit

Refer to the PPMI Schedule of Activities for the applicable cohort to determine the

activities to be conducted at the Baseline visit.

Once all study procedures are completed, the Investigator must ensure that the participant

meets eligibility for the relevant cohort in order to continue with longitudinal follow up

visits. This Baseline visit is anticipated to take 8 hours (could occur over more than one

day).

1

0.2.3 HC, PD and PD Genetic Follow up Visits

Refer to the PPMI Schedule of Activities for the applicable cohort to determine the

activities to be conducted at follow up visits.

PPMI

Page 23 of 55

Version Date: 01Feb2021


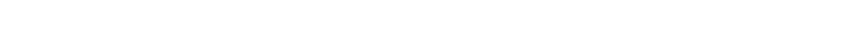


DocuSign Envelope ID: 706F2DD8-8346-4731-A419-FF8E7C48DF97

After the Baseline visit is completed, participants will be evaluated in clinic every 6

months for the first two years. Annual visits are anticipated to take about 6-8 hours

(could occur over more than one day), while the 6-month in clinic visits will take about

2

-4 hours. After two years, all participants will continue to be evaluated every 6 months

remotely and annually in the clinic, for a minimum of 5 years of longitudinal follow up

visits. Options for Remote 6-month visits include virtual visits by video link or

telemedicine, or phone/audio only. The remote 6-month visits will take about 1-2 hours.

Sites should complete as many assessments at the Remote (“R”) visit as is feasible, based

on how the visit is conducted (i.e., videoconference versus in-home or audio only) and

ability to do an assessment.

1

0.3 Prodromal Cohort Visits

1

0.3.1 Prodromal Screening Visit

Refer to the PPMI Schedule of Activities for the Prodromal cohort to determine the

activities to be conducted at the Screening visit.

Participants eligible for PPMI Clinical Prodromal cohort will undergo a screening

evaluation, which may include completion of a UPSIT if recruited directly by the site, as

well as DaTscan imaging for all participants prior to the Baseline visit. Informed consent

for the Prodromal Screening visit will be obtained by the site and eligibility to complete

the DaTscan imaging confirmed. Sites must have confirmation that a participant is

eligible based on the DaTscan prior to proceeding to the Baseline visit, at which time the

participant will be fully consented to the PPMI Clinical protocol. The Screening Visit

will take about 6-8 hours to complete (could occur over more than one day).

1

0.3.2 Prodromal Baseline Visit

Refer to the PPMI Schedule of Activities for the Prodromal cohort to determine the

activities to be conducted at the Baseline visit.

Prodromal cohort participants eligible to proceed to the Baseline visit will first consent

to the full PPMI Clinical study. During the informed consent process the following

activities will also be described, as applicable:

•

•

•

Discuss either start of participation or continued participation in PPMI Digital App

study.

Discuss continued participation in the PPMI Online study with expectation to provide

ongoing response to participant questionnaires.

An explanation of FOUND in PPMI will be given and participants will be asked

permission to have their contact information sent to the FOUND coordinating site at

UCSF so that UCSF study team can contact them about their interest in participation.

Participants may take time to review and complete at a subsequent visit.

An explanation of the PPMI Pathology Core will be given and participants will be

asked permission to have their contact information sent to the Pathology Core study

team. Participants may take time to review and complete at a subsequent visit.

•

PPMI

Version Date: 01Feb2021

Page 24 of 55


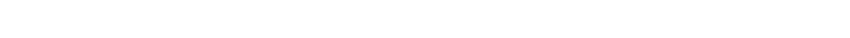


DocuSign Envelope ID: 706F2DD8-8346-4731-A419-FF8E7C48DF97

•

•

An explanation of the purpose and procedures for identification of a substitute

decision maker (or research proxy) will be given. Participants may take time to

review and complete at a subsequent visit.

The investigator will review participants’ continuing ability to give informed consent

at each in-person visit and document this within the EDC system.

Once all Baseline study procedures are completed, the Investigator must ensure that the

participant meets eligibility in order to continue with longitudinal follow up visits. This

Baseline visit is anticipated to take about 6-8 hours (could occur over more than one day).

1

0.3.3 Prodromal Follow Up Visits

Refer to the PPMI Schedule of Activities for the Prodromal cohort to determine the

activities to be conducted at the follow up visits.

After the Baseline visit is completed, participants will be evaluated in clinic annually and

remotely every 6 months, for a minimum of 5 years of longitudinal follow up visits.

Annual visits are anticipated to take about 6-8 hours (could occur over more than one

day). Options for Remote 6-month visits include virtual visits by video link or

telemedicine, or phone/audio only. The remote 6-month visits will take about 1-2 hours.

Sites should complete as many assessments at the Remote (“R”) visit as is feasible, based

on how the visit is conducted (i.e., videoconference versus in-home or audio only) and

ability to do an assessment.

1

0.3.4 Prodromal – Diagnostic Visit

If a Prodromal participant is clinically diagnosed with PD or other neurodegenerative

disorder during a scheduled visit, study procedures will follow the applicable standard

visit schedule of activities and the visit will be labeled as a Standard visit.

If a Prodromal participant is suspected to have developed PD or is diagnosed with PD or

other neurodegenerative disorder outside the window of a scheduled visit, then the

participant should be contacted to return for an in-person assessment called a diagnostic

visit. This in-person visit should occur within 45 days of the site being aware of the

diagnosis. The Visit Status data form should indicate the type of visit is a “Diagnostic

visit” and should be conducted as outlined below.

1

) Investigator determines participant has diagnosis of PD or other

neurodegenerative disorder.

If at the in-person diagnostic visit the investigator determines that the

participant has a diagnosis of PD or another neurodegenerative disorder, then:

a) If the next planned visit is an annual study visit, this diagnostic visit will

take the place of the next annual visit. The participant will undergo all

assessments scheduled for that annual visit.

•

If not already scheduled as part of that visit, DaTscan imaging

and MRI will be added.

PPMI

Page 25 of 55

Version Date: 01Feb2021


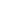

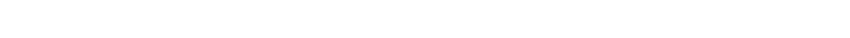


DocuSign Envelope ID: 706F2DD8-8346-4731-A419-FF8E7C48DF97

b) If the next planned visit is a remote study visit, then that remote visit

will be replaced by this in person diagnostic visit. The following

assessments will be added to those already scheduled as part of the

remote visit:

•

Blood and urine research sample collection.

2

) Investigator determines participant does not have diagnosis of PD or other

neurodegenerative disorder.

If at the in-person diagnostic visit the investigator determines that the

participant does not have a diagnosis of PD or another neurodegenerative

disorder, then no further study assessments should be conducted. This visit

will not take the place of an annual or remote visit, rather will be labeled as

an Unscheduled visit. Ensure the Visit Status data form indicates the type

of visit is a “Diagnostic visit”. Resume the participant’s regular visit

schedule.

1

0.4 Need for PD Therapy Visit

The need for PD therapy may be identified in the following ways:

1

2

3

) Participants may identify the need for PD therapy and start treatment outside of a

study visit.

) Participants may identify or confirm the need for PD therapy and start treatment at a

study visit.

) Participants may identify the need for PD therapy at a study visit, but start treatment

following the study visit.

If a PD participant or a prodromal participant identify the need for PD medication at an in-

person visit, the visit will follow the schedule of activities for that visit. Ensure the Visit

Status data form indicates the type of visit is a “Need for PD Therapy visit”. Resume the

participant’s regular visit schedule following that visit.

If the site becomes aware that a participant will begin PD medication outside of a scheduled

in person visit, the site should determine if the participant is willing to return to conduct an

in person visit prior to starting PD medications. If a participant is unwilling or unable to

return for an in person visit prior to starting PD medication, conduct the next study visit per

the regular visit schedule (participant should arrive at visit holding medication). If the

participant agrees, schedule an in-person visit as soon as possible following the participant’s

decision to request medication. The visit for starting PD medication assessments will follow

the schedule of activities of the next planned in-person visit. Ensure the Visit Status data

form indicates the type of visit is a “Need for PD Therapy visit”. Resume the participant’s

regular visit schedule following that visit.

If a participant begins PD medications following the completed need for therapy visit, the

site will communicate by phone with the participant to obtain and record the medication dose

and start date.

PPMI

Page 26 of 55

Version Date: 01Feb2021


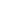

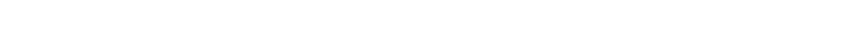


DocuSign Envelope ID: 706F2DD8-8346-4731-A419-FF8E7C48DF97

1

0.5 Premature Withdrawal Visit

If a participant withdraws from the study during a scheduled annual visit, proceed with the

visit as outlined. Ensure the Visit Status data form indicates the type of visit is a “Premature

Withdrawal visit”.

If a participant withdraws from the study outside of a scheduled annual visit and agrees to be

seen for one more visit, the next scheduled annual visit should be completed. Study

procedures will be the same as outlined in the schedule of activities for that respective annual

visit, except for the activities as outlined below. Alternatively, if the participant is unwilling

or unable to return for a final in person visit, the premature withdrawal visit could be

completed by video link or telemedicine. In addition to completing the activities outlined in

the schedule of activities, the site will complete the Conclusion of Participation assessment

and indicate the reason for study withdrawal. Ensure the Visit Status data form indicates the

type of visit is a “Premature Withdrawal visit”.

Premature Withdrawal Visit – Activity Exceptions:

•

•

•

•

•

Blood and urine research sample collection – only if not done in the last 3 months

Lumbar puncture for collection of CSF – only if not done in the last 3 months

Skin biopsy – only if not done in the last 6 months

MRI – only if not done in the last 6 months

DaTscan Imaging – only if not done in the last 6 months

1

0.6 Unscheduled Visits

An unscheduled visit may be performed only if required (i.e., deemed necessary to follow up

on adverse events, or deemed clinically relevant by the site Investigator to ensure the safety

of the participant). The following activities will be completed:

•

•

•

•

•

•

Vital signs

*Neurological examination

*Blood sample for clinical laboratory assessments

Review of current medical conditions

Review of concomitant medications

Review of adverse events

*

Conducted only if clinically indicated

1

0.7 Out of Clinic Annual Visits

To enable continued involvement of participants in the PPMI Clinical study and enhance

study retention, participants who are unable to attend annual visits in person due to reasons

such as participant burden, advanced disease, and/or participant safety (e.g., such as COVID-

1

9), may be eligible for assessment out of the clinic. Permission to have an out of clinic visit

must be obtained in advance from the PPMI Executive Steering Committee, or designee,

who will determine participant’s eligibility to participate via remotely completed

assessments. This permission may be applied to the study more broadly or PPMI sites will

notify the Site Management Core about a participant’s inability to present for an in-person

clinic visit. The site will be informed of any decision made regarding Out of Clinic visits and

will determine the appropriate approach for conducting this visit. Options for Out of Clinic

visits include virtual visits by video link (i.e., telemedicine), enhanced telephone,

PPMI

Page 27 of 55

Version Date: 01Feb2021


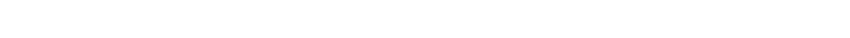


DocuSign Envelope ID: 706F2DD8-8346-4731-A419-FF8E7C48DF97

phone/audio only, or in-home assessments in which PPMI site staff travel to the participant’s

home. Sites should complete as many assessments indicated for the scheduled annual visit

as is feasible, based on the type of out of clinic visit conducted and ability to do an assessment

(i.e., videoconference versus in-home or audio only). Ensure the Visit Status data form

indicates the type of visit is an “Out of Clinic visit”.

1

1

2

CLINICAL ASSESSMENTS

Refer to the PPMI Assessments and eCRF Completion Manual for a detailed

description of the clinical assessments and instructions for administration.

1

SAFETY ASSESSMENTS

1

2.1 Medical Conditions Review, Physical and Neurological Examination

Medical and family history, as well as a complete physical and neurological exam will be

captured on all participants according to the schedule of activities. A neurological exam will

also be conducted annually, as well as at the last completed visit if possible.

1

2.2 Vital Signs/Weight/Height

Pulse rate (supine and standing), blood pressure (supine and standing), and oral temperature

will be determined at every visit. The supine blood pressure and pulse rate will be determined

after 1-3 minutes of quiet rest and the standing pressure and rate will be determined after 1-

3

minutes in the standing position. Weight and height will also be collected at baseline and

annually.

1

2.3 Clinical Laboratory Tests

Routine clinical safety laboratory tests indicated in the table below will be performed at the

first visit only for new enrollments (i.e., Screening for PD and Healthy Controls, Baseline

for Prodromal participants). A central laboratory will be implemented in order to conduct

identical analysis methods and utilize consistent normal ranges and thus common

interpretation of laboratory changes. If not stated otherwise, venous whole blood will be

collected in blood collection tubes (vacutainers). All samples for laboratory analysis must

be collected, prepared, labelled, and shipped according to the laboratory’s requirement as

detailed in the lab manual. The total amount of blood needed for the clinical safety lab tests

will be no more than 5 ml. No more than 60 ml will be drawn at either the Screening or

Baseline visit, including both safety and research blood samples.

The coagulation panel (PT/PTT) will be collected and shipped by all sites to the central lab

for analysis for the first visit only for new enrollments (i.e., Screening for PD and Healthy

Controls, Baseline for Prodromal participants). Sites have the option, per clinical practice,

to collect an additional blood sample to evaluate coagulation results prior to the conduct of

post Baseline Visit lumbar puncture assessments. The sample should be sent to a local lab

facility for analysis. Results will be evaluated to determine, in the opinion of the Investigator,

whether there are any issues that may preclude conduct of the follow up lumbar puncture.

Results should be maintained as part of the participant’s study documents; however, will not

be included in the study database.

PPMI

Page 28 of 55

Version Date: 01Feb2021


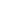

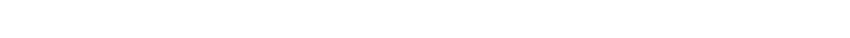


DocuSign Envelope ID: 706F2DD8-8346-4731-A419-FF8E7C48DF97

CENTRAL LAB TESTS

METABOLIC PANEL COMPLETE BLOOD COUNT

Sodium (Na)

Potassium (K)

Chloride (Cl)

White Blood Cell Count (WBC)

Red Blood Cell Count (RBC)

Hemoglobin (Hb)

Carbon Dioxide (CO2)

Blood Urea Nitrogen (BUN)

Glucose

Hematocrit (HCT)

Platelet Count (PLT)

Calcium (Ca)

Creatinine (Crn)

Bilirubin Total

Albumin

Total Protein

Aspartate aminotransferase (AST)

Alanine aminotransferase (ALT)

Alkaline Phosphatase (ALKP)

Uric Acid

Prothrombin time (PT) – Screening Only

Partial Thromboplastin Time (PTT) – Screening Only

1

3

BIOLOGIC RESEARCH SAMPLING

Refer to the PPMI Biologics Manual for the detailed description of the biologic samples

collected and processing instructions.

1

3.1 Blood Samples

Whole blood (about 10 ml), serum (about 30 ml) and plasma (about 10 ml) will be collected

to conduct proteomic, metabolomic, genetic and other research analyses. No more than 60

ml will be drawn at any visit, including both clinical safety labs and research blood samples.

It is strongly advised that the research blood samples are collected in a fasted state (i.e.,

minimum of 8 hours since last meal/food intake) to ensure the quality of samples for

future analyses. If fasting is not possible, then participants should be advised to eat a low

lipid diet. All research samples will be sent to a central biorepository to be stored indefinitely

for research purposes. Samples will be made available to researchers to conduct analyses

related to PD and other disorders. Participants will not receive any individual results of

research analysis or testing conducted on the biologic samples.

1

3.2 Urine

Urine (about 10 ml) will be collected to conduct analyte analyses.

1

3.3 Lumbar Puncture / Cerebral Spinal Fluid (CSF)

The lumbar puncture (LP) is performed by the site investigator or another qualified clinician

appointed by the investigator. A lumbar puncture for the collection of 15-20 ml of CSF will

be conducted for all participants per the visit schedule unless there is evidence of clinically

significant coagulopathy or thrombocytopenia that would interfere with the safe conduct of

the procedure. The first 2 ml of CSF will be processed for cell count, protein, and glucose

levels. Participants will be closely monitored the day of the procedure for adverse events.

PPMI

Page 29 of 55

Version Date: 01Feb2021


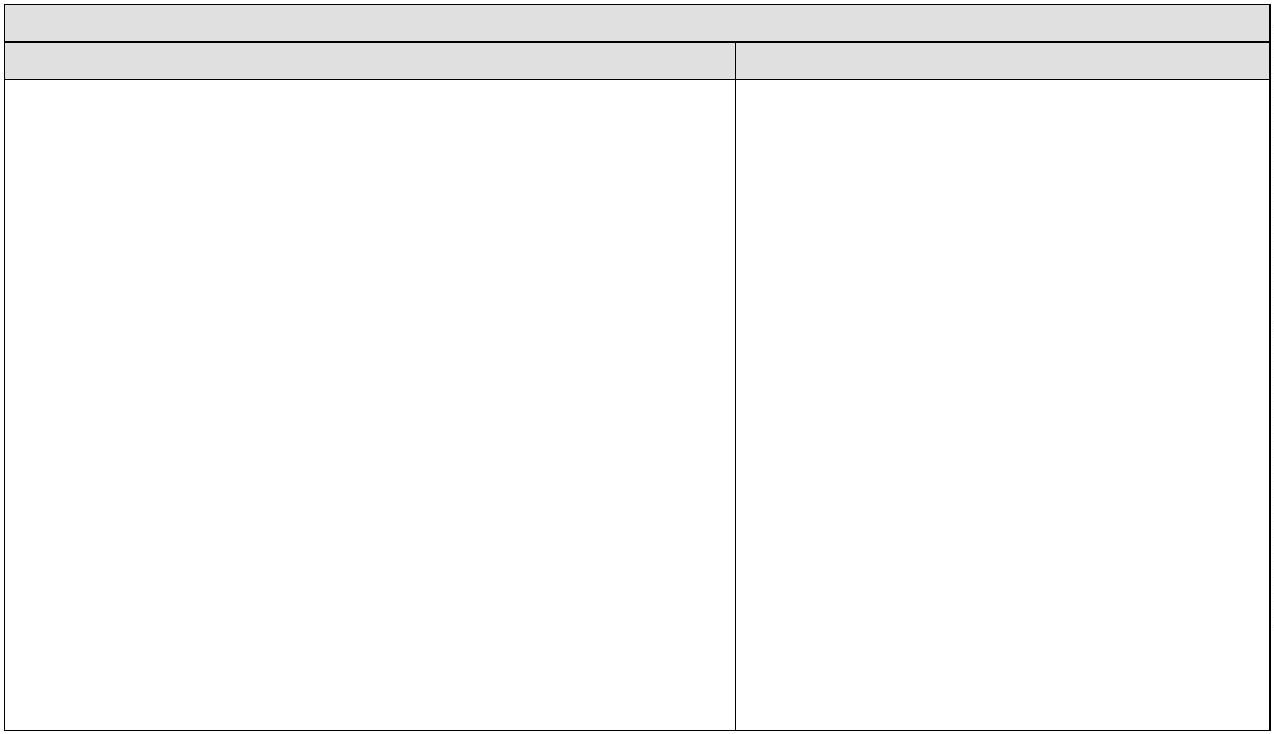

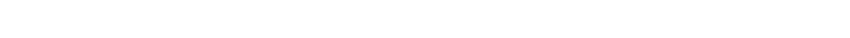


DocuSign Envelope ID: 706F2DD8-8346-4731-A419-FF8E7C48DF97

Participants will be also be contacted by phone 2 to 3 [business/working] days following an

LP to assess for any adverse events. The CSF samples will be sent to a central biorepository

to be stored indefinitely for research purposes. The CSF samples will be made available to

researchers to conduct analyses related to PD and other disorders.

1

3.4 Skin Biopsy

The skin biopsy is performed by the site investigator or another qualified clinician appointed

by the investigator. Skin punch biopsy will be performed under local anesthesia (lidocaine)

in the posterior neck according to the Schedule of Activities. Up to two punches will be

completed and the skin samples will be processed as described in the PPMI Biologics Manual

and shipped to the central biorepository for storage and analysis. Remaining samples may be

used to evaluate other proteins, analytes or potential biomarkers. Participants will not receive

any individual results of analysis or testing conducted on the skin samples. Participants will

be monitored the day of the procedure for adverse events. Participants will be also be

contacted by phone 2 to 3 [business/working] days following a skin biopsy to assess for any

adverse events.

1

4

IMAGING

1

4.1 Dopamine Transporter SPECT Imaging

Refer to the PPMI SPECT Technical Operations manual for a detailed description of

the SPECT imaging procedures.

Participants will undergo dopamine transporter imaging to measure dopamine transporter

binding using single photon emission computed tomography (SPECT). All new participants

will undergo DaTscan imaging at Screening. All new and transitioning participants will also

undergo follow up DaTscan imaging as indicated in their cohort visit schedule.

To lessen participant burden, a participant’s previously acquired DaTscan may be used in

place of a newly acquired scan as long as the previous scan was acquired within 6 months

of the study scheduled DaTscan, it meets protocol acquisition standards, and passes QC

requirements for the research study analysis.

The DaTscan imaging procedure will be performed at the individual sites using DaTscan^TM^

to target the dopamine transporter and all imaging data will be submitted for analysis to the

Imaging core. DaTscan imaging eligibility will be determined using pre-specified imaging

cut-offs. DaTscan eligibility result will be made available to the participant’s clinical site.

Women of childbearing potential must have a urine (or serum if required by the site)

pregnancy test prior to injection of DaTscan^TM^. The result must be confirmed as negative

prior to proceeding with the injection. Before the DaTscan^TM^ injection, participants will be

pre-treated with stable iodine (10 drops of a saturated solution of potassium iodide) to reduce

the uptake of DaTscan^TM^ by the thyroid. If the participant is allergic to iodine, then potassium

perchlorate 400 mg) can be substituted for potassium iodide. Participants will be injected

with up to 5 mCi of DaTscan^TM^. Within a 4-hour (+/- 30 minute) window following the

PPMI

Page 30 of 55

Version Date: 01Feb2021


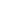

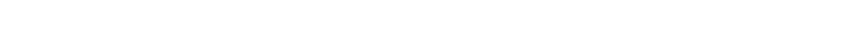


DocuSign Envelope ID: 706F2DD8-8346-4731-A419-FF8E7C48DF97

injection, participants will undergo DaTscan imaging for approximately 30 minutes (or up

to an hour if the participant moved during scanning).

Participants will be monitored by study personnel for adverse events on the day that a

dopamine transporter SPECT scan is obtained. Participants will also be contacted by phone

2

to 3 [business/working] days following the injection/scan to assess adverse events.

The product used to complete the dopamine transporter SPECT scans is being used “off-

label” in the PPMI Clinical study. The imaging result obtained from the scan is not intended

to provide information about a clinical diagnosis and will not be shared with participants.

1

4.2 Magnetic Resonance Imaging (MRI)

Refer to the PPMI MRI Technical Operations manual for a detailed description of the

MRI imaging procedures.

Participants will undergo an MRI brain scan at the Baseline visit and will also undergo

follow up MRI scans as indicated in the visit schedule. At the discretion of the Investigator

and Imaging staff, participants who have presence of pacemakers, aneurysm clips, artificial

heart valves, ear implants, metal fragments or foreign objects in the eyes, skin or body or

any other known contra-indication to MRI may be advised not to complete a Baseline (or

follow-up) MRI scan, but these participants may still participate in the study.

1

5

RISKS TO PARTICIPANTS

1

5.1 Blood Sampling

Risks associated with venous blood draw include pain and bruising at the site where the

blood is taken. Sometimes people can feel lightheaded or even faint after having blood

drawn.

1

5.2 MRI

Participants should notify the study doctor if they suffer from claustrophobia because they

may become anxious while in the magnetic resonance scanner. The investigator may treat

the participant for anxiety if indicated. There may be loud noises such as knocking or

hammering that occur while the MRI is being conducted. Participants should also inform

the study doctor if they have a pacemaker or metal implants (screws, plates or clips) because

this may preclude MR evaluation.

1

5.3 DaTscan Imaging

Risks of DaTscan^TM^: DaTscan^TM^ is administered at radiotracer doses and is not expected to

have any pharmacological or toxicological effects. DaTscan^TM^ binds to the dopamine and

serotonin transporter. At pharmacologic doses DaTscan^TM^ might be expected to have

stimulant-like effects and affect cardiovascular responses. However, in the proposed study

the estimated mass dose of DaTscan^TM^ is very low (<30/pmol kg). More than 500,000 doses

of the radiotracer have been administered to human participants.

PPMI

Page 31 of 55

Version Date: 01Feb2021


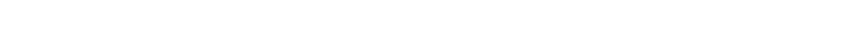


DocuSign Envelope ID: 706F2DD8-8346-4731-A419-FF8E7C48DF97

Iodine: Prior to each injection participants will be pretreated with Lugol’s (or similar)

solution, 10 drops of a saturated solution of potassium iodide) to reduce thyroid uptake of

the radioactive agent. Participants may experience a metallic or bitter taste in their mouths

from the iodine. Participants with allergies to iodine might get itching, a rash, bloating,

severe blood pressure changes (shock), and death if given iodine. Participants who are

allergic to iodine may be imaged without Lugol’s or if available may be administered

potassium perchlorate rather than Lugol’s.

In addition to the known risks listed above, these imaging procedures may cause unknown

risks to the participant, or a developing embryo or fetus or possible risks to the future

offspring of male participants. Female participants of childbearing potential will be asked

to have a pregnancy test. Female participants and male participants whose partners become

pregnant within 30 days of DaTscan^TM^ injection should report the pregnancy on the Report

of Pregnancy data form in EDC within 24 hours of notification of the pregnancy.

1

5.4 Lumbar Puncture

The most common risks of a lumbar puncture are pain at the site and a temporary headache

usually due to a small amount of CSF leakage around the needle insertion site. Lying down

for 30 -60 minutes after the test may make a headache less likely to occur. There is a slight

risk of infection because the needle breaks the skin’s surface, providing a possible portal of

entry for bacteria. A temporary numbness to the legs or lower back pain may be experienced.

There is a small risk of bleeding in the spinal canal. Participants will have blood drawn at

the Screening visit to test for coagulopathies.

1

5.5 Skin Biopsy

Risks associated with performing punch biopsies of the skin include pain and bruising at the

site where the biopsy is taken. There is a small risk that the biopsy site may change color.

The skin biopsy may leave a scar. There is also a small possibility of infection or bleeding

at the biopsy site. Although very rare, it is possible to have an allergic reaction to the local

anesthetic (lidocaine) or betadine.

1

5.6 Disclosure of Genetic Information

All genetic information will be maintained in a confidential research file. While every effort

will be made to maintain confidentiality there is a small risk that information will be

disclosed.

1

6

REFERRALS IN THE CASE OF CLINICALLY RELEVANT FINDINGS

If a research assessment, lab, or MRI reveals a clinically significant abnormality (e.g., MRI

structural lesion, indication of suicidality, depression, or renal impairment on metabolic

profile), the participant will be informed of this result and instructed to follow up with his or

her primary care physician. Should there be a safety concern warranting a referral for

medical or psychiatric follow-up, the Investigator should provide the participant with the

appropriate referral as necessary. The sites will follow their standard procedures for urgent

and non-urgent medical situations identified during study visits.

PPMI

Version Date: 01Feb2021

Page 32 of 55


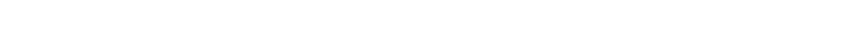


DocuSign Envelope ID: 706F2DD8-8346-4731-A419-FF8E7C48DF97

1

7

8

POTENTIAL BENEFITS TO PARTICIPANTS

There are no direct anticipated benefits to study participants in this study. However, new

information may be generated by the study that will support development of better treatments

for Parkinson’s disease.

1

CONCOMITANT MEDICATIONS

1

8.1 Use of Concomitant Medications

Concomitant medications, including over-the-counter (OTC), dietary supplements (e.g.,

herbal remedies) or prescriptions, are permitted during the study period, except for the

following medications that might interfere with dopamine transporter SPECT imaging

which are restricted for 5 half-lives prior to a DaTscan^TM^ injection: alpha methyldopa,

methylphenidate, modafinil, amphetamine derivatives and other CNS stimulants.

Medications known to be associated with drug induced parkinsonism will not be allowed

for 6 months prior to screening and for the duration of the study, dopamine receptor

blockers (neuroleptics), metoclopramide and reserpine. All concomitant medications

reported at the time of the Screening visit and for the duration of participation are

recorded on the study medication logs.

1

8.2 Initiation of PD Medication

It is anticipated that PD participants will not require PD medications for at least 6 months

after Baseline. However, PD medications may be initiated at any time after enrollment at

the discretion of the participant or treating physician. The medication used is at the

discretion of the treating physician. The Investigator will document any new medications

or changes in medication at each study visit on the study medication logs.

1

9

PARTICIPATION IN CLINICAL TRIALS

It is understood that individuals may want to participate in therapeutic clinical trials. It is

preferred, but not required, that participants who choose to participate in clinical trials of

investigational therapeutics, begin their clinical trial following 12 months of participation in

PPMI. All participants who do enroll in a clinical trial may remain in the PPMI Clinical

study. PPMI will work collaboratively with the clinical trial sponsor to share PPMI study

data and encourage clinical trial participants to remain in PPMI Clinical in whatever capacity

possible. Contact the Site Management Core for further instruction and to determine whether

an in-person PPMI visit may be needed before the participant begins a therapeutic clinical

trial. For those studies testing a drug, the Investigator will document on the medication log

the study drug dosage, if applicable and known, and, if unknown, will report on the identity

of the study drug and dosage after it is unmasked. Other information pertaining to

participation in other clinical trials or observational studies may be documented in the PPMI

study database.

2

0

COSTS FOR PARTICIPATION

All research travel, assessments and tests will be provided with no cost to the study

participant.

PPMI

Version Date: 01Feb2021

Page 33 of 55


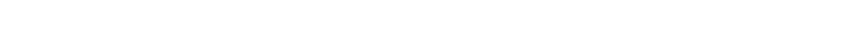


DocuSign Envelope ID: 706F2DD8-8346-4731-A419-FF8E7C48DF97

2

1

2

3

PAYMENT AND REIMBURSEMENT FOR PARTICIPATION

Participants will be paid for completed study visits based on the visit type. Participants who

require travel to the clinical site, or incur other costs associated with a study visit, will be

reimbursed according to the study reimbursement guidelines. Participants will have the

option to receive funds using either a pre-paid card, or direct deposit to a personal account.

2

PARTICIPANT WITHDRAWALS

Study participants will be informed during the consent process that they have the right to

withdraw from the study at any time without prejudice and may be withdrawn at the

Investigator’s or Sponsor’s discretion at any time. Any information that has already been

collected prior to the study participant’s withdrawal will not be removed.

2

ADVERSE EVENTS

2

3.1 Adverse Event Reporting Requirements

Site investigators and coordinators will be instructed to assess for adverse events at the study

visit when DaTscan imaging, lumbar puncture, or skin biopsy is conducted, as well as by

telephone 2 to 3 [business/working] days following such activity. Adverse experiences,

whether observed by the investigator, or elicited from or volunteered by the participant,

should be recorded on the Adverse Event Log. Events occurring outside of the study

procedure adverse event reporting period defined above do not require documentation for

study purposes (i.e., will not be listed on the Adverse Event Log).

Any adverse event ongoing at the 2 to 3 [business/working] day reporting telephone visit,

should be followed until resolution or stabilization. Adverse events reported following a

premature withdrawal or conclusion of participation visit should be followed not more than

3

0 days from last study procedure (i.e., SPECT imaging, lumbar puncture, skin biopsy).

Adverse events will be reported by the site as required by the site’s Institutional

Review/Ethics Board and to the Radiation Safety Committee, as applicable.

2

3.2 Serious Adverse Event Reporting Requirements

Serious adverse events pertaining to DaTscan imaging, lumbar puncture, or skin biopsy will

be reported as follows (see Operations Manual for detailed SAE reporting instructions):

a) Any serious adverse event occurring within 24 hours following the

DaTscan^TM^ injection will be documented on the Adverse Event Log and reported to

GE Healthcare using PPMI GE Healthcare SAE Form, whether assessed as related to

administration of DaTscan^TM^ or not.

b) Any serious adverse event occurring more than 24 hours following the

DaTscan^TM^ injection that is assessed as being related to the DaTscan^TM^ injection will

be documented on the Adverse Event Log and reported to GE Healthcare using PPMI

GE Healthcare SAE Form.

PPMI

Page 34 of 55

Version Date: 01Feb2021


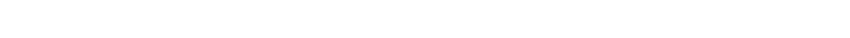


DocuSign Envelope ID: 706F2DD8-8346-4731-A419-FF8E7C48DF97

c) Any serious adverse event occurring up to 3 days following a lumbar puncture or skin

biopsy will be documented on the Adverse Event Log and may result in additional

follow up with the site.

d) The Investigator will comply with his/her local Institutional Review Board

(IRB)/Ethics Board, and Radiation Safety Committee (as applicable), regarding the

reporting of adverse experiences.

2

3.3 Adverse Event Definitions

Adverse Events (AE)

An AE is any undesirable experience occurring to a participant during study participation,

whether or not considered related to the study procedure.

Serious Adverse Event (SAE)

An SAE is an AE that is fatal or life-threatening, or results in hospitalization, prolongation

of hospitalization, persistent or significant disability/incapacity, or a congenital

anomaly/birth defect. A life-threatening AE is an AE that, in the view of the investigator,

places the participant at immediate risk of death from the reaction, as it occurred. Important

medical events that may not result in death, be life-threatening, or require hospitalization

may be considered an SAE when, based upon appropriate medical judgment, they may

jeopardize the participant and may require medical or surgical intervention to prevent one of

the outcomes listed in this definition.

Inpatient admission in the absence of a precipitating, treatment-emergent, clinical adverse

event is not participant to immediate reporting. For example:

•

Admission for treatment of a pre-existing condition not associated with the

development of a new adverse event.

•

•

Social admission (e.g., participant has no place to sleep).

Protocol-specific admission during a clinical study (e.g., for a procedure required by

another study protocol).

•

Optional admission not associated with a precipitating clinical adverse event (e.g.,

for elective cosmetic surgery).

Inpatient admission does not include the following:

•

•

•

•

•

•

•

•

•

Emergency Room/Accident and Emergency/Casualty Department visits

Outpatient/same-day/ambulatory procedures

Observation/short-stay units

Rehabilitation facilities

Hospice facilities

Respite care (e.g., caregiver relief)

Skilled nursing facilities

Nursing homes

Custodial care facilities

PPMI

Page 35 of 55

Version Date: 01Feb2021


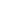

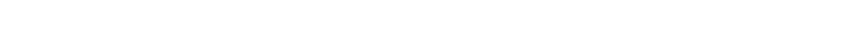


DocuSign Envelope ID: 706F2DD8-8346-4731-A419-FF8E7C48DF97

2

3.4 Assessing Relationship of Adverse Events

The assessment of the relationship of an AE to the imaging procedure, lumbar puncture, or

skin biopsy is a clinical decision based on all available information at the time the event is

being documented. The following definitions of the relationship between the AE (including

SAEs) and the study procedure should be considered:

•

•

•

Unrelated - No possible relationship

The temporal relationship between study procedure and the adverse event

onset/course is unreasonable or incompatible, or a causal relationship to study

procedure is implausible.

Unlikely - Not reasonably related, although a causal relationship cannot be ruled out.

While the temporal relationship between study procedure and the adverse event

onset/course does not preclude causality, there is a clear alternate cause that is more

likely to have caused the adverse event than the study procedure.

Possible - Causal relationship is uncertain

The temporal relationship between study procedure and the adverse event

onset/course is reasonable or unknown, and while other potential causes may not

exist, a causal relationship to the study procedure does not appear probable.

•

•

Probable - High degree of certainty for causal relationship

The temporal relationship between study procedure and the adverse event

onset/course is reasonable and other causes have been eliminated or are unlikely.

Definite - Causal relationship is certain

The temporal relationship between study procedure and the adverse event

onset/course is reasonable and other causes have been eliminated.

2

3.5 Assessing Intensity/Severity of Adverse Event

In addition to assessing the relationship of the adverse event to the study procedure, an

assessment is required of the intensity (severity) of the event. The following classifications

should be used:

•

•

•

Mild:

A mild AE is an AE, usually transient in nature and generally not interfering with

normal activities.

Moderate:

A moderate AE is an AE that is sufficiently discomforting to interfere with normal

activities.

Severe:

A severe AE is an AE that incapacitates the participant and prevents normal activities.

Note that a severe event is not necessarily a serious event. Nor must a serious event

necessarily be severe.

2

4

SIGNIFICANT STUDY EVENTS

There are important events that might occur during a participant’s follow up in the study,

PPMI

Version Date: 01Feb2021

Page 36 of 55


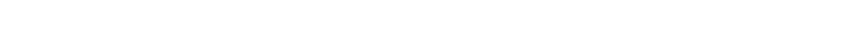


DocuSign Envelope ID: 706F2DD8-8346-4731-A419-FF8E7C48DF97

such as initiation of PD medication, new clinical diagnosis, an SAE, pregnancy, or death.

This information will be captured within the study database and may result in additional

follow up with the site. These events are fully described in the Operations Manual.

2

5

STUDY MONITORING AND SITE MANAGEMENT

The PPMI Steering Committee has the responsibility to monitor all procedures for safety,

GCP, and regulatory compliance. The study sites will be managed and overseen in an

ongoing manner to verify:

(a) The rights and well-being of human participants are protected.

(b) The reported study data are accurate, complete, and attributable.

(c) The conduct of the study follows the currently approved protocol/amendment(s), with

GCP, and with the applicable regulatory requirement(s).

2

6

PRIVACY AND CONFIDENTIALITY

Privacy of participants will be protected in that each person will have the option to voluntarily

choose whether to participate in this study. It is the responsibility of the site Investigator to

consider the participant’s privacy and confidentiality when completing study visits and

related protocol activities.

The Site Investigator must assure that the confidentiality of participants, including their

personal identity and personal medical information, will be maintained at all times. U.S.

sites have additional confidentiality obligations to study participants under the Health

Insurance Portability and Accountability Act (HIPAA), while European sites have additional

obligations under the EU General Data Protection Regulation (GDPR). Participants will be

identified by participant ID numbers on data forms and other study materials submitted to

the Site Management Core (SMC), the central laboratory, and central biorepository.

The Site Investigator will permit the study monitor or designated SMC representative to

review signed informed consent(s) and that portion of the participant’s medical record that

is directly related to the study (or provide certified copies of source documentation upon

request). This shall include all study relevant documentation including participant medical

history to verify eligibility, laboratory test result reports, admission/discharge summaries for

hospital admissions occurring while the participant is in the study, and autopsy reports for

deaths occurring during the study (when available). In addition, electronic document storage

will be maintained within the Florence electronic trial master file. Identifiable participant

information may be stored within this system, which has been validated and deemed

compatible with 21 CFR Part 11 requirements. Only study staff requiring access to related

study documentation will have permission to view identifiable information.

2

7

DATA AND SAMPLE SHARING AND STORAGE FOR FUTURE USE

Additional data collected for this study will be maintained and stored indefinitely at the study

Cores on secure, password protected systems. All study information (data and samples) will

be accessed only by those who require access as pertains to the individual’s role on the study.

All organizations responsible for data storage and review will observe the highest precautions

to ensure data integrity and security.

PPMI

Version Date: 01Feb2021

Page 37 of 55


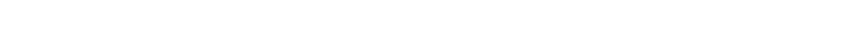


DocuSign Envelope ID: 706F2DD8-8346-4731-A419-FF8E7C48DF97

Data collected for this study may be transferred and shared across participating PPMI Cores

including the Data Management and Analytics Core at Blackfynn, LLC (Philadelphia, PA),

Indiana University PPMI Cores (Indianapolis, IN), the Site Management Core at the Institute

for Neurodegenerative Disorders (New Haven, CT), and the Statistical Core at the University

of Iowa (Iowa City, IA) for conducting analyses as pertains to the study including, but not

limited to, enrollment, compliance, study outcomes and, in combination from the data

received from PPMI Online and PPMI Remote studies, to enable modifications to the

predictive prodromal eligibility criteria. All PPMI data will be incorporated into a fully

harmonized PPMI database.

All data obtained during the conduct of PPMI Clinical will be sent to the Laboratory of Neuro

Imaging (LONI) in Los Angeles, California to be stored indefinitely for research purposes.

Research data will be made available to researchers to conduct analyses related to PD and

other disorders. Researchers will be required to comply with the PPMI data agreement to

receive data. All personally identifiable information will be removed before it is shared

outside the study.

Research biosamples will be shipped and stored indefinitely for research purposes at the

Biorepository Cores at Indiana University School of Medicine, BioRep in Milan, Italy and

Tel Aviv Sourasky Medical Center in Tel Aviv, Israel. Research specimens will be made

available to researchers to conduct analyses related to PD and other disorders through an

application process to the Biospecimen Review Committee (BRC). All personally

identifiable information will be removed before it is shared outside the study.

2

8

ANALYSIS PLAN

The overall goal of PPMI is to identify markers of disease progression to inform clinical

trials of therapies to reduce progression of PD disability. Correspondingly, all primary and

secondary analyses of the PPMI data will focus on this goal. However, due to the rich nature

of data collected as part of this study, many additional exploratory analyses will be examined

throughout the study – both within and outside of the primary study steering committee.

Throughout the course of the study, analyses will be periodically updated to examine and

compare baseline characteristics among the various subsets enrolled into the study.

Continuous variables will be examined using a t-test and dichotomous variables will be

examined using a chi-square test. Appropriate assumptions will be assessed for each

comparison and necessary adjustments (i.e., transformations) will be made prior to analysis.

2

8.1 Primary Objectives

2

8.1.1 Comparison of progression biomarkers among cohorts/subsets

Use clinical and biological data to estimate the mean rates of change and variability around

the mean of clinical, digital, imaging, biological, and genetic outcomes in study

participants with PD diagnosis [including patients with a LRRK2, GBA, SNCA, or rare

genetic mutations (such as Parkin or Pink1) and individuals with prodromal Parkinson

disease [including individuals with RBD, olfactory loss, LRRK2, GBA, SNCA, or rare

PPMI

Page 38 of 55

Version Date: 01Feb2021


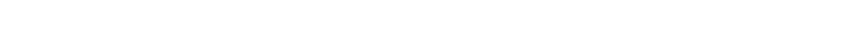


DocuSign Envelope ID: 706F2DD8-8346-4731-A419-FF8E7C48DF97

genetic mutations (such as Parkin or Pink1) and/or other risk factors for PD with and

without DAT deficit and in healthy participants.

Due to the large number of progression endpoints possible for consideration, a substantial

number of analyses will be conducted to examine the change and variability over time.

These analyses will include standard logistic, linear, and longitudinal models, and many

other proposed approaches for assessing these data. Primary interest will focus on well-

known and accepted measures of disease progression (such as the MDS-UPDRS). But, an

examination and potential development of novel progression endpoints to better

characterize disease progression over time in this heterogeneous cohort will be considered.

2

8.1.2 Examination of PD subsets

Analyses will also be conducted to assess whether there are subgroup differences observed

with respect to disease progression. Analyses will confirm existing and identify novel

clinical, digital, imaging, biologic, and genetic PD progression markers to identify

quantitative individual measures or combinations of measures that demonstrate optimum

interval change in study participants with PD diagnosis [including patients with a LRRK2,

GBA, SNCA or rare genetic mutations (such as Parkin or Pink1)] and individuals with

prodromal Parkinson disease [including individuals with RBD, olfactory loss, LRRK2,

GBA, or rare genetic mutations (such as Parkin or Pink1) and/or other risk factors for PD

with and without DAT deficit in comparison to healthy controls or in subsets of study

participants with PD diagnosis or prodromal PD defined by baseline assessments,

progression milestones and/or rate of clinical, digital, imaging, biologic, and genetic

change, or other measures.

2

8.1.3 Analysis of prodromal participants phenoconversion

Evaluate the probability of phenoconversion for PD for individuals with prodromal PD

enrolled in the prodromal cohorts [including individuals with RBD, olfactory loss, LRRK2,

GBA, SNCA, or rate genetic mutations (such as Parkin or Pink1) and/or other risk factors

for PD with and without DAT deficit. This analysis will involve estimating the number and

percentage of prodromal participants that meet criteria for phenoconversion at a number of

observed time points. For each period of time, the percentage and a 95% confidence

interval will be reported.

2

8.2 Secondary Objectives

2

8.2.1 Ancillary biomarker studies

Conduct preliminary clinical, digital, imaging, biologic, and genetic markers verification

studies on promising biological markers in study subsets using stored collected samples.

A series of ancillary analyses will be conducted to verify known and novel proposed PD

biomarkers. These studies will vary substantially depending on the type of marker. But, as

much as possible, the methods and analysis plans for all verification studies will be

reviewed centrally by the PPMI steering committee in advance of implementation.

PPMI

Page 39 of 55

Version Date: 01Feb2021


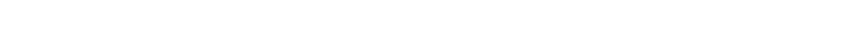


DocuSign Envelope ID: 706F2DD8-8346-4731-A419-FF8E7C48DF97

2

8.2.2 Compare genetic and idiopathic PD

Compare biomarker signatures for study participants with PD diagnosis without known

genetic mutation to those with known genetic mutation [including LRRK2, GBA, SNCA,

or rare genetic mutations (such as Parkin or Pink1)]. These analyses will initially involve

a high-level comparison of whether PD progression over time differs among those with

and without a known genetic mutation. Subsequent analyses will be implemented in much

the same way as above, with the exception that models will implicitly assume and examine

potential interactions between presence or absence of a known genetic mutation and each

of the potential progression markers considered in the various models.

2

2

8.2.3 Compare Prodromal and PD

Compare biomarker signatures for study participants with PD diagnoses to individuals with

prodromal PD enrolled in the prodromal cohorts [including individuals with RBD,

olfactory loss, LRRK2, GBA, SNCA, or rate genetic mutations (such as Parkin or Pink1)]

and/or other risk factors for PD with and without DAT deficit and between prodromal

participants who phenoconvert and those that have not phenoconverted.

8.2.4 Model Prodromal progression and predictors of phenoconversion

Develop and test risk paradigms to establish the sequence of early prodromal events

(clinical, imaging, biologic changes) in individuals with prodromal PD enrolled in the

prodromal cohorts [including individuals with RBD, olfactory loss, LRRK2, GBA, SNCA,

or rare genetic mutations (such as Parkin or Pink1) and/or other risk factors for PD with

and without DAT deficit] including testing early signal of risk in the associated PPMI

Online and PPMI Remote studies.

PPMI

Page 40 of 55

Version Date: 01Feb2021


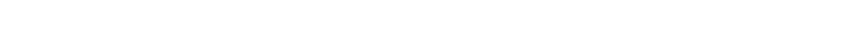


DocuSign Envelope ID: 706F2DD8-8346-4731-A419-FF8E7C48DF97

2

9

REFERENCES

1

.

Chen-Plotkin AS, Albin R, Alcalay R, Babcock D, Bajaj V, Bowman D, et al. Finding

useful biomarkers for Parkinson's disease. Sci Transl Med. 2018;10(454).

Espay AJ, Lang AE. Parkinson Diseases in the 2020s and Beyond: Replacing Clinico-

2

.

Pathologic Convergence With Systems Biology Divergence. J Parkinsons Dis. 2018;8(s1):S59-

S64.

3

.

Obeso JA, Stamelou M, Goetz CG, Poewe W, Lang AE, Weintraub D, et al. Past, present,

and future of Parkinson's disease: A special essay on the 200th Anniversary of the Shaking Palsy.

Mov Disord. 2017;32(9):1264-310.

4

.

Marek K, Chowdhury S, Siderowf A, Lasch S, Coffey CS, Caspell-Garcia C, et al. The

Parkinson's progression markers initiative (PPMI) - establishing a PD biomarker cohort. Ann Clin

Transl Neurol. 2018;5(12):1460-77.

5

.

Parkinson Progression Marker I. The Parkinson Progression Marker Initiative (PPMI).

Prog Neurobiol. 2011;95(4):629-35.

Foroud T, Smith D, Jackson J, Verbrugge J, Halter C, Wetherill L, et al. Novel recruitment

strategy to enrich for LRRK2 mutation carriers. Mol Genet Genomic Med. 2015;3(5):404-12.

Jennings D, Siderowf A, Stern M, Seibyl J, Eberly S, Oakes D, et al. Imaging prodromal

Parkinson disease: the Parkinson Associated Risk Syndrome Study. Neurology.

6

.

7

.

2

8

014;83(19):1739-46.

. Marek K, Seibyl J, Eberly S, Oakes D, Shoulson I, Lang AE, et al. Longitudinal follow-up

of SWEDD subjects in the PRECEPT Study. Neurology. 2014;82(20):1791-7.

Prakash N, Caspell-Garcia C, Coffey C, Siderowf A, Tanner CM, Kieburtz K, et al.

9

.

Feasibility and safety of lumbar puncture in the Parkinson's disease research participants:

Parkinson's Progression Marker Initiative (PPMI). Parkinsonism Relat Disord. 2019;62:201-9.

1

0.

Simuni T, Caspell-Garcia C, Coffey CS, Weintraub D, Mollenhauer B, Lasch S, et al.

Baseline prevalence and longitudinal evolution of non-motor symptoms in early Parkinson's

disease: the PPMI cohort. J Neurol Neurosurg Psychiatry. 2018;89(1):78-88.

1

1.

Simuni T, Siderowf A, Lasch S, Coffey CS, Caspell-Garcia C, Jennings D, et al.

Longitudinal Change of Clinical and Biological Measures in Early Parkinson's Disease:

Parkinson's Progression Markers Initiative Cohort. Mov Disord. 2018;33(5):771-82.

1

2.

and the course of prodromal Parkinson's disease. Mov Disord. 2017;32(11):1640-5.

3. Chahine LM, Iranzo A, Fernandez-Arcos A, Simuni T, Seedorff N, Caspell-Garcia C, et

Weintraub D, Chahine LM, Hawkins KA, Siderowf A, Eberly S, Oakes D, et al. Cognition

1

al. Basic clinical features do not predict dopamine transporter binding in idiopathic REM behavior

disorder. NPJ Parkinsons Dis. 2019;5:2.

1

4.

Simuni T, Long JD, Caspell-Garcia C, Coffey CS, Lasch S, Tanner CM, et al. Predictors

of time to initiation of symptomatic therapy in early Parkinson's disease. Ann Clin Transl Neurol.

2

1

016;3(7):482-94.

5. Conrado DJ, Nicholas T, Tsai K, Macha S, Sinha V, Stone J, et al. Dopamine Transporter

Neuroimaging as an Enrichment Biomarker in Early Parkinson's Disease Clinical Trials: A Disease

Progression Modeling Analysis. Clin Transl Sci. 2018;11(1):63-70.

1

6.

Stephenson D, Hill D, Cedarbaum JM, Tome M, Vamvakas S, Romero K, et al. The

Qualification of an Enrichment Biomarker for Clinical Trials Targeting Early Stages of Parkinson's

Disease. J Parkinsons Dis. 2019;9(3):553-63.

PPMI

Page 41 of 55

Version Date: 01Feb2021


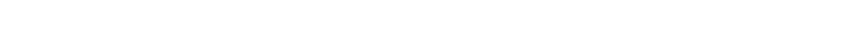


DocuSign Envelope ID: 706F2DD8-8346-4731-A419-FF8E7C48DF97

1

7.

Kang JH, Mollenhauer B, Coffey CS, Toledo JB, Weintraub D, Galasko DR, et al. CSF

biomarkers associated with disease heterogeneity in early Parkinson's disease: the Parkinson's

Progression Markers Initiative study. Acta Neuropathol. 2016;131(6):935-49.

1

8.

Mollenhauer B, Caspell-Garcia CJ, Coffey CS, Taylor P, Shaw LM, Trojanowski JQ, et al.

Longitudinal CSF biomarkers in patients with early Parkinson disease and healthy controls.

Neurology. 2017;89(19):1959-69.

1

9.

Mollenhauer B, Caspell-Garcia CJ, Coffey CS, Taylor P, Singleton A, Shaw LM, et al.

Longitudinal analyses of cerebrospinal fluid alpha-Synuclein in prodromal and early Parkinson's

disease. Mov Disord. 2019.

2

0.

Nalls MA, McLean CY, Rick J, Eberly S, Hutten SJ, Gwinn K, et al. Diagnosis of

Parkinson's disease on the basis of clinical and genetic classification: a population-based modelling

study. Lancet Neurol. 2015;14(10):1002-9.

2

1.

Dadar M, Zeighami Y, Yau Y, Fereshtehnejad SM, Maranzano J, Postuma RB, et al. White

matter hyperintensities are linked to future cognitive decline in de novo Parkinson's disease

patients. Neuroimage Clin. 2018;20:892-900.

2

2.

Pasquini J, Ceravolo R, Brooks DJ, Bonuccelli U, Pavese N. Progressive loss of raphe

nuclei serotonin transporter in early Parkinson's disease: A longitudinal (123)I-FP-CIT SPECT

study. Parkinsonism Relat Disord. 2019.

2

3.

Zhang Y, Wu IW, Tosun D, Foster E, Schuff N, Parkinson's Progression Markers I.

Progression of Regional Microstructural Degeneration in Parkinson's Disease: A Multicenter

Diffusion Tensor Imaging Study. PLoS One. 2016;11(10):e0165540.

2

4.

Kamps S, van den Heuvel OA, van der Werf YD, Berendse HW, Weintraub D, Vriend C.

Smaller subcortical volume in Parkinson patients with rapid eye movement sleep behavior

disorder. Brain Imaging Behav. 2019;13(5):1352-60.

2

5.

Caspell-Garcia C, Simuni T, Tosun-Turgut D, Wu IW, Zhang Y, Nalls M, et al. Multiple

modality biomarker prediction of cognitive impairment in prospectively followed de novo

Parkinson disease. PLoS One. 2017;12(5):e0175674.

2

6.

Chahine LM, Xie SX, Simuni T, Tran B, Postuma R, Amara A, et al. Longitudinal changes

in cognition in early Parkinson's disease patients with REM sleep behavior disorder. Parkinsonism

Relat Disord. 2016;27:102-6.

2

7.

Amara AW, Chahine LM, Caspell-Garcia C, Long JD, Coffey C, Hogl B, et al.

Longitudinal assessment of excessive daytime sleepiness in early Parkinson's disease. J Neurol

Neurosurg Psychiatry. 2017;88(8):653-62.

2

8.

LRRK2 mutation carriers resistant to Parkinson disease. Ann Neurol. 2019;85(4):593-9.

9. Koros C, Simitsi A, Prentakis A, Beratis I, Papadimitriou D, Kontaxopoulou D, et al. 123I-

FP-CIT SPECT [(123) I-2beta-carbomethoxy-3beta-(4-iodophenyl)-N-(3-fluoropropyl)

Bakshi R, Macklin EA, Logan R, Zorlu MM, Xia N, Crotty GF, et al. Higher urate in

2

nortropane single photon emission computed tomography] Imaging in a p.A53T alpha-synuclein

Parkinson's disease cohort versus Parkinson's disease. Mov Disord. 2018;33(11):1734-9.

3

0.

Latourelle JC, Beste MT, Hadzi TC, Miller RE, Oppenheim JN, Valko MP, et al. Large-

scale identification of clinical and genetic predictors of motor progression in patients with newly

diagnosed Parkinson's disease: a longitudinal cohort study and validation. Lancet Neurol.

2

3

017;16(11):908-16.

1. Berg D, Postuma RB, Adler CH, Bloem BR, Chan P, Dubois B, et al. MDS research criteria

for prodromal Parkinson's disease. Mov Disord. 2015;30(12):1600-11.

PPMI

Page 42 of 55

Version Date: 01Feb2021


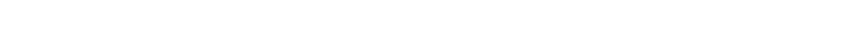


DocuSign Envelope ID: 706F2DD8-8346-4731-A419-FF8E7C48DF97

3

2.

research criteria for prodromal Parkinson's disease. Mov Disord. 2019;34(10):1464-70.

3. Iranzo A, Santamaria J, Valldeoriola F, Serradell M, Salamero M, Gaig C, et al. Dopamine

Heinzel S, Berg D, Gasser T, Chen H, Yao C, Postuma RB, et al. Update of the MDS

3

transporter imaging deficit predicts early transition to synucleinopathy in idiopathic rapid eye

movement sleep behavior disorder. Ann Neurol. 2017;82(3):419-28.

3

4.

Jennings D, Siderowf A, Stern M, Seibyl J, Eberly S, Oakes D, et al. Conversion to

Parkinson Disease in the PARS Hyposmic and Dopamine Transporter-Deficit Prodromal Cohort.

JAMA Neurol. 2017;74(8):933-40.

3

5.

Simuni T, Uribe L, Cho HR, Caspell-Garcia C, Coffey CS, Siderowf A, et al. Clinical and

dopamine transporter imaging characteristics of non-manifest LRRK2 and GBA mutation carriers

in the Parkinson's Progression Markers Initiative (PPMI): a cross-sectional study. Lancet Neurol.

2

3

020;19(1):71-80.

6. Stephenson D, Hu MT, Romero K, Breen K, Burn D, Ben-Shlomo Y, et al. Precompetitive

Data Sharing as a Catalyst to Address Unmet Needs in Parkinson's Disease. J Parkinsons Dis.

2015;5(3):581-94.

PPMI

Page 43 of 55

Version Date: 01Feb2021


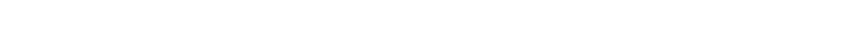


DocuSign Envelope ID: 706F2DD8-8346-4731-A419-FF8E7C48DF97

3

0

APPENDIX 1 – Healthy Control Schedule Years 0-5

PPMI

Version Date: 01Feb2021

Page 44 of 55


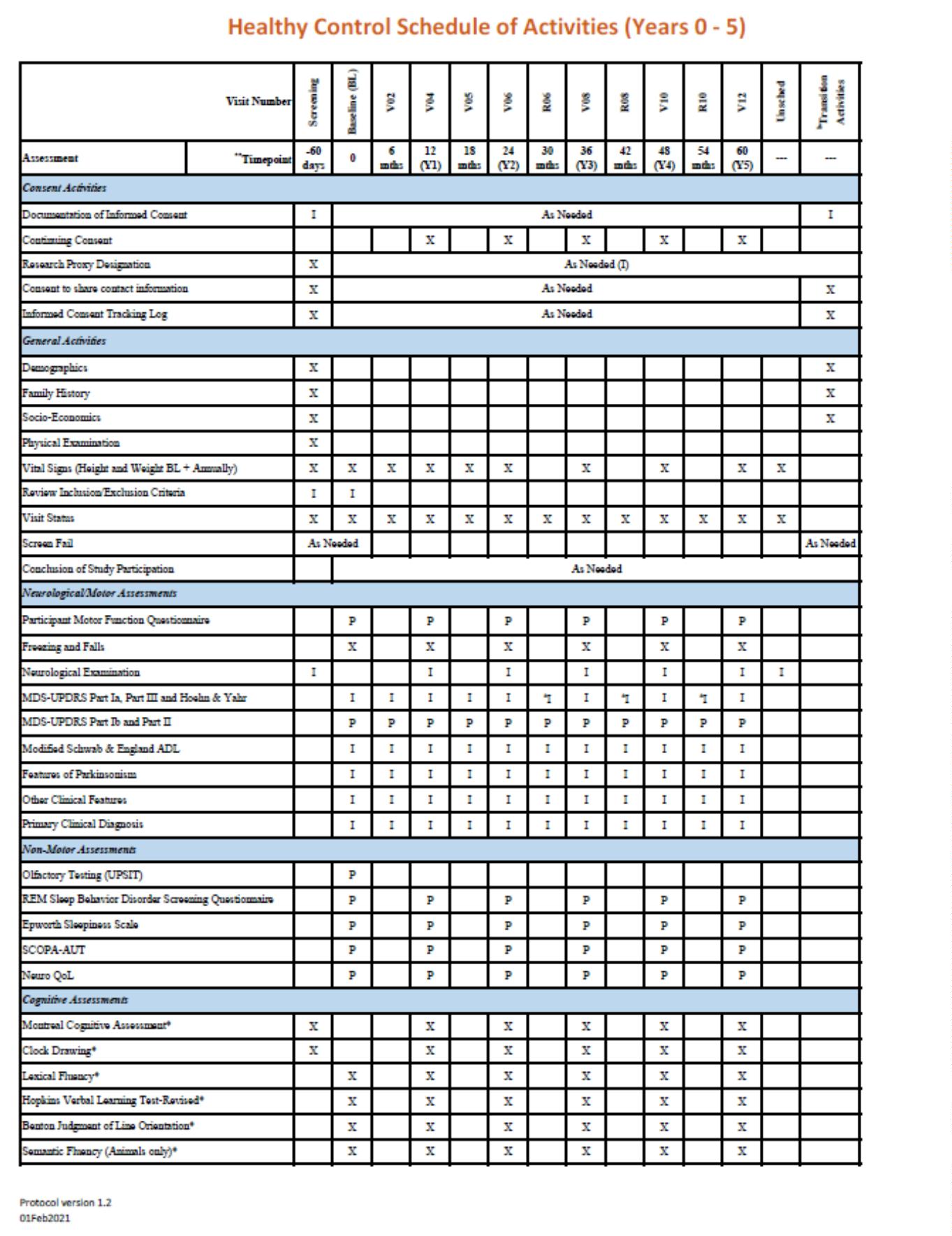

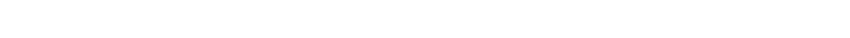


DocuSign Envelope ID: 706F2DD8-8346-4731-A419-FF8E7C48DF97

PPMI

Page 45 of 55

Version Date: 01Feb2021


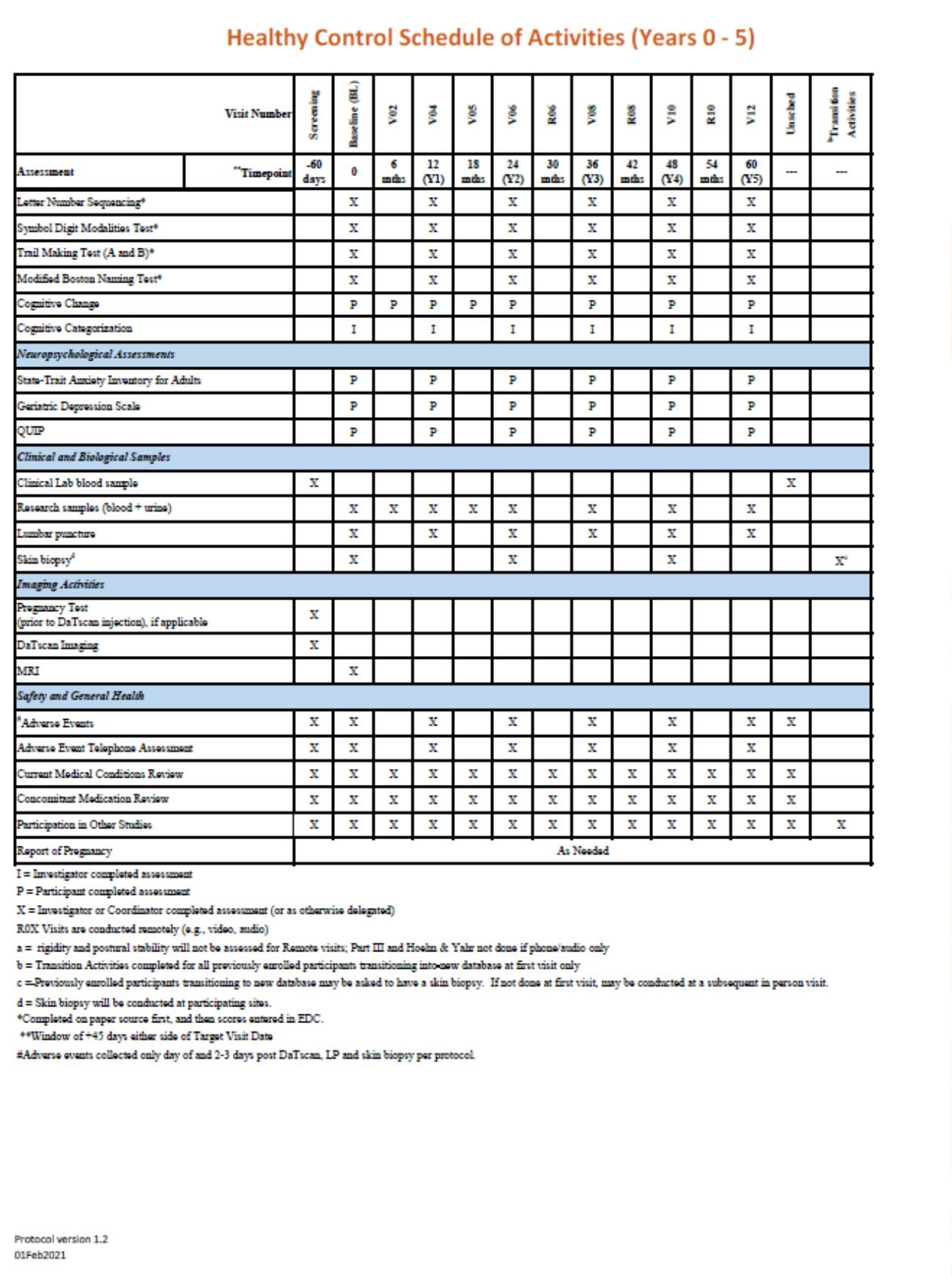

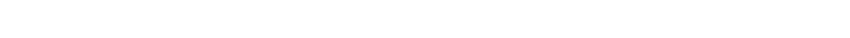


DocuSign Envelope ID: 706F2DD8-8346-4731-A419-FF8E7C48DF97

3

1

APPENDIX 2 – Prodromal Schedule Years 0 – 5

PPMI

Version Date: 01Feb2021

Page 46 of 55


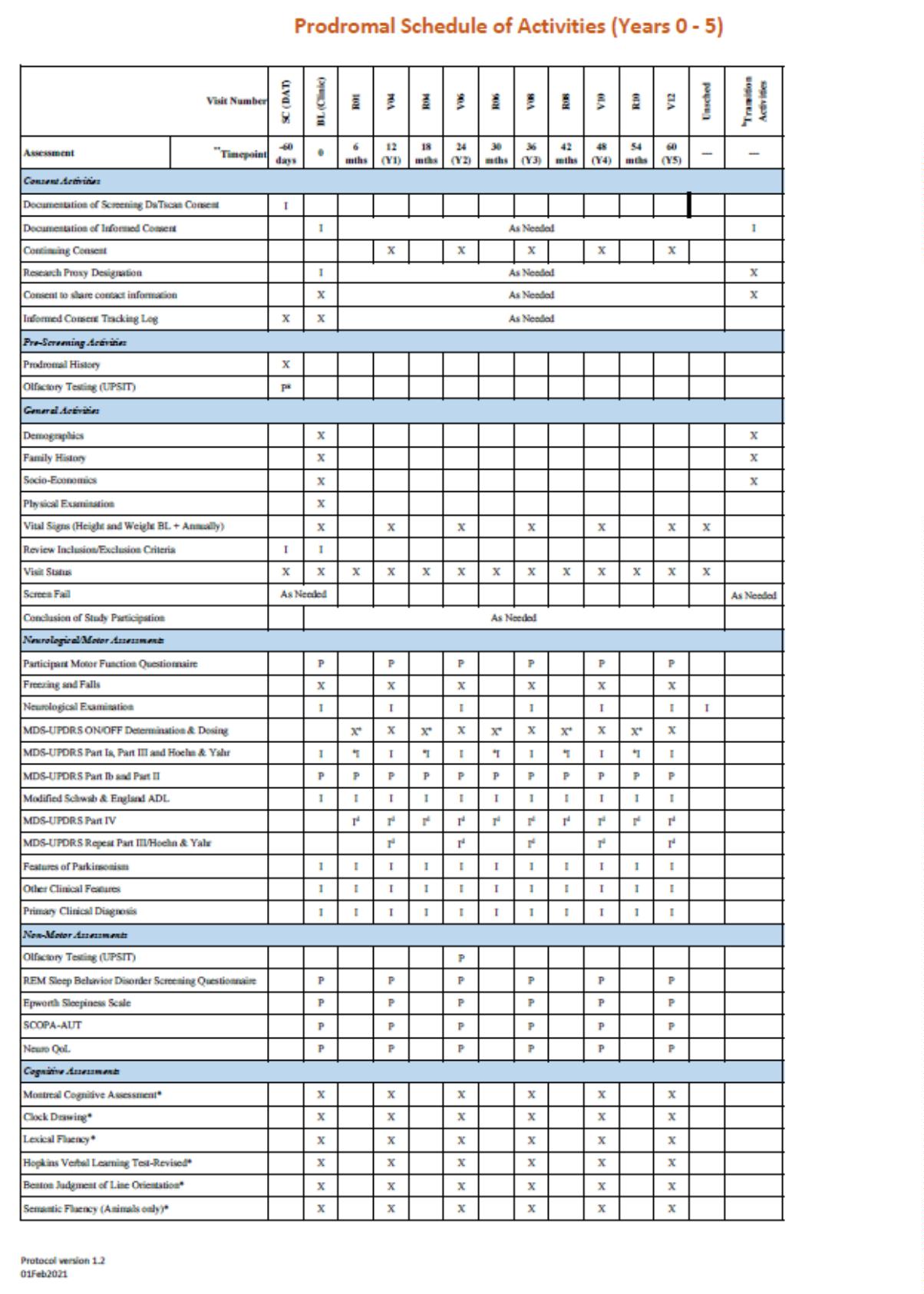

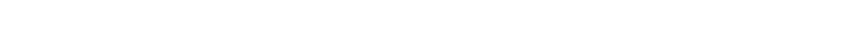


DocuSign Envelope ID: 706F2DD8-8346-4731-A419-FF8E7C48DF97

PPMI

Page 47 of 55

Version Date: 01Feb2021


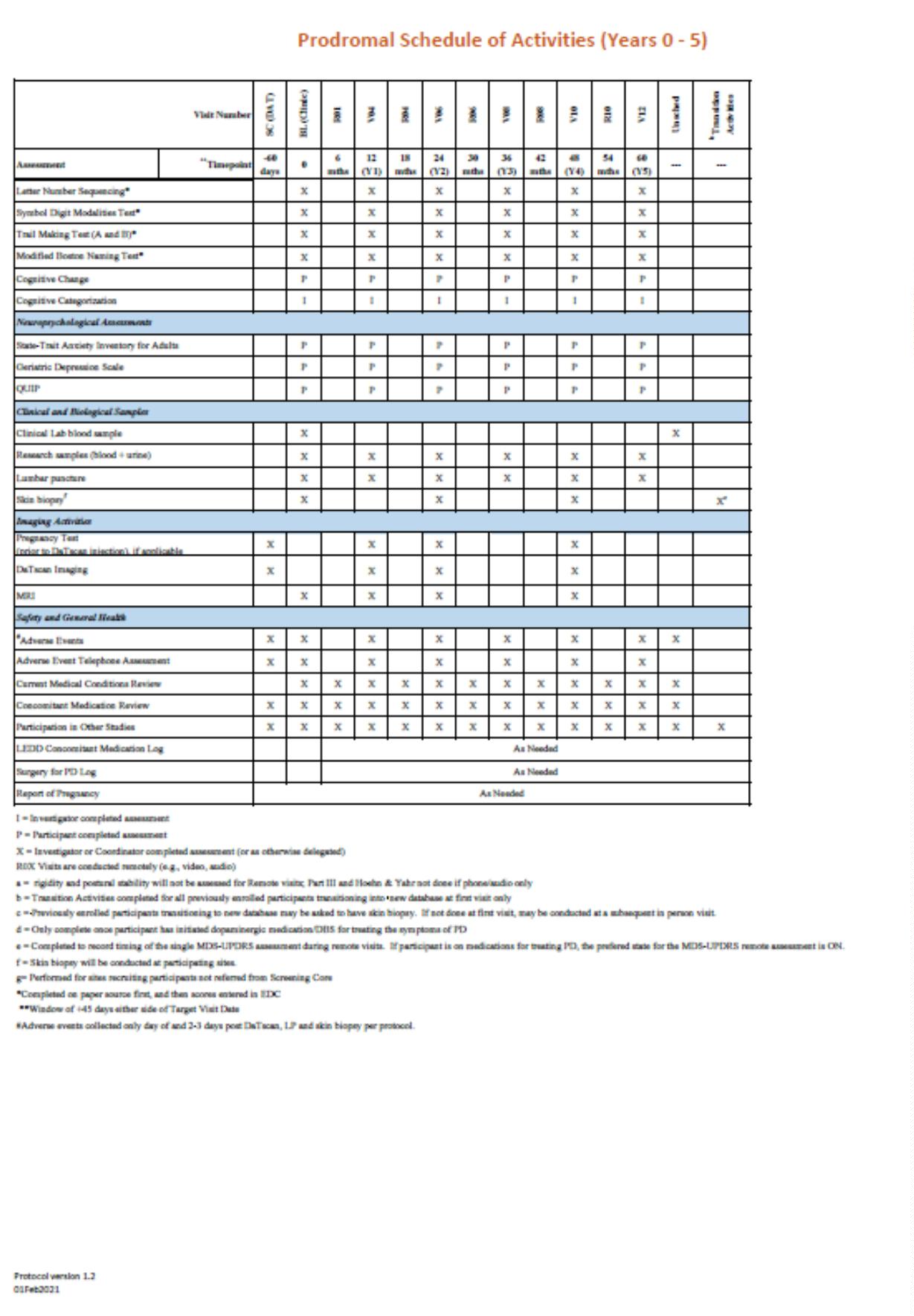

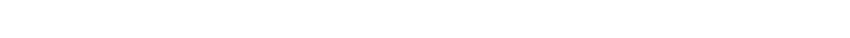


DocuSign Envelope ID: 706F2DD8-8346-4731-A419-FF8E7C48DF97

3

2

APPENDIX 3 – PD / PD Genetic Schedule Years 0 – 5

PPMI

Version Date: 01Feb2021

Page 48 of 55


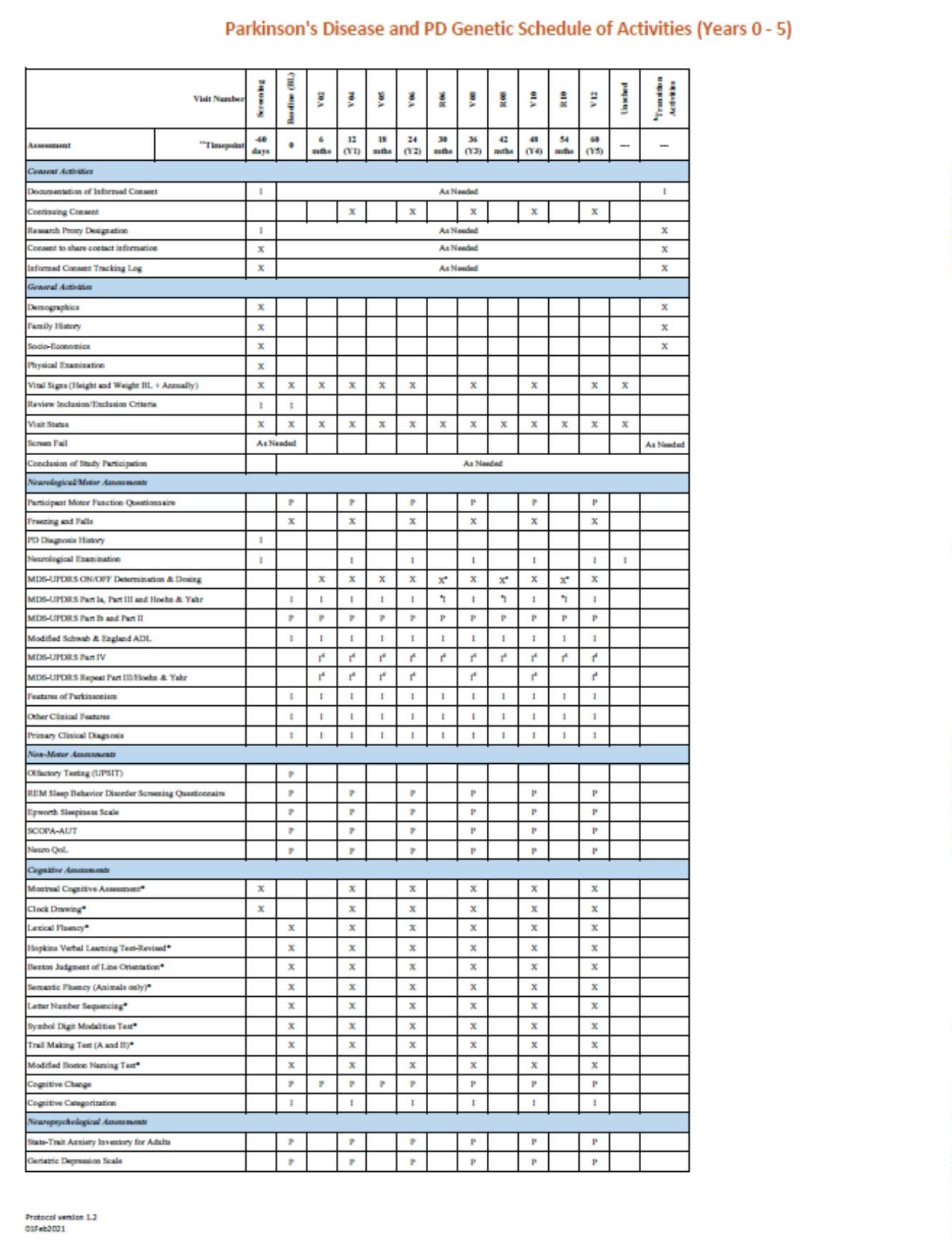

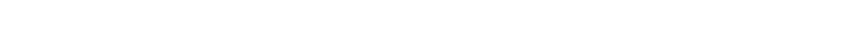


DocuSign Envelope ID: 706F2DD8-8346-4731-A419-FF8E7C48DF97

PPMI

Page 49 of 55

Version Date: 01Feb2021


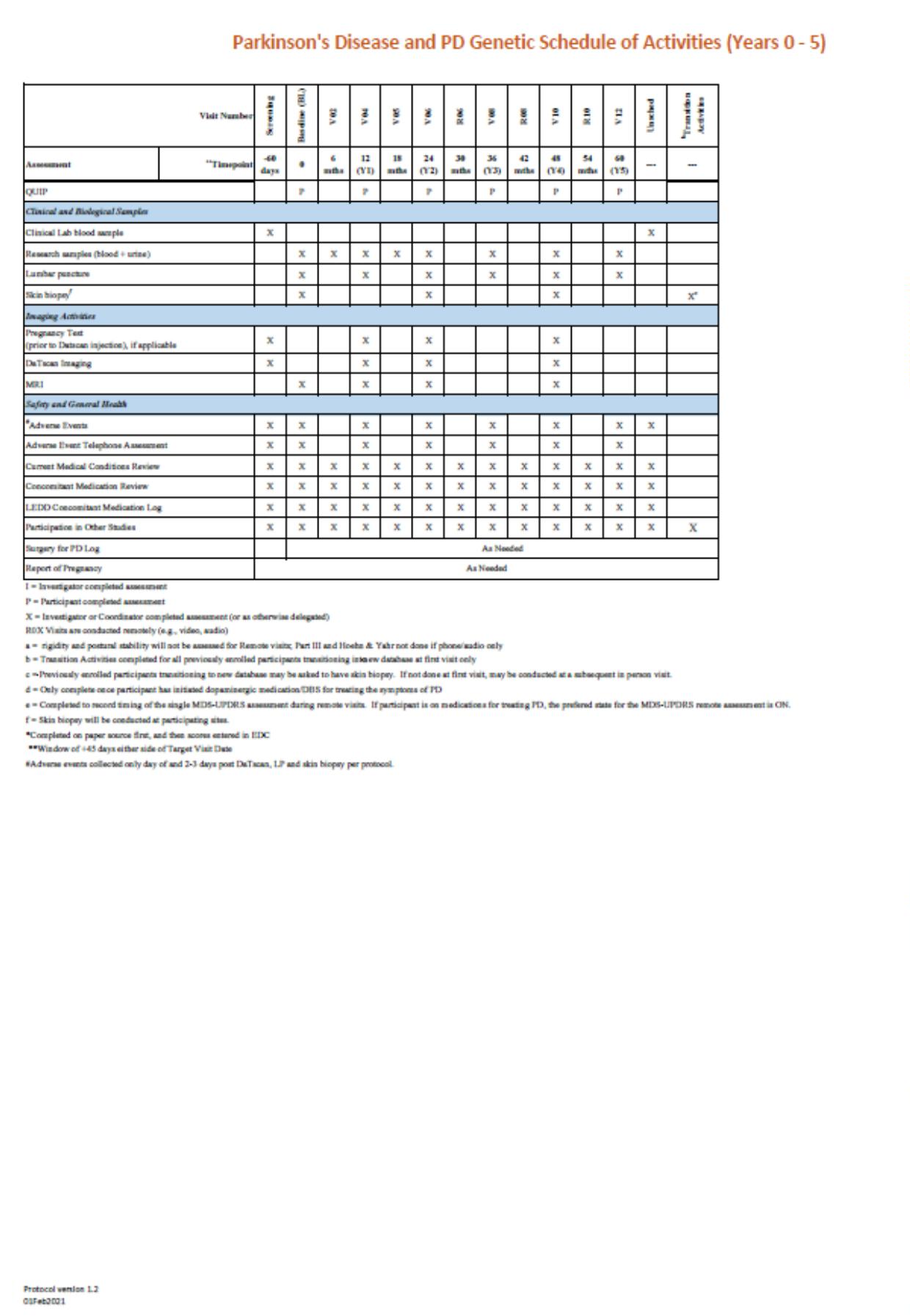

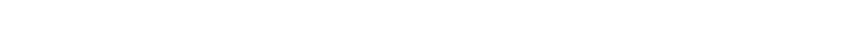


DocuSign Envelope ID: 706F2DD8-8346-4731-A419-FF8E7C48DF97

3

3

APPENDIX 4 – Healthy Control Schedule Years 6-13

PPMI

Version Date: 01Feb2021

Page 50 of 55


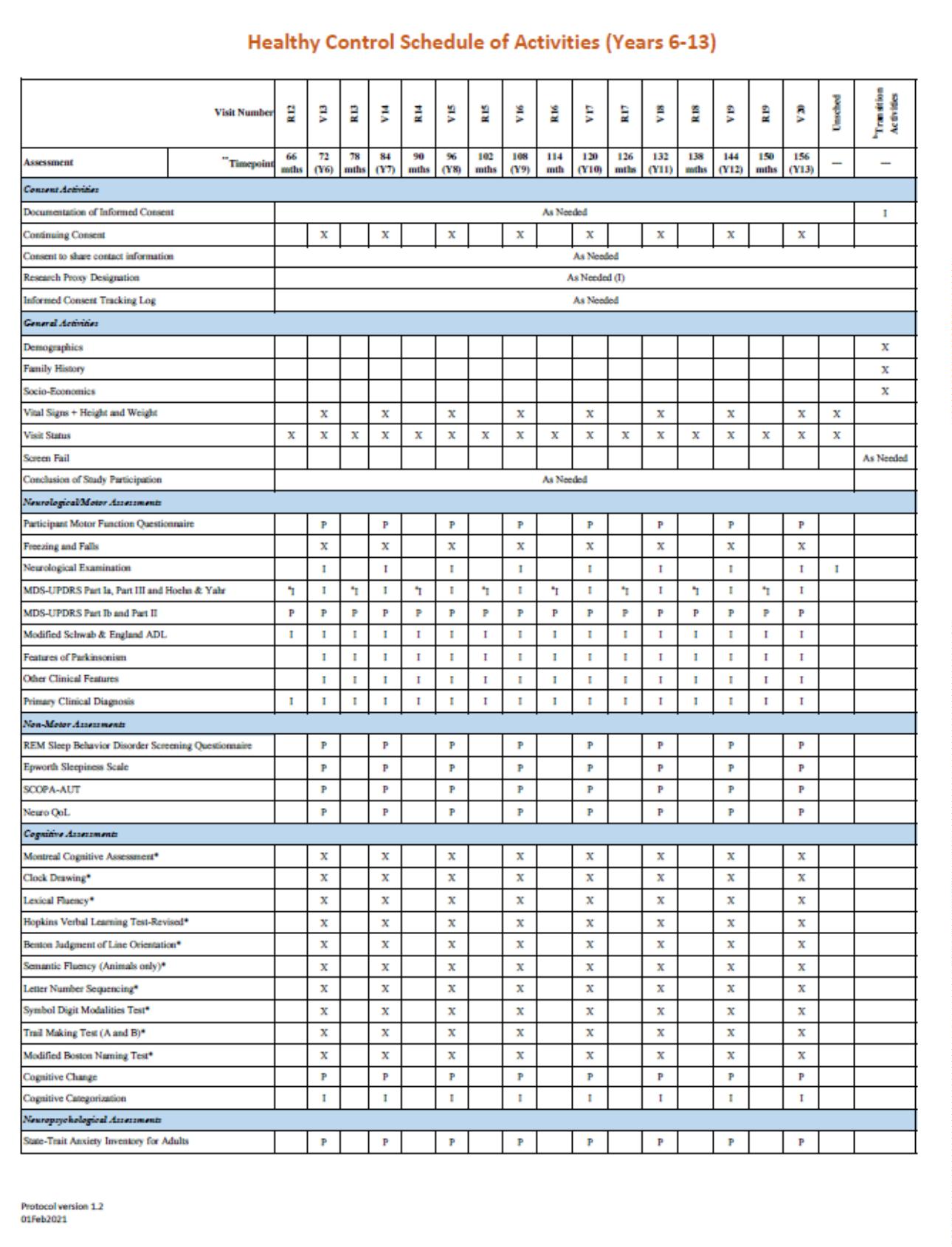

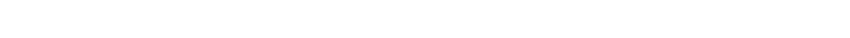


DocuSign Envelope ID: 706F2DD8-8346-4731-A419-FF8E7C48DF97

PPMI

Page 51 of 55

Version Date: 01Feb2021


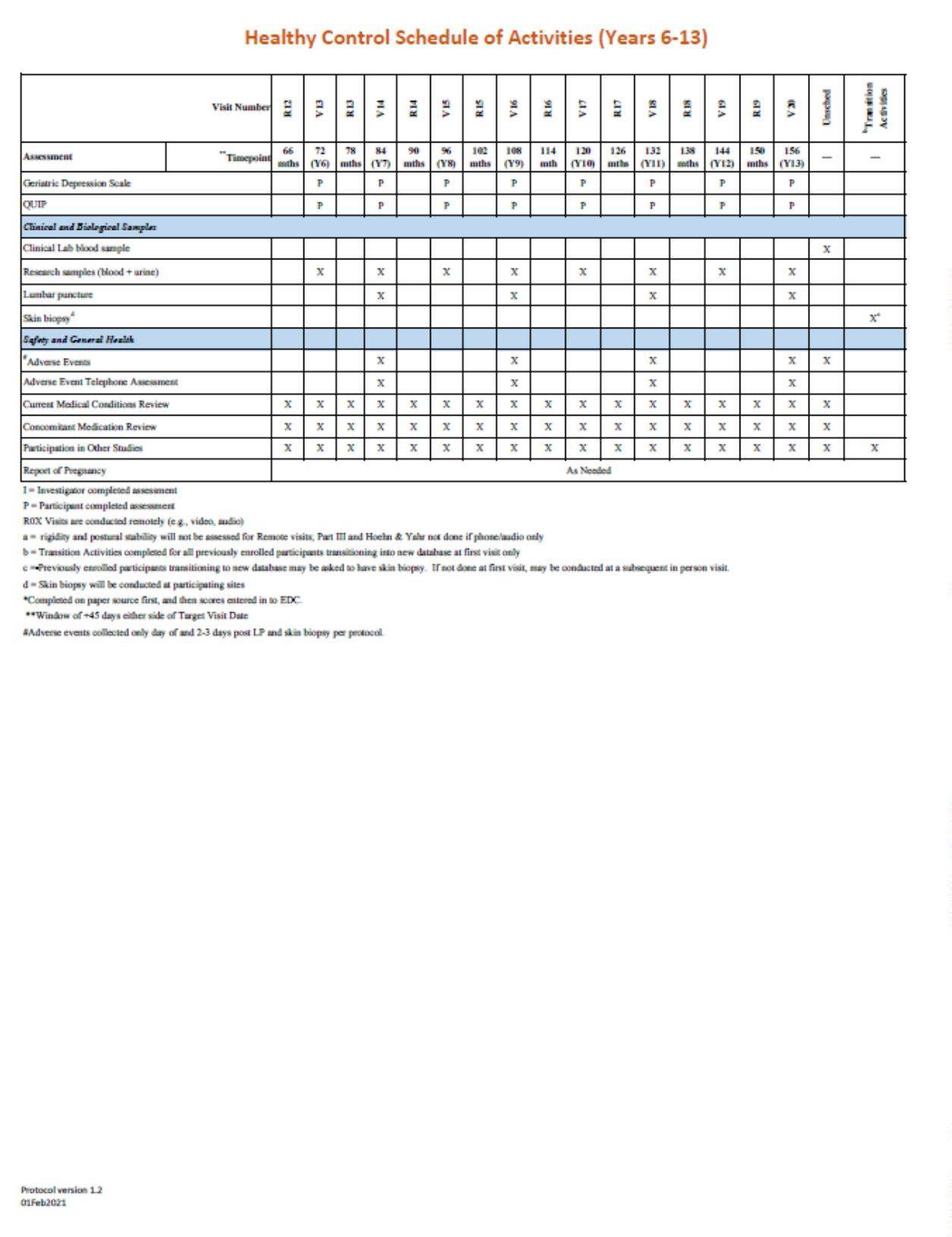

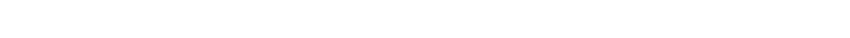


DocuSign Envelope ID: 706F2DD8-8346-4731-A419-FF8E7C48DF97

3

4

APPENDIX 5 – Prodromal Schedule Years 6-13

PPMI

Version Date: 01Feb2021

Page 52 of 55


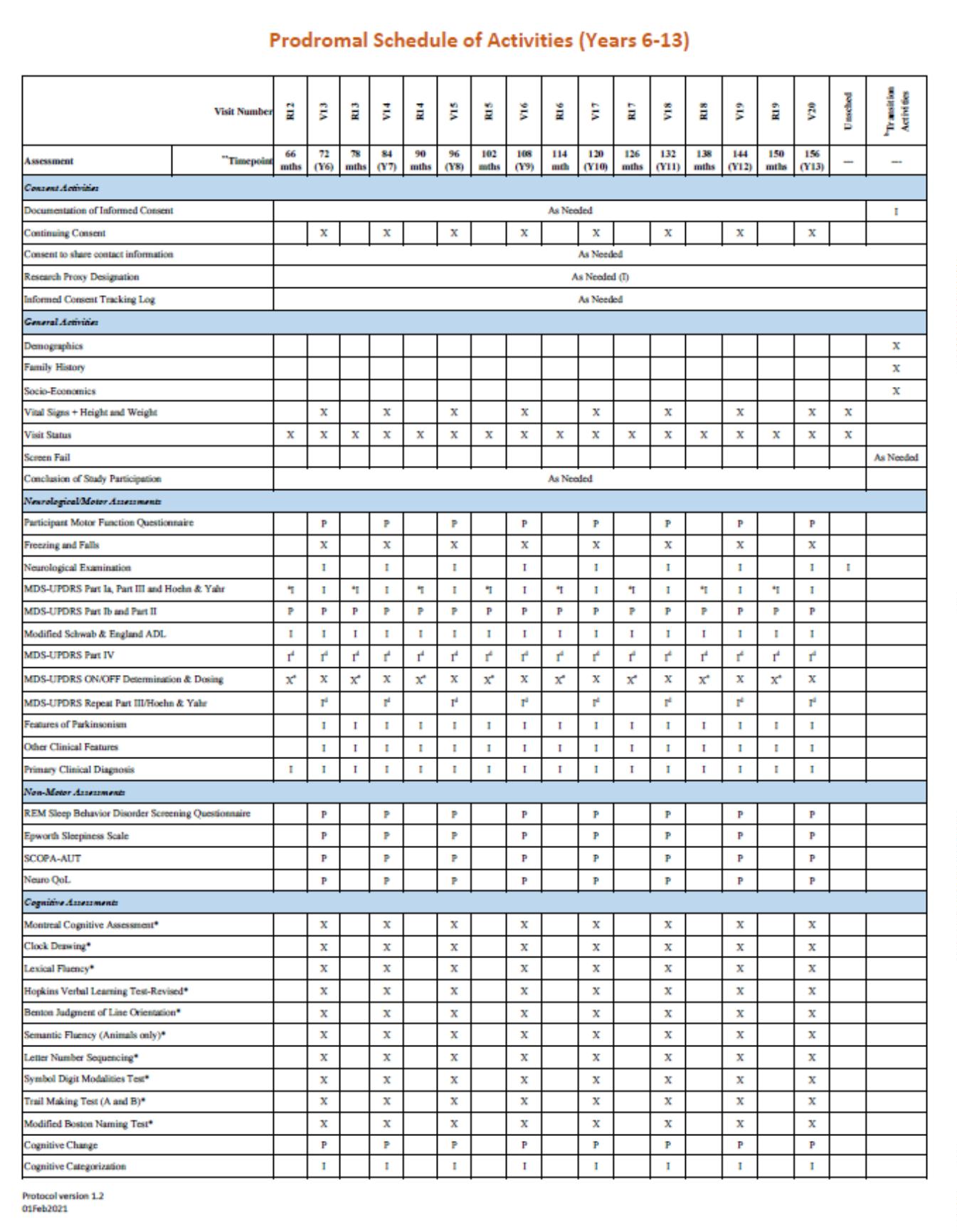

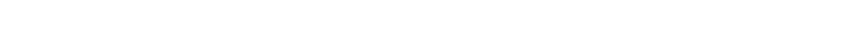


DocuSign Envelope ID: 706F2DD8-8346-4731-A419-FF8E7C48DF97

PPMI

Page 53 of 55

Version Date: 01Feb2021


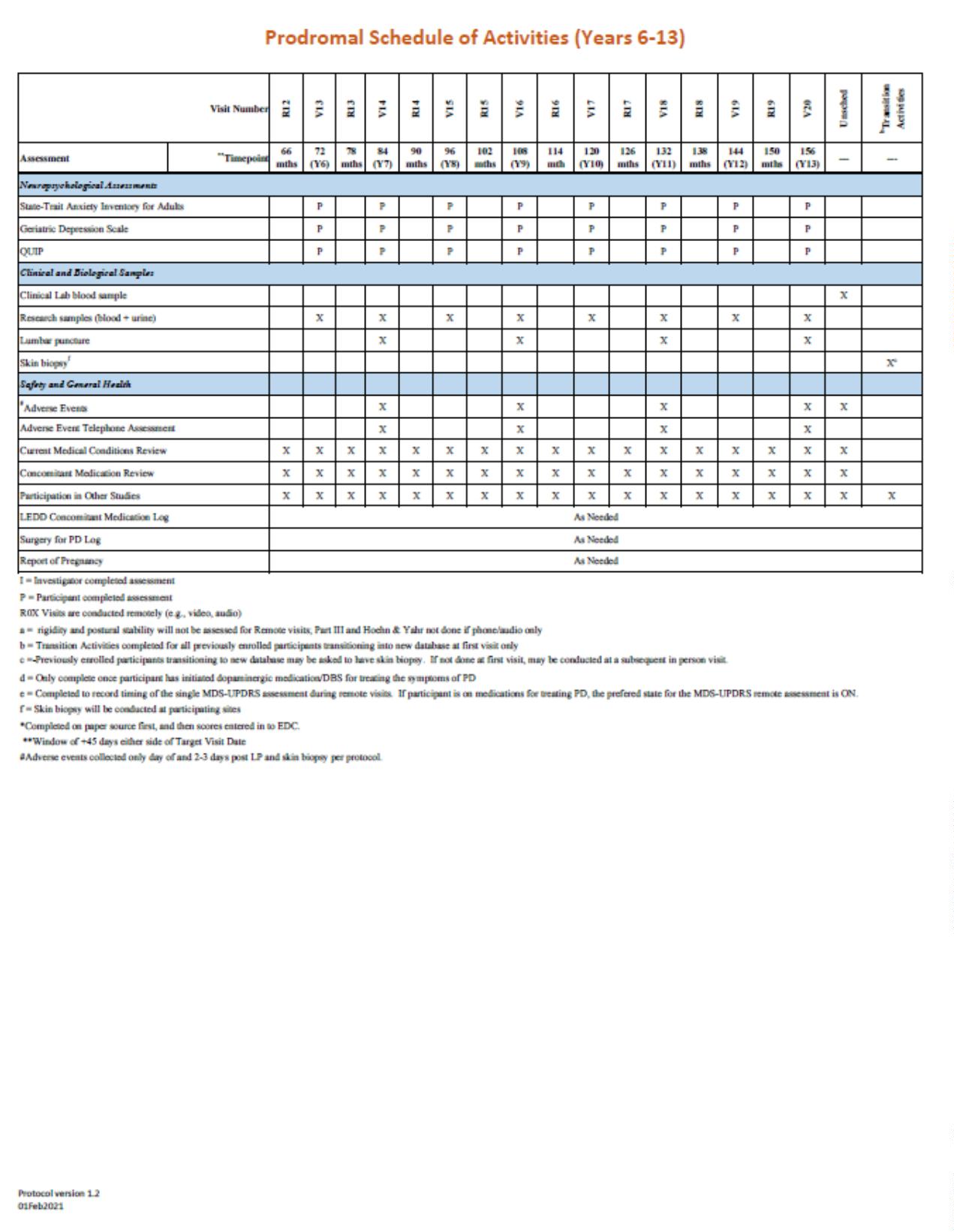

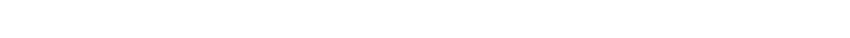


DocuSign Envelope ID: 706F2DD8-8346-4731-A419-FF8E7C48DF97

3

5

APPENDIX 6 - PD / PD Genetic Schedule Years 6 - 13

PPMI

Version Date: 01Feb2021

Page 54 of 55


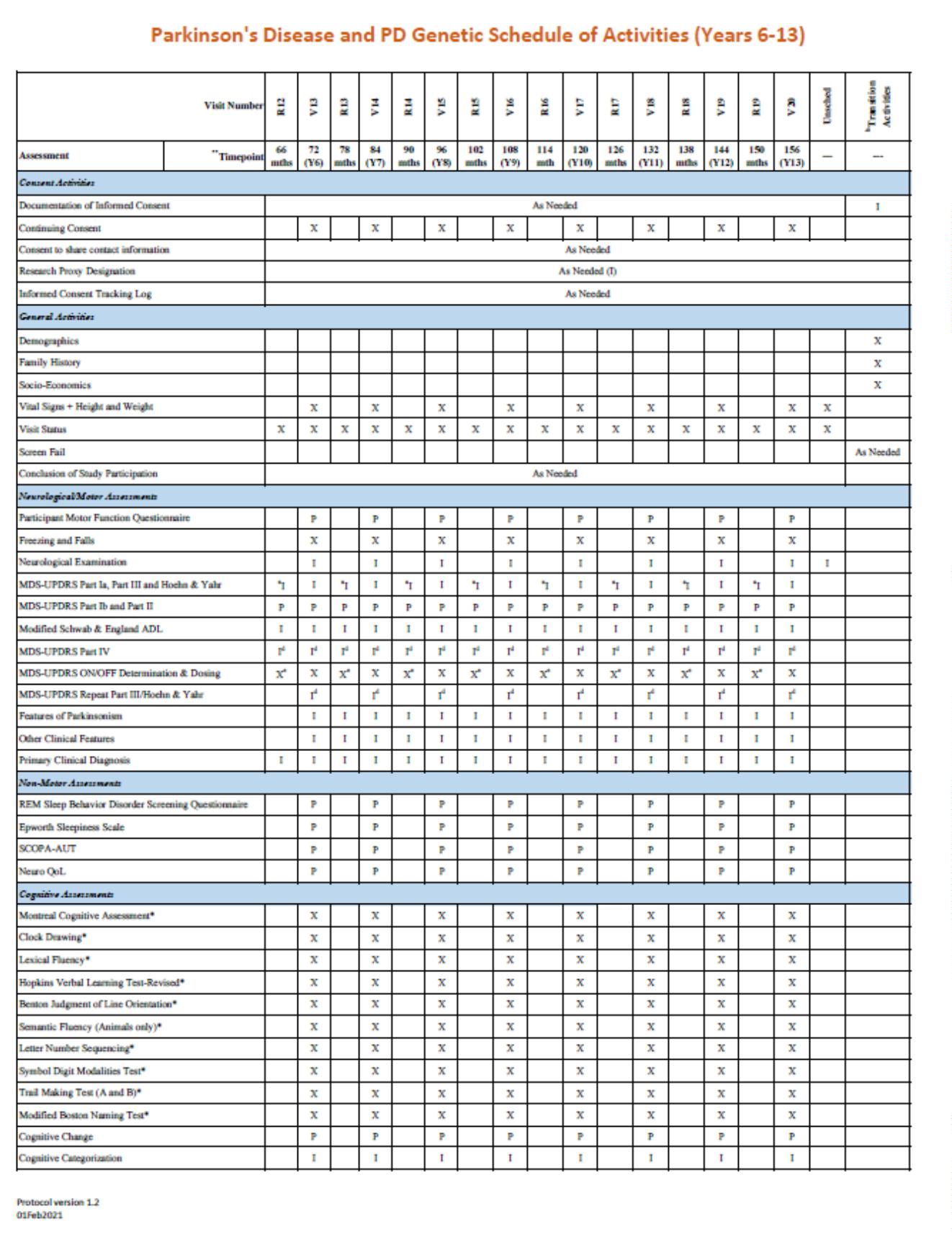

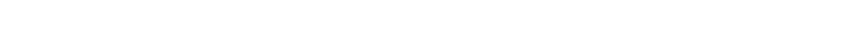


DocuSign Envelope ID: 706F2DD8-8346-4731-A419-FF8E7C48DF97

PPMI

Page 55 of 55

Version Date: 01Feb2021


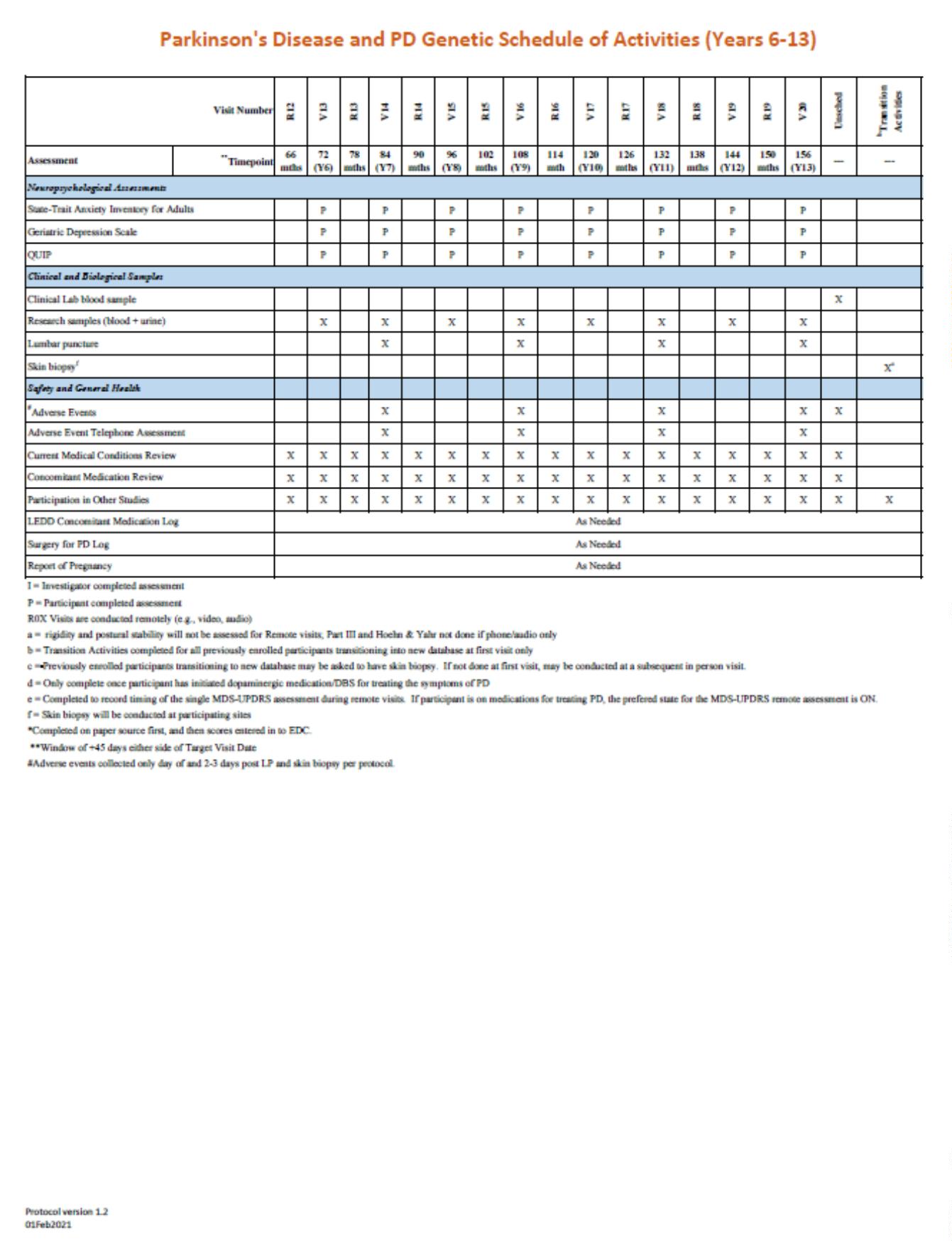

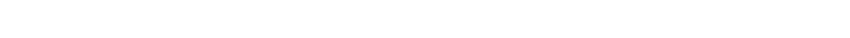

Supplement: Supplementary Materials — Supplementary Material 1: the detailed protocol of PPMI cohort. Supplementary Material 2: Q-Q plot and Manhattan plot of right putamen, right caudate, right anterior putamen, left putamen, left caudate, and left anterior putamen DaTscan EWAS. Supplementary Material 3: Q-Q plot and Manhattan plot of right putamen, right caudate, right anterior putamen, left putamen, left caudate, and left anterior putamen DaTscan GWAS. [file 2893662.f1.zip › Supplementary Material 1.docx]
