# Supplementary figures and images for: Genome- and Exome-Wide Association Studies Revealed Candidate Genes Associated with DaTscan Imaging Features
Source: Parkinsons Dis. 2023 Aug 23;2023:2893662. doi: 10.1155/2023/2893662 (PMC10468272; doi:10.1155/2023/2893662)

Manhattan plot of left anterior putamen DaTscan EWAS

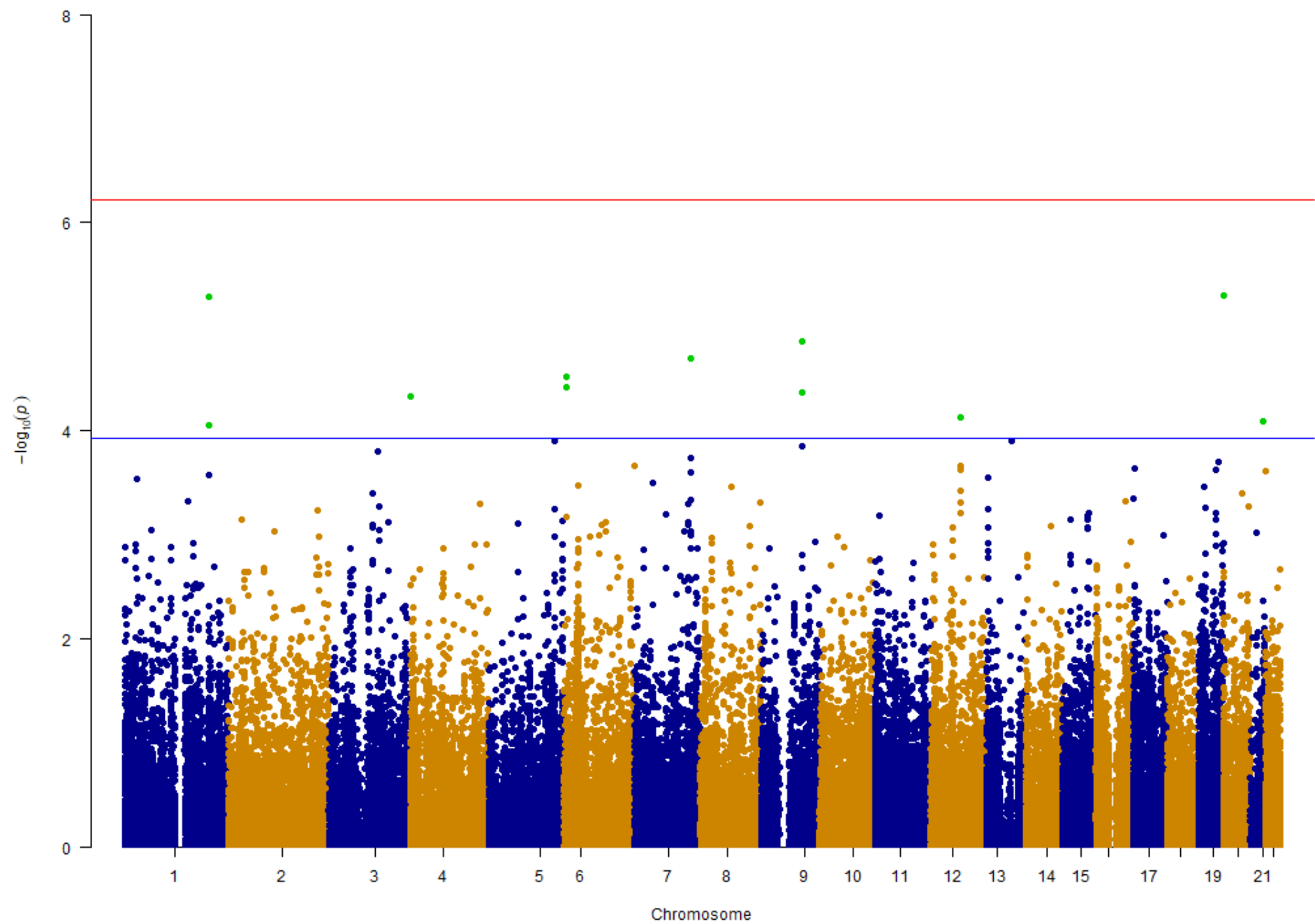

Supplement: Supplementary Materials — Supplementary Material 1: the detailed protocol of PPMI cohort. Supplementary Material 2: Q-Q plot and Manhattan plot of right putamen, right caudate, right anterior putamen, left putamen, left caudate, and left anterior putamen DaTscan EWAS. Supplementary Material 3: Q-Q plot and Manhattan plot of right putamen, right caudate, right anterior putamen, left putamen, left caudate, and left anterior putamen DaTscan GWAS. [file 2893662.f1.zip › Supplementary Material 2/LAP_manhattan_color.pdf]

Q-Q plot of left anterior putamen DaTscan EWAS

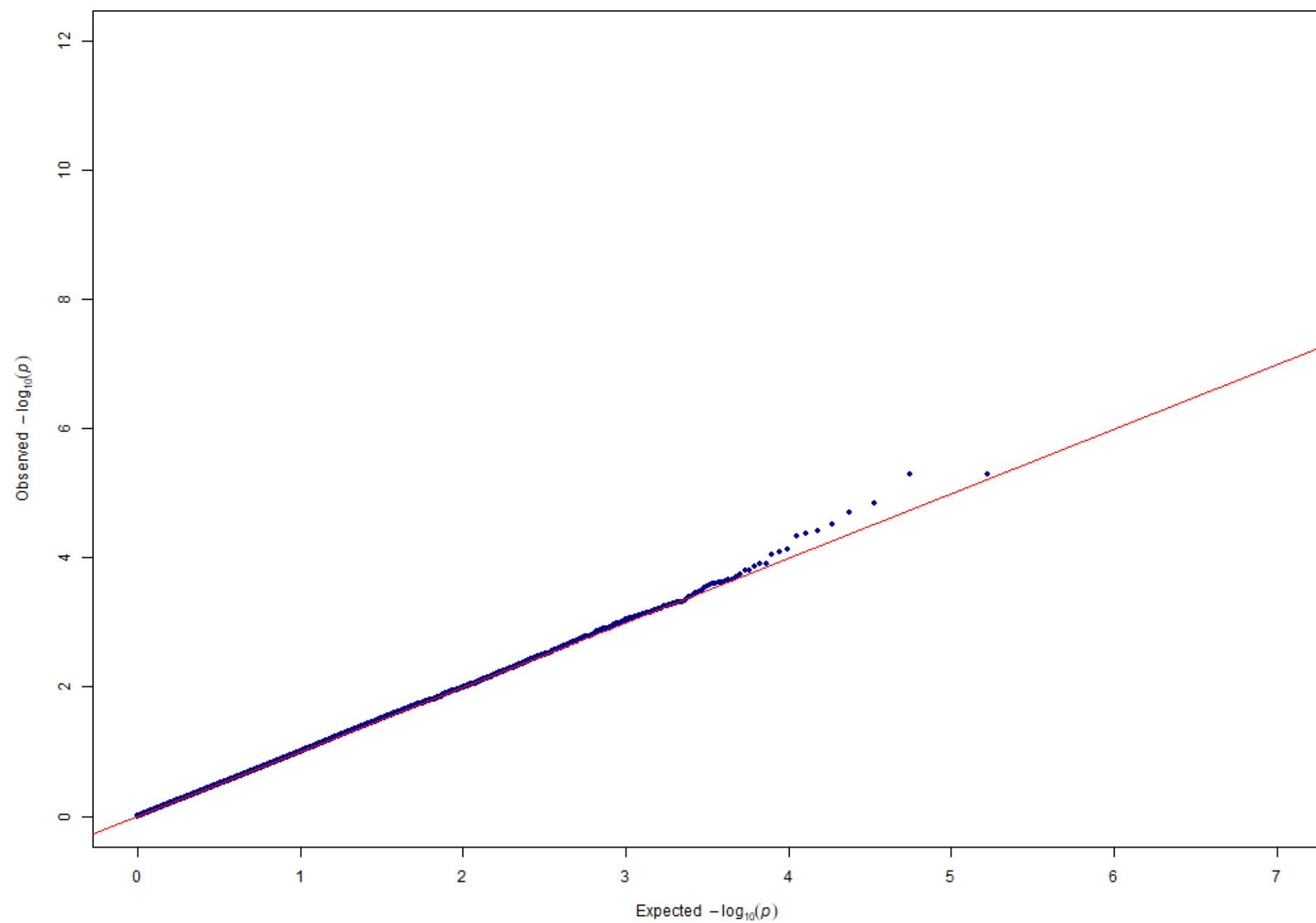

Supplement: Supplementary Materials — Supplementary Material 1: the detailed protocol of PPMI cohort. Supplementary Material 2: Q-Q plot and Manhattan plot of right putamen, right caudate, right anterior putamen, left putamen, left caudate, and left anterior putamen DaTscan EWAS. Supplementary Material 3: Q-Q plot and Manhattan plot of right putamen, right caudate, right anterior putamen, left putamen, left caudate, and left anterior putamen DaTscan GWAS. [file 2893662.f1.zip › Supplementary Material 2/LAP_qqplot_color.pdf]

Manhattan plot of left caudate DaTscan EWAS

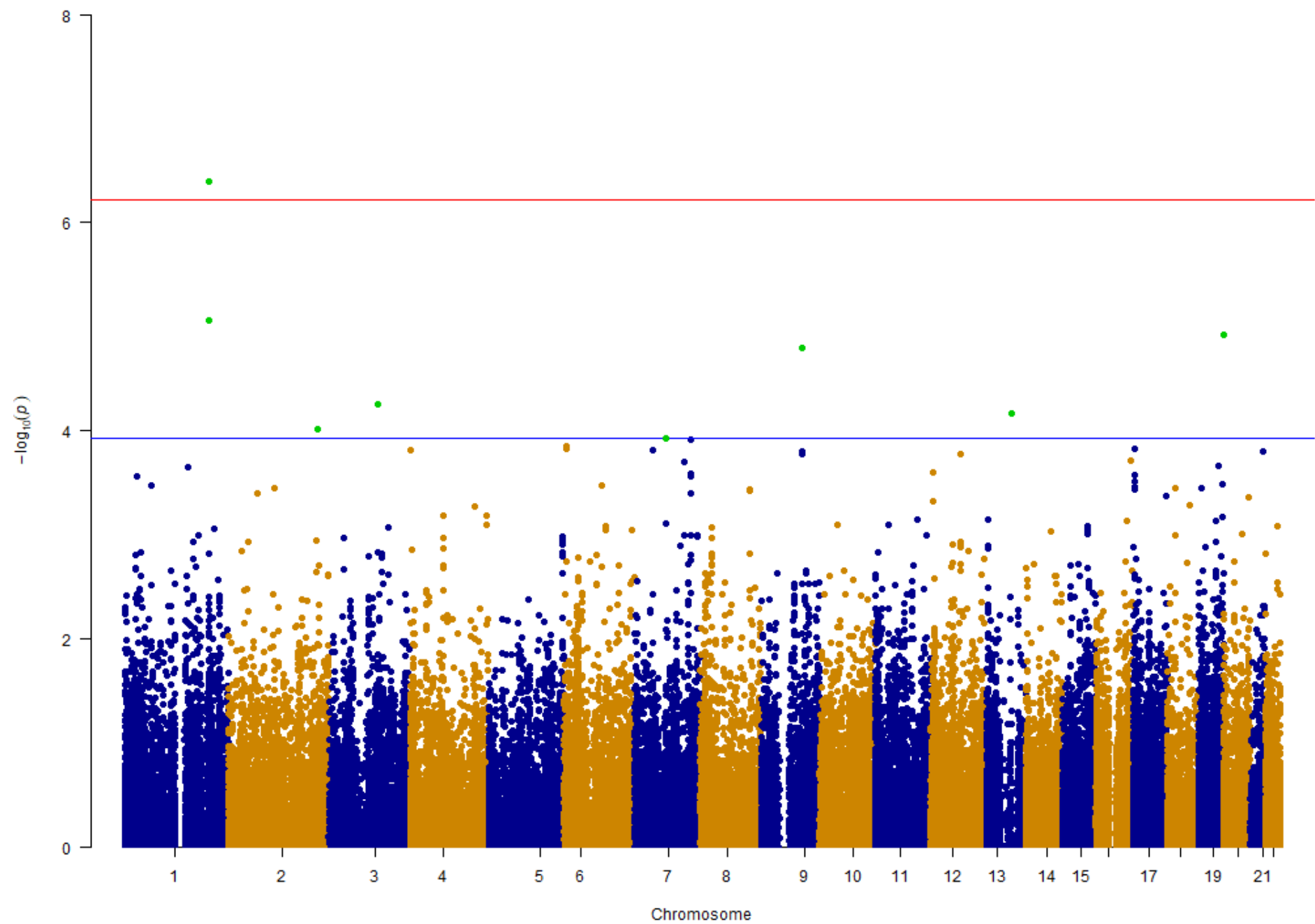

Supplement: Supplementary Materials — Supplementary Material 1: the detailed protocol of PPMI cohort. Supplementary Material 2: Q-Q plot and Manhattan plot of right putamen, right caudate, right anterior putamen, left putamen, left caudate, and left anterior putamen DaTscan EWAS. Supplementary Material 3: Q-Q plot and Manhattan plot of right putamen, right caudate, right anterior putamen, left putamen, left caudate, and left anterior putamen DaTscan GWAS. [file 2893662.f1.zip › Supplementary Material 2/LC_manhattan_color.pdf]

Q-Q plot of left caudate DaTscan EWAS

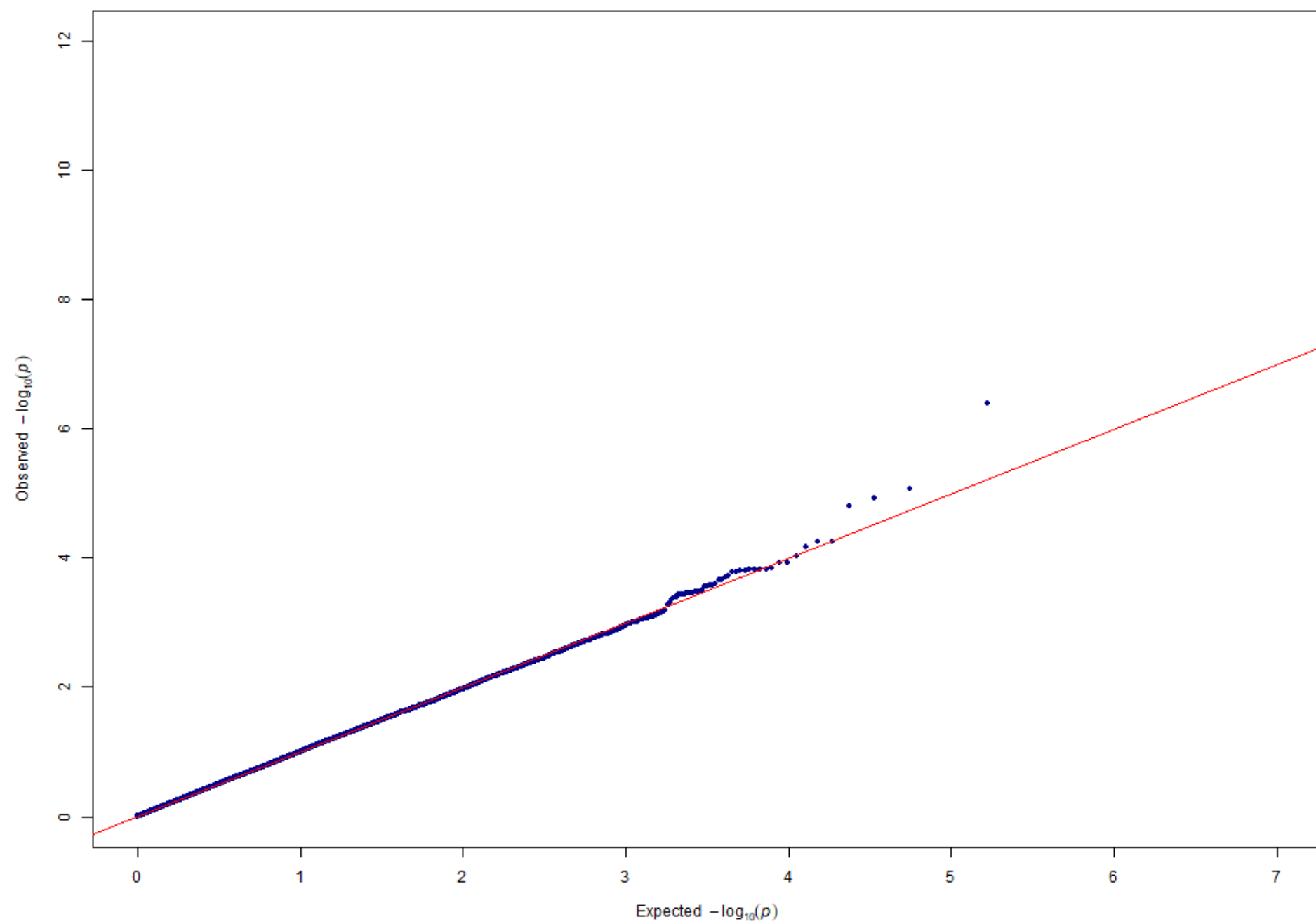

Supplement: Supplementary Materials — Supplementary Material 1: the detailed protocol of PPMI cohort. Supplementary Material 2: Q-Q plot and Manhattan plot of right putamen, right caudate, right anterior putamen, left putamen, left caudate, and left anterior putamen DaTscan EWAS. Supplementary Material 3: Q-Q plot and Manhattan plot of right putamen, right caudate, right anterior putamen, left putamen, left caudate, and left anterior putamen DaTscan GWAS. [file 2893662.f1.zip › Supplementary Material 2/LC_qqplot_color.pdf]

Manhattan plot of left putamen DaTscan EWAS

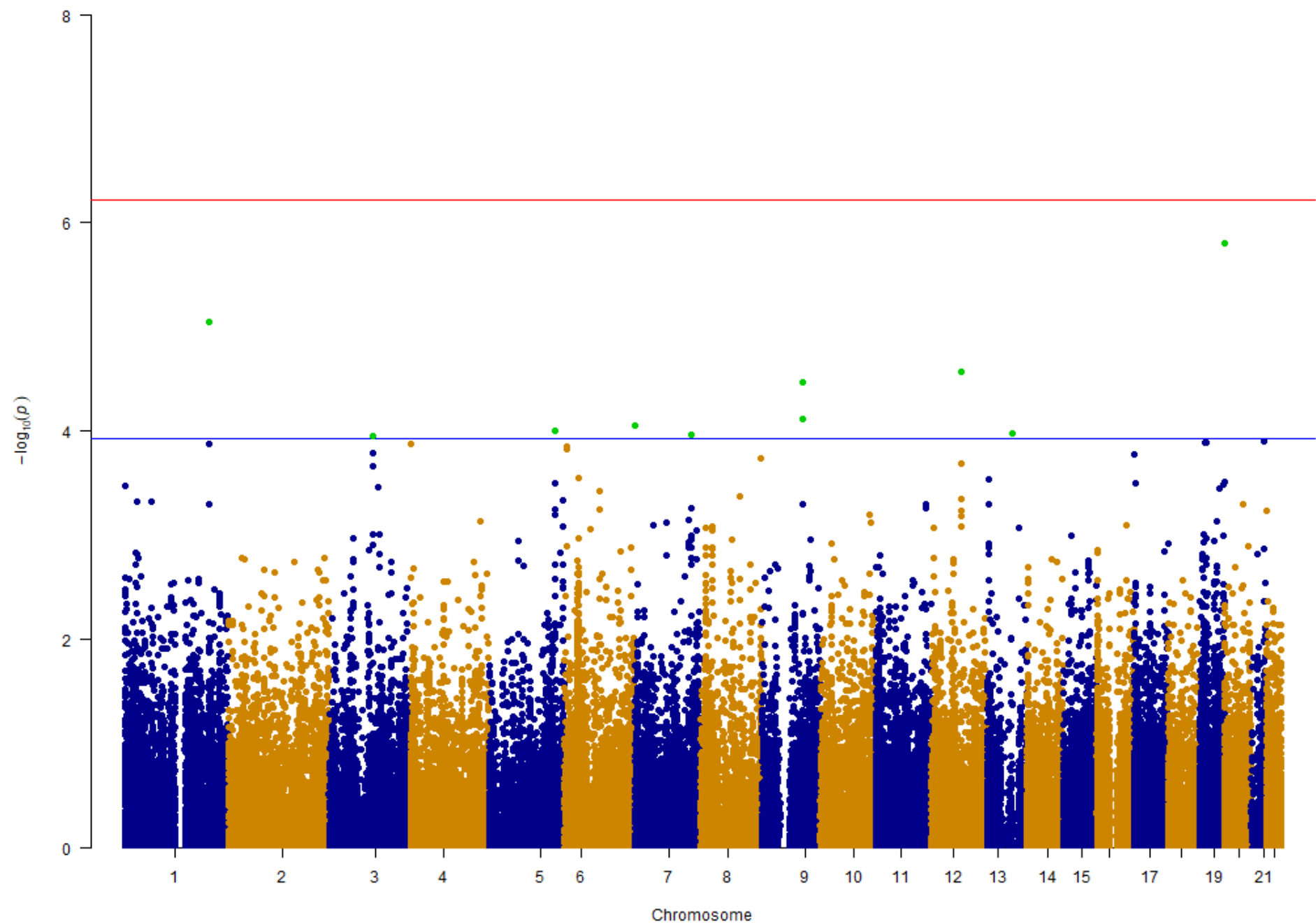

Supplement: Supplementary Materials — Supplementary Material 1: the detailed protocol of PPMI cohort. Supplementary Material 2: Q-Q plot and Manhattan plot of right putamen, right caudate, right anterior putamen, left putamen, left caudate, and left anterior putamen DaTscan EWAS. Supplementary Material 3: Q-Q plot and Manhattan plot of right putamen, right caudate, right anterior putamen, left putamen, left caudate, and left anterior putamen DaTscan GWAS. [file 2893662.f1.zip › Supplementary Material 2/LP_manhattan_color.pdf]

Q-Q plot of left putamen DaTscan EWAS

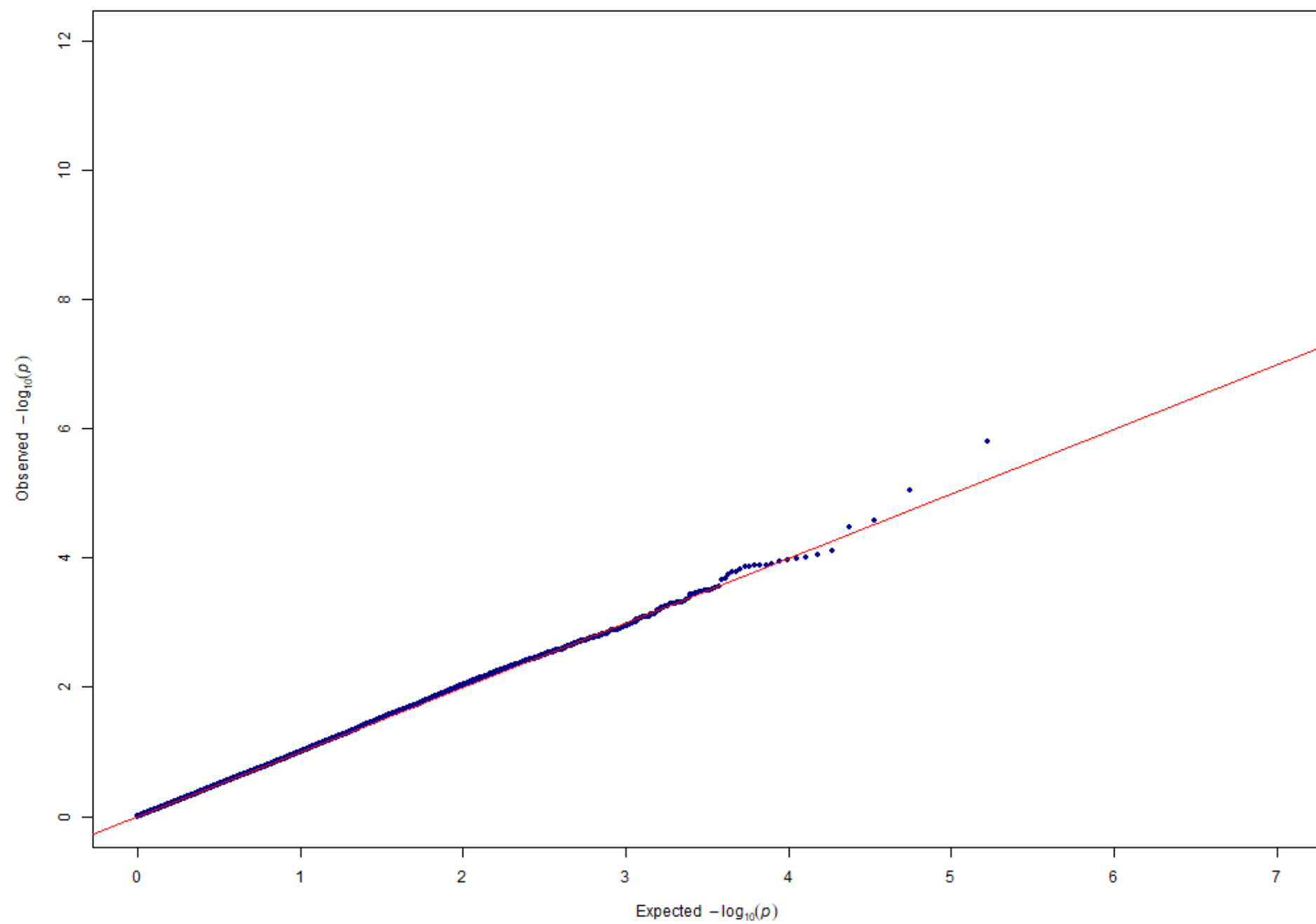

Supplement: Supplementary Materials — Supplementary Material 1: the detailed protocol of PPMI cohort. Supplementary Material 2: Q-Q plot and Manhattan plot of right putamen, right caudate, right anterior putamen, left putamen, left caudate, and left anterior putamen DaTscan EWAS. Supplementary Material 3: Q-Q plot and Manhattan plot of right putamen, right caudate, right anterior putamen, left putamen, left caudate, and left anterior putamen DaTscan GWAS. [file 2893662.f1.zip › Supplementary Material 2/LP_qqplot_color.pdf]

Manhattan plot of right anterior putamen DaTscan EWAS

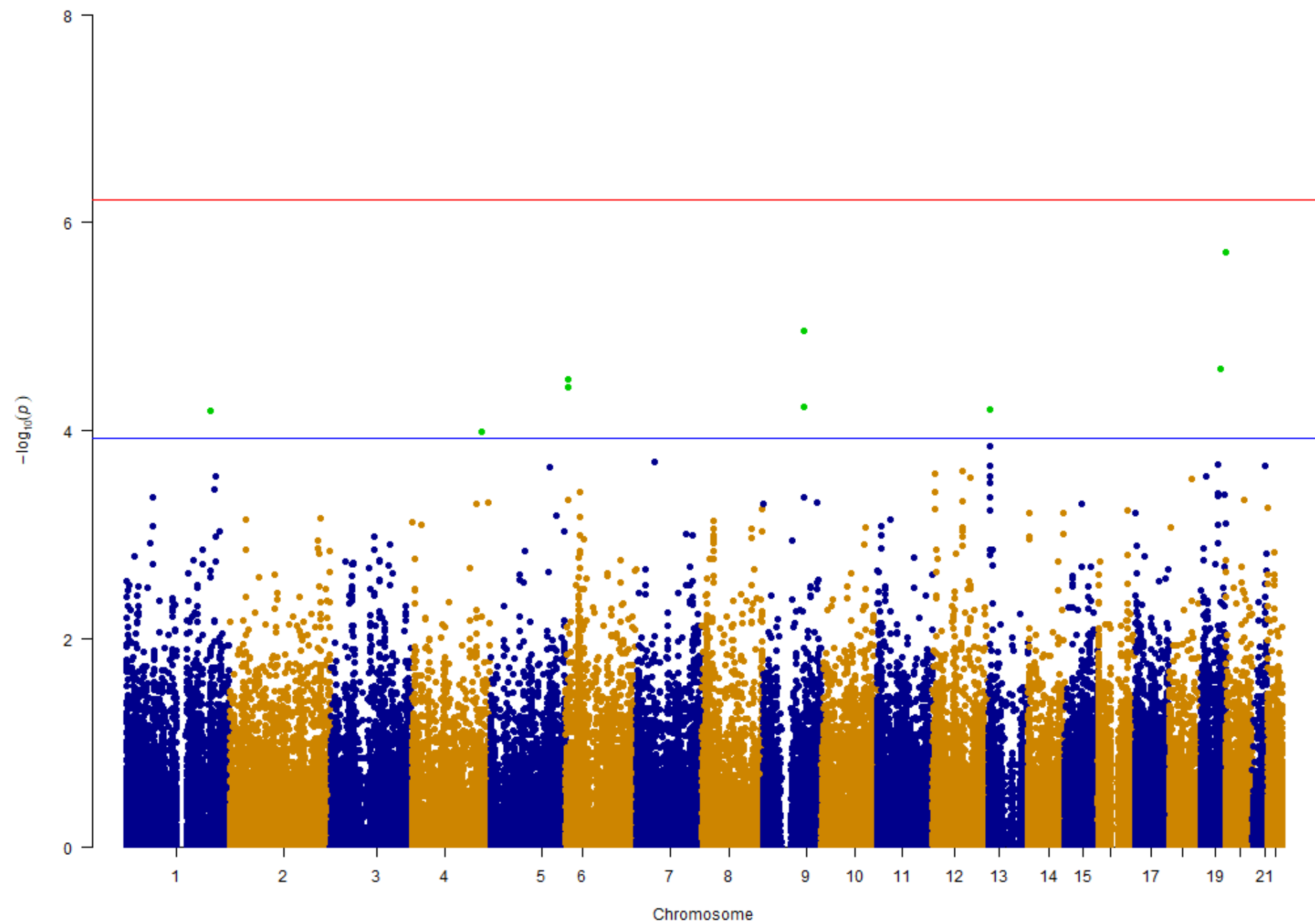

Supplement: Supplementary Materials — Supplementary Material 1: the detailed protocol of PPMI cohort. Supplementary Material 2: Q-Q plot and Manhattan plot of right putamen, right caudate, right anterior putamen, left putamen, left caudate, and left anterior putamen DaTscan EWAS. Supplementary Material 3: Q-Q plot and Manhattan plot of right putamen, right caudate, right anterior putamen, left putamen, left caudate, and left anterior putamen DaTscan GWAS. [file 2893662.f1.zip › Supplementary Material 2/RAP_manhattan_color.pdf]

Q-Q plot of right anterior putamen DaTscan EWAS

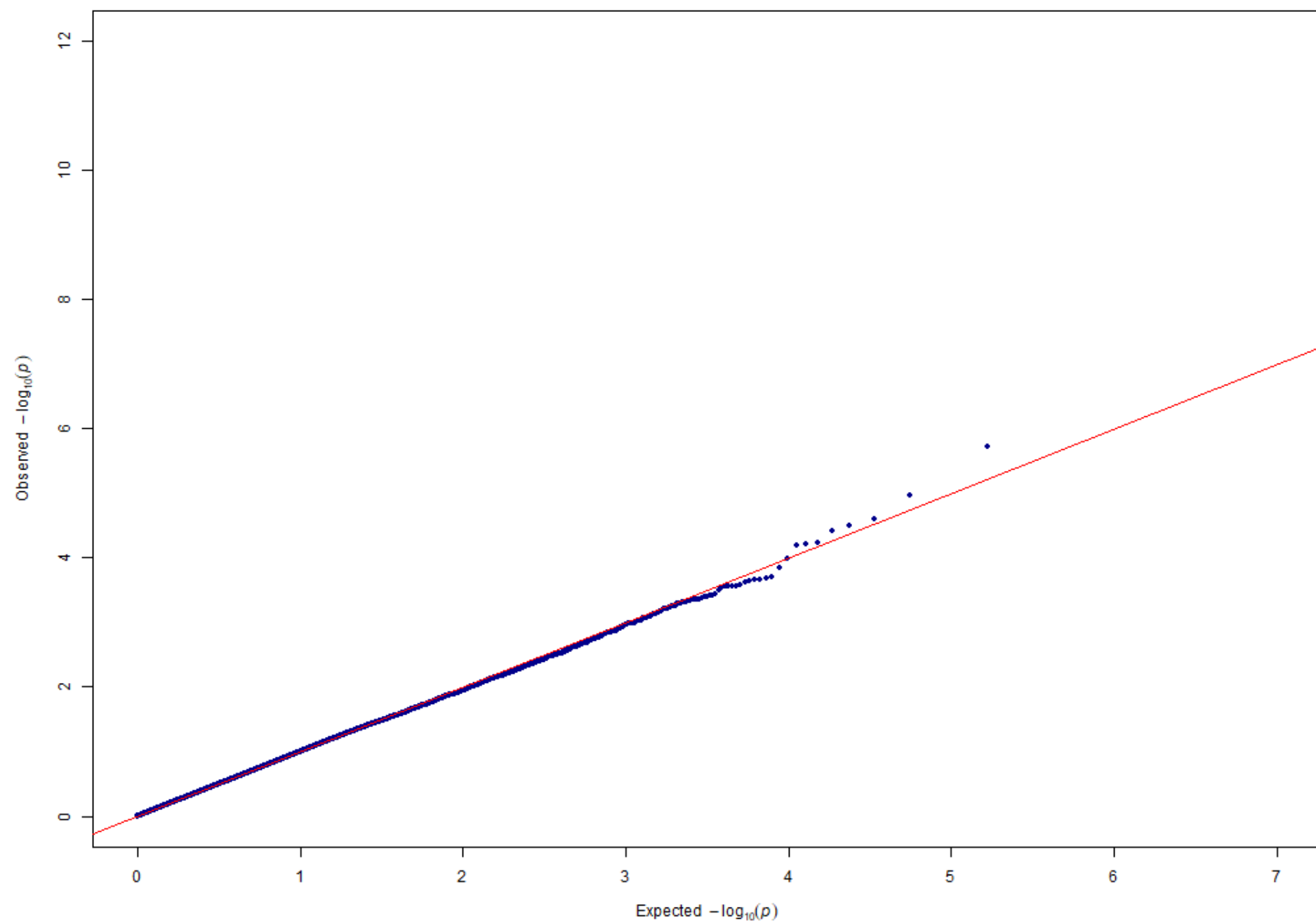

Supplement: Supplementary Materials — Supplementary Material 1: the detailed protocol of PPMI cohort. Supplementary Material 2: Q-Q plot and Manhattan plot of right putamen, right caudate, right anterior putamen, left putamen, left caudate, and left anterior putamen DaTscan EWAS. Supplementary Material 3: Q-Q plot and Manhattan plot of right putamen, right caudate, right anterior putamen, left putamen, left caudate, and left anterior putamen DaTscan GWAS. [file 2893662.f1.zip › Supplementary Material 2/RAP_qqplot_color.pdf]

Manhattan plot of right caudate DaTscan EWAS

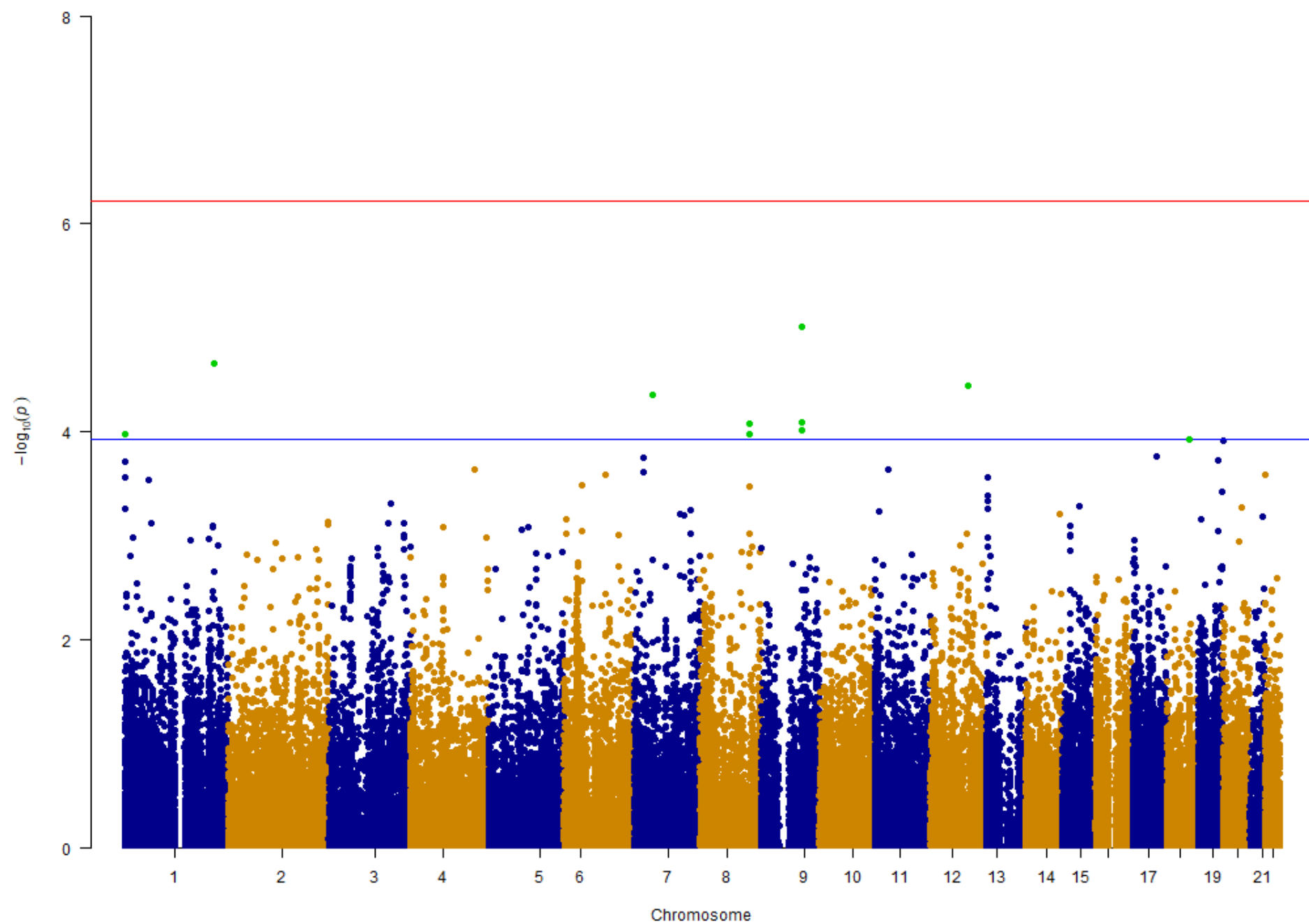

Supplement: Supplementary Materials — Supplementary Material 1: the detailed protocol of PPMI cohort. Supplementary Material 2: Q-Q plot and Manhattan plot of right putamen, right caudate, right anterior putamen, left putamen, left caudate, and left anterior putamen DaTscan EWAS. Supplementary Material 3: Q-Q plot and Manhattan plot of right putamen, right caudate, right anterior putamen, left putamen, left caudate, and left anterior putamen DaTscan GWAS. [file 2893662.f1.zip › Supplementary Material 2/RC_manhattan_color.pdf]

Q-Q plot of right caudate DaTscan EWAS

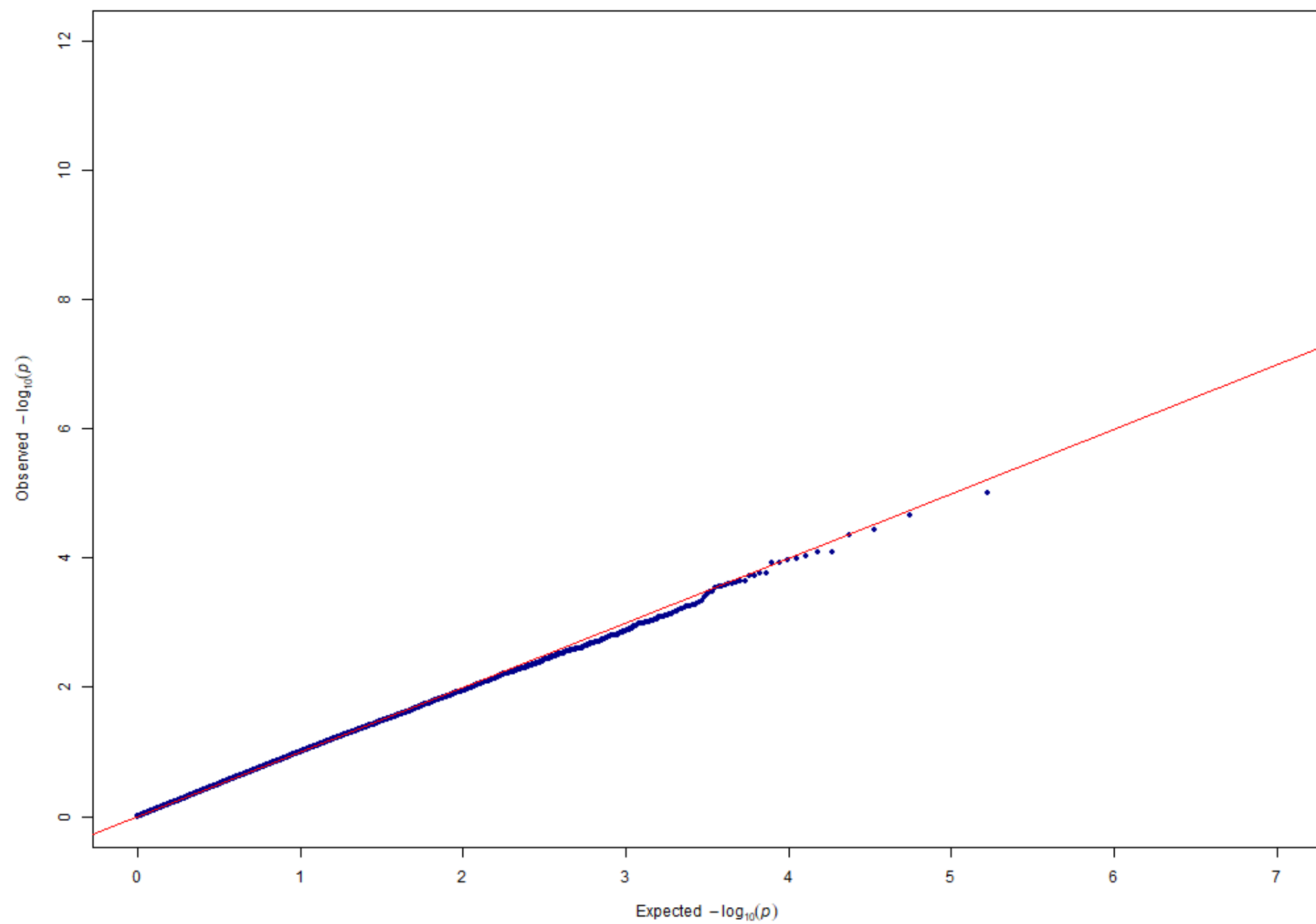

Supplement: Supplementary Materials — Supplementary Material 1: the detailed protocol of PPMI cohort. Supplementary Material 2: Q-Q plot and Manhattan plot of right putamen, right caudate, right anterior putamen, left putamen, left caudate, and left anterior putamen DaTscan EWAS. Supplementary Material 3: Q-Q plot and Manhattan plot of right putamen, right caudate, right anterior putamen, left putamen, left caudate, and left anterior putamen DaTscan GWAS. [file 2893662.f1.zip › Supplementary Material 2/RC_qqplot_color.pdf]

Manhattan plot of right putamen DaTscan EWAS

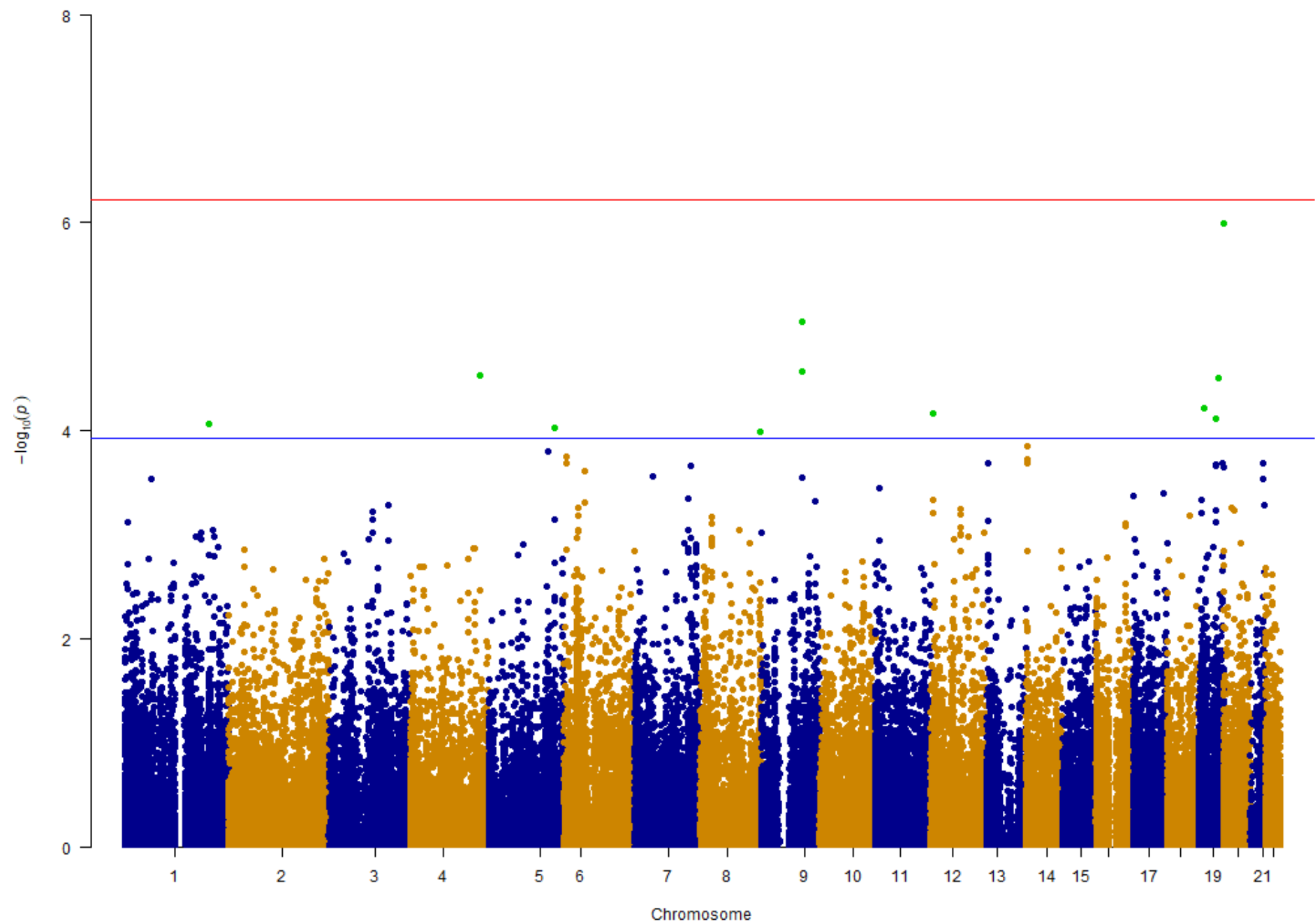

Supplement: Supplementary Materials — Supplementary Material 1: the detailed protocol of PPMI cohort. Supplementary Material 2: Q-Q plot and Manhattan plot of right putamen, right caudate, right anterior putamen, left putamen, left caudate, and left anterior putamen DaTscan EWAS. Supplementary Material 3: Q-Q plot and Manhattan plot of right putamen, right caudate, right anterior putamen, left putamen, left caudate, and left anterior putamen DaTscan GWAS. [file 2893662.f1.zip › Supplementary Material 2/RP_manhattan_color.pdf]

Q-Q plot of right putamen DaTscan EWAS

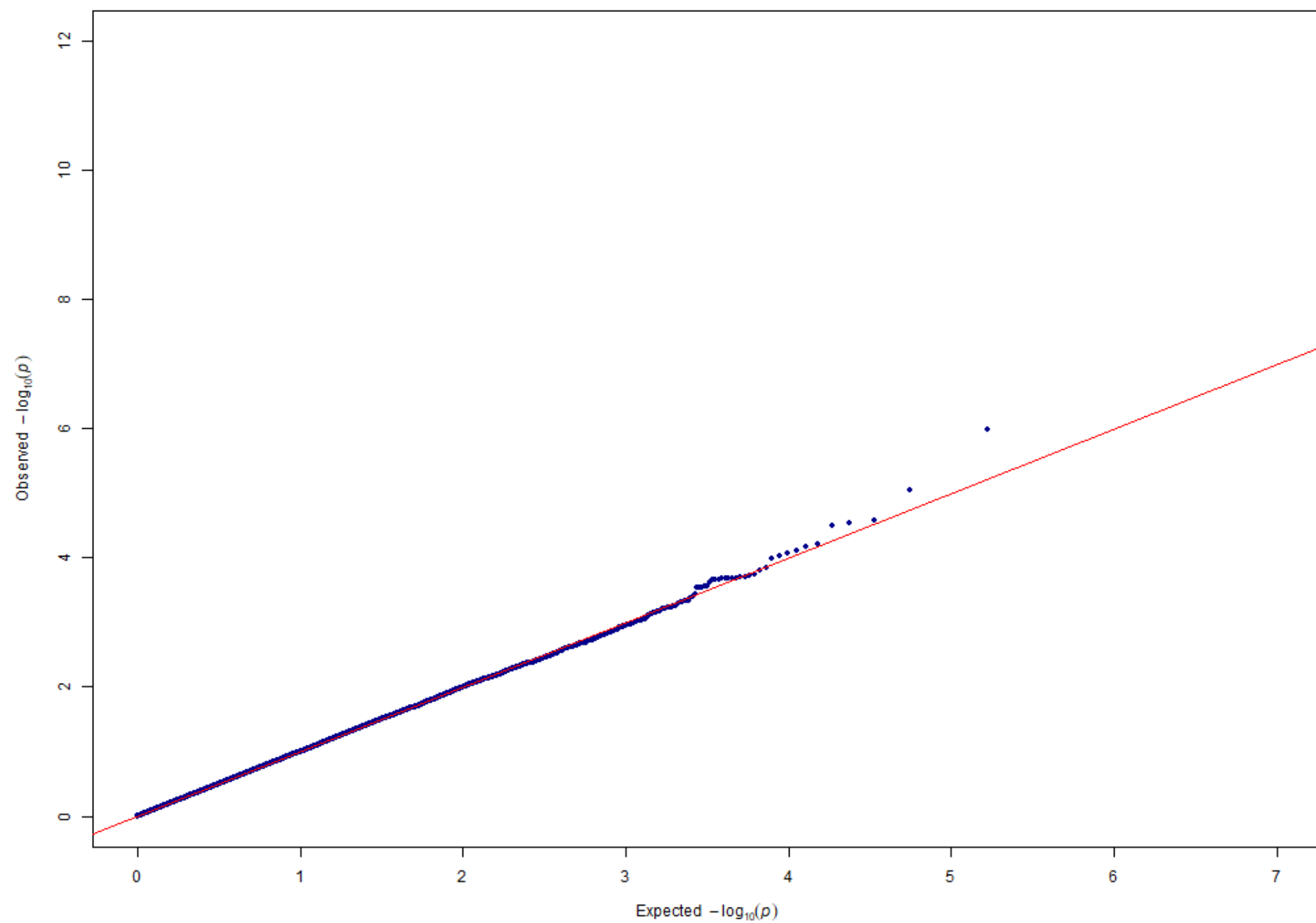

Supplement: Supplementary Materials — Supplementary Material 1: the detailed protocol of PPMI cohort. Supplementary Material 2: Q-Q plot and Manhattan plot of right putamen, right caudate, right anterior putamen, left putamen, left caudate, and left anterior putamen DaTscan EWAS. Supplementary Material 3: Q-Q plot and Manhattan plot of right putamen, right caudate, right anterior putamen, left putamen, left caudate, and left anterior putamen DaTscan GWAS. [file 2893662.f1.zip › Supplementary Material 2/RP_qqplot_color.pdf]

Manhattan plot of left anterior putamen DaTscan GWAS

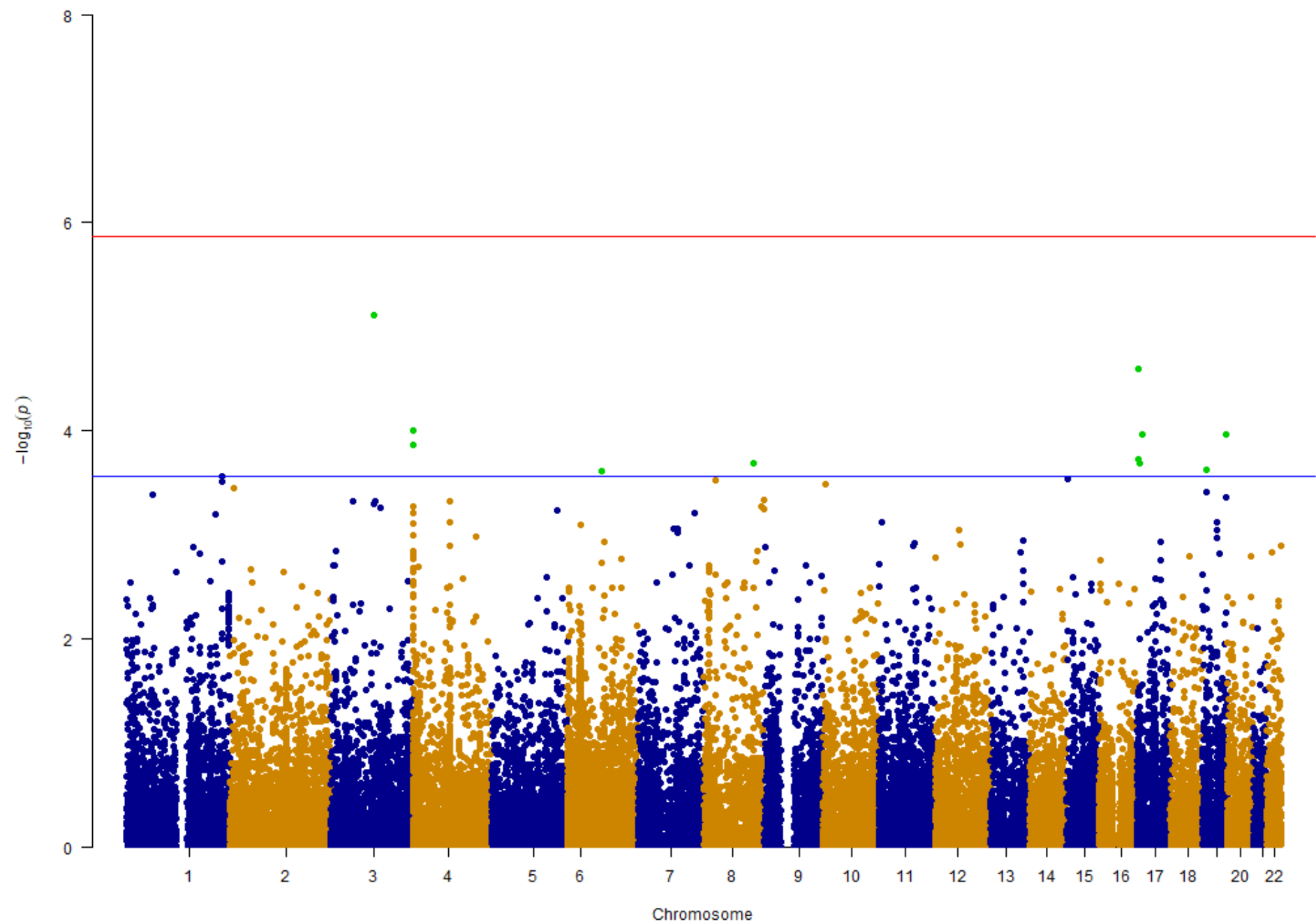

Supplement: Supplementary Materials — Supplementary Material 1: the detailed protocol of PPMI cohort. Supplementary Material 2: Q-Q plot and Manhattan plot of right putamen, right caudate, right anterior putamen, left putamen, left caudate, and left anterior putamen DaTscan EWAS. Supplementary Material 3: Q-Q plot and Manhattan plot of right putamen, right caudate, right anterior putamen, left putamen, left caudate, and left anterior putamen DaTscan GWAS. [file 2893662.f1.zip › Supplementary Material 3/LAP_manhattan_color.pdf]

Q-Q plot of left anterior putamen DaTscan GWAS

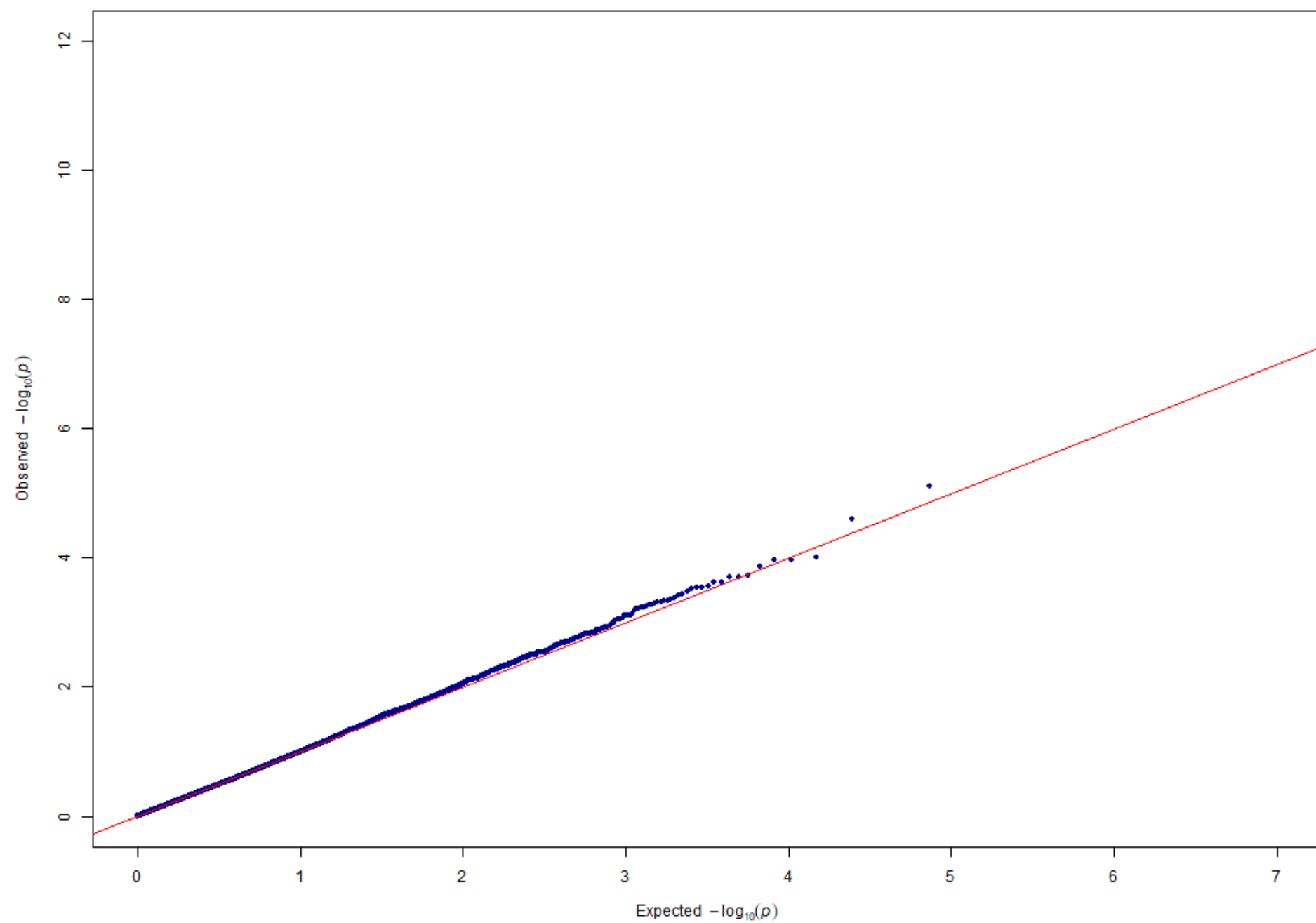

Supplement: Supplementary Materials — Supplementary Material 1: the detailed protocol of PPMI cohort. Supplementary Material 2: Q-Q plot and Manhattan plot of right putamen, right caudate, right anterior putamen, left putamen, left caudate, and left anterior putamen DaTscan EWAS. Supplementary Material 3: Q-Q plot and Manhattan plot of right putamen, right caudate, right anterior putamen, left putamen, left caudate, and left anterior putamen DaTscan GWAS. [file 2893662.f1.zip › Supplementary Material 3/LAP_qq_color.pdf]

Manhattan plot of left caudate DaTscan GWAS

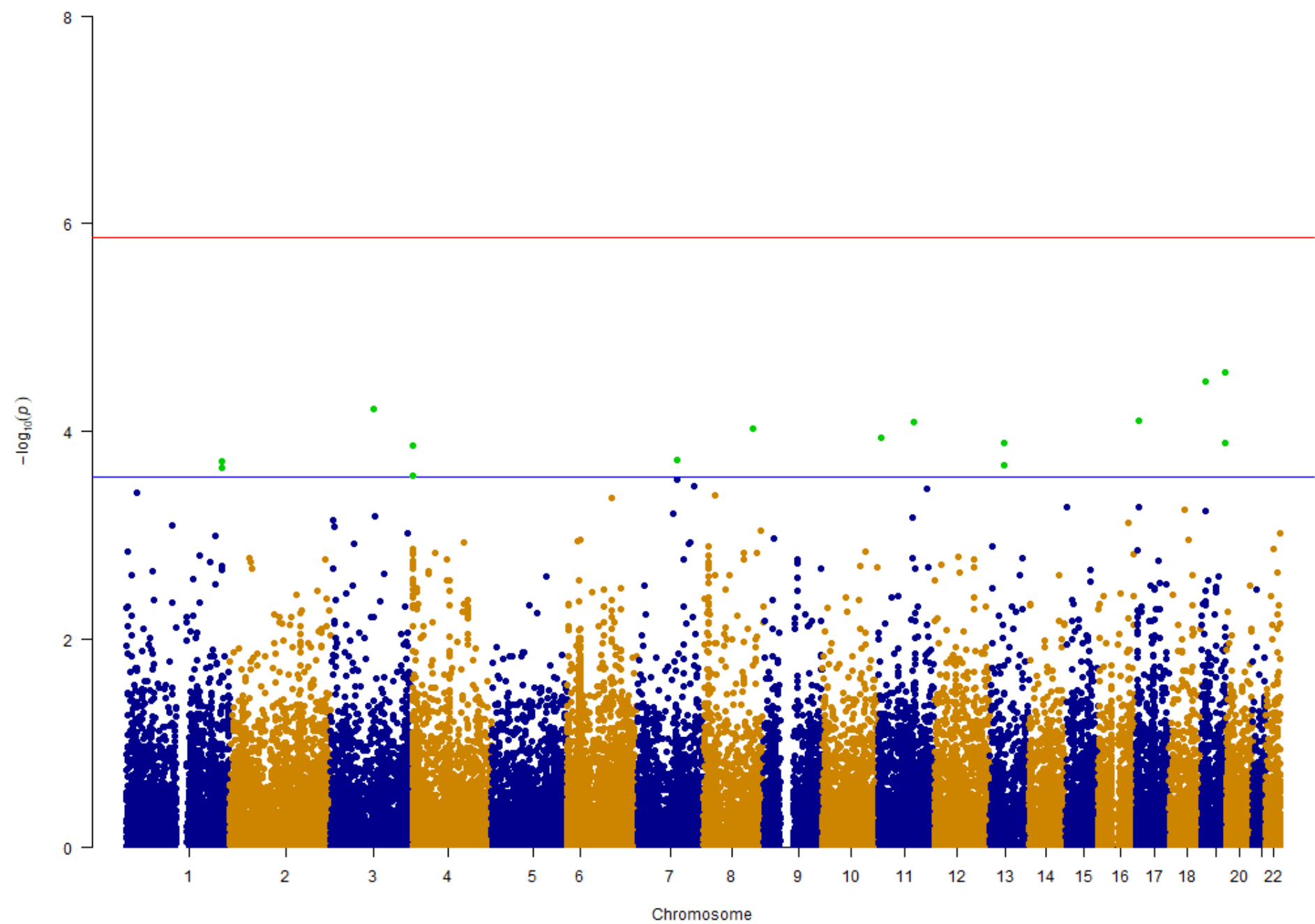

Supplement: Supplementary Materials — Supplementary Material 1: the detailed protocol of PPMI cohort. Supplementary Material 2: Q-Q plot and Manhattan plot of right putamen, right caudate, right anterior putamen, left putamen, left caudate, and left anterior putamen DaTscan EWAS. Supplementary Material 3: Q-Q plot and Manhattan plot of right putamen, right caudate, right anterior putamen, left putamen, left caudate, and left anterior putamen DaTscan GWAS. [file 2893662.f1.zip › Supplementary Material 3/LC_manhattan_color.pdf]

Q-Q plot of left caudate DaTscan GWAS

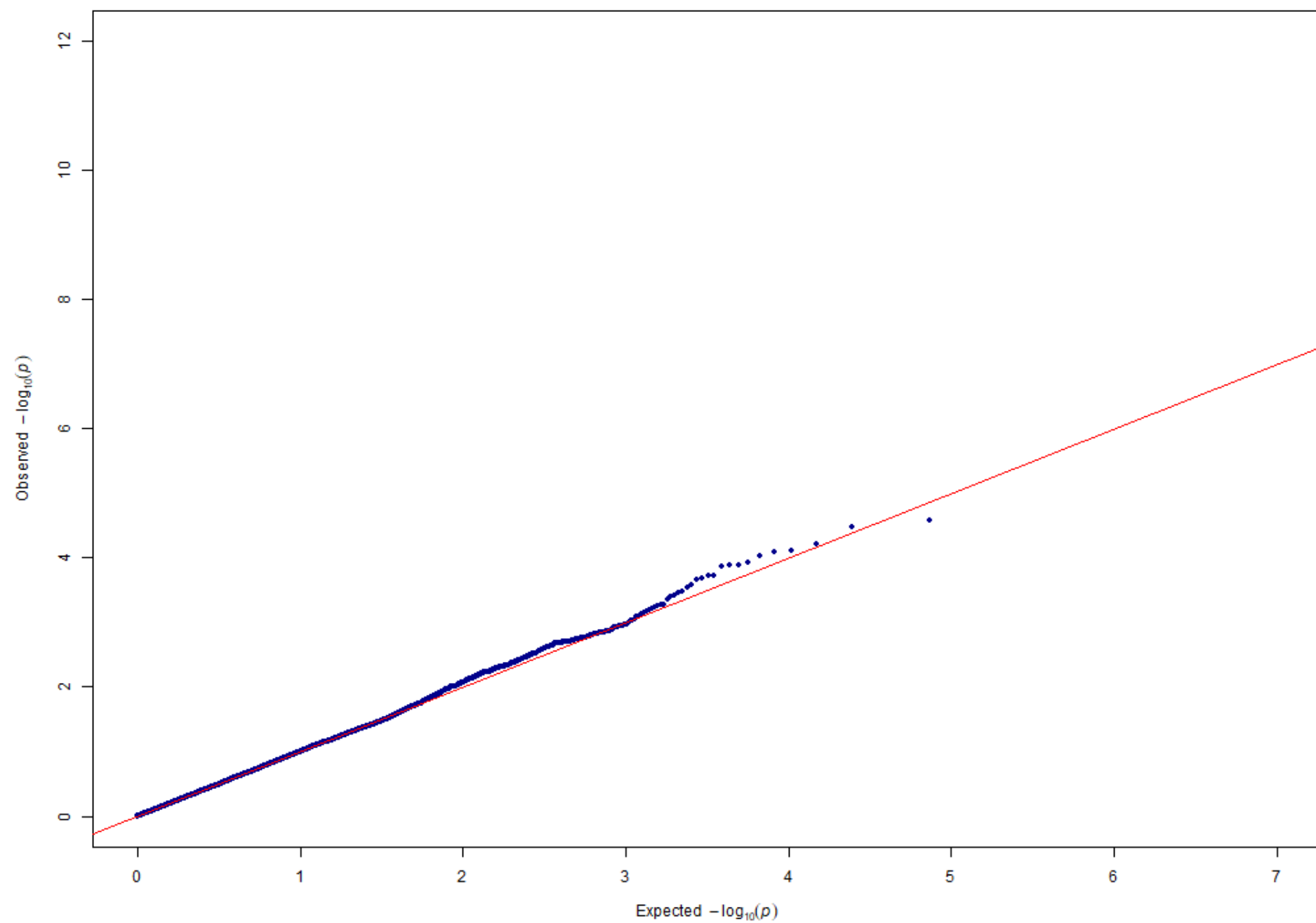

Supplement: Supplementary Materials — Supplementary Material 1: the detailed protocol of PPMI cohort. Supplementary Material 2: Q-Q plot and Manhattan plot of right putamen, right caudate, right anterior putamen, left putamen, left caudate, and left anterior putamen DaTscan EWAS. Supplementary Material 3: Q-Q plot and Manhattan plot of right putamen, right caudate, right anterior putamen, left putamen, left caudate, and left anterior putamen DaTscan GWAS. [file 2893662.f1.zip › Supplementary Material 3/LC_qq_color.pdf]

Manhattan plot of left putamen DaTscan GWAS

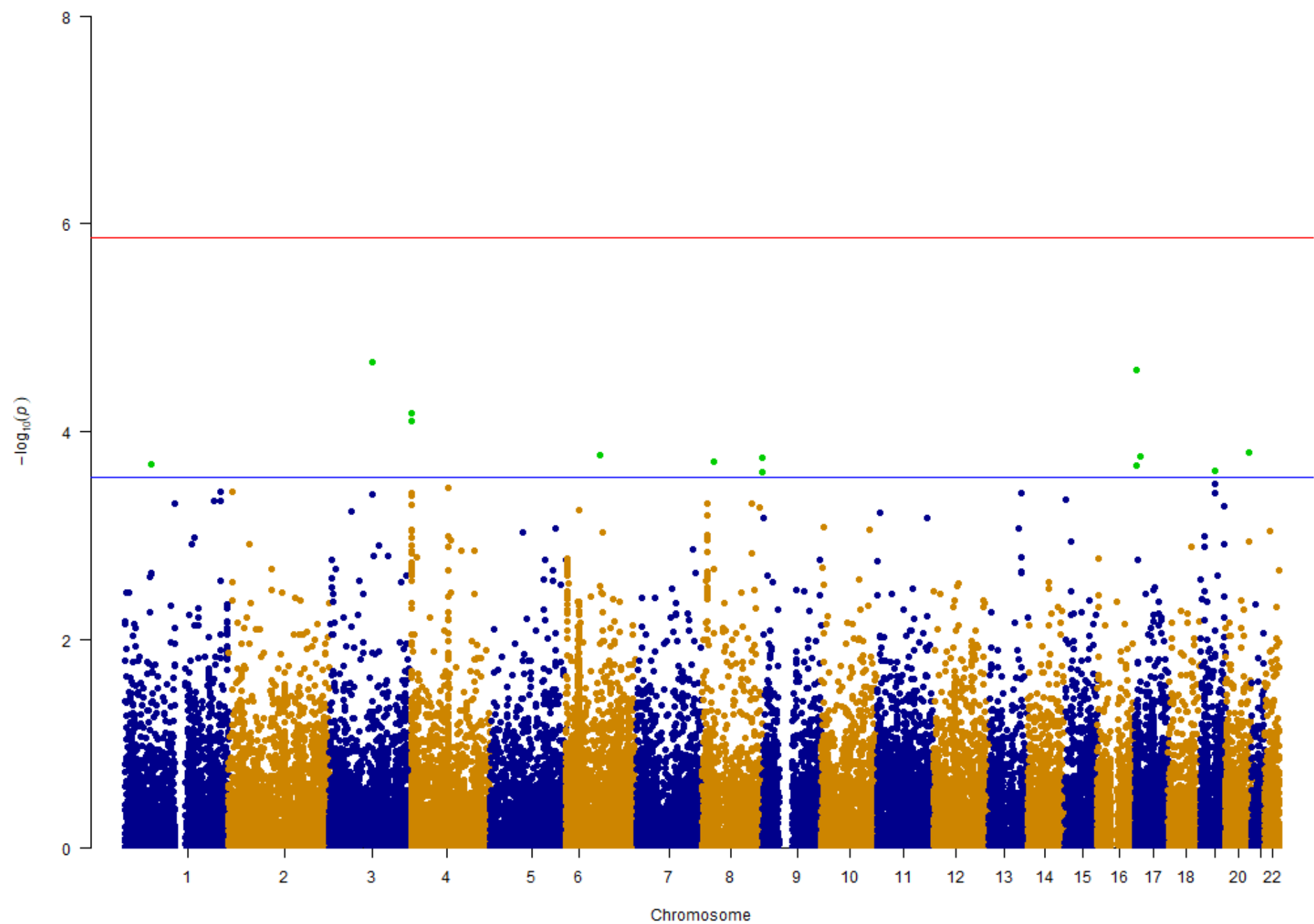

Supplement: Supplementary Materials — Supplementary Material 1: the detailed protocol of PPMI cohort. Supplementary Material 2: Q-Q plot and Manhattan plot of right putamen, right caudate, right anterior putamen, left putamen, left caudate, and left anterior putamen DaTscan EWAS. Supplementary Material 3: Q-Q plot and Manhattan plot of right putamen, right caudate, right anterior putamen, left putamen, left caudate, and left anterior putamen DaTscan GWAS. [file 2893662.f1.zip › Supplementary Material 3/LP_manhattan_color.pdf]

Q-Q plot of left putamen DaTscan GWAS

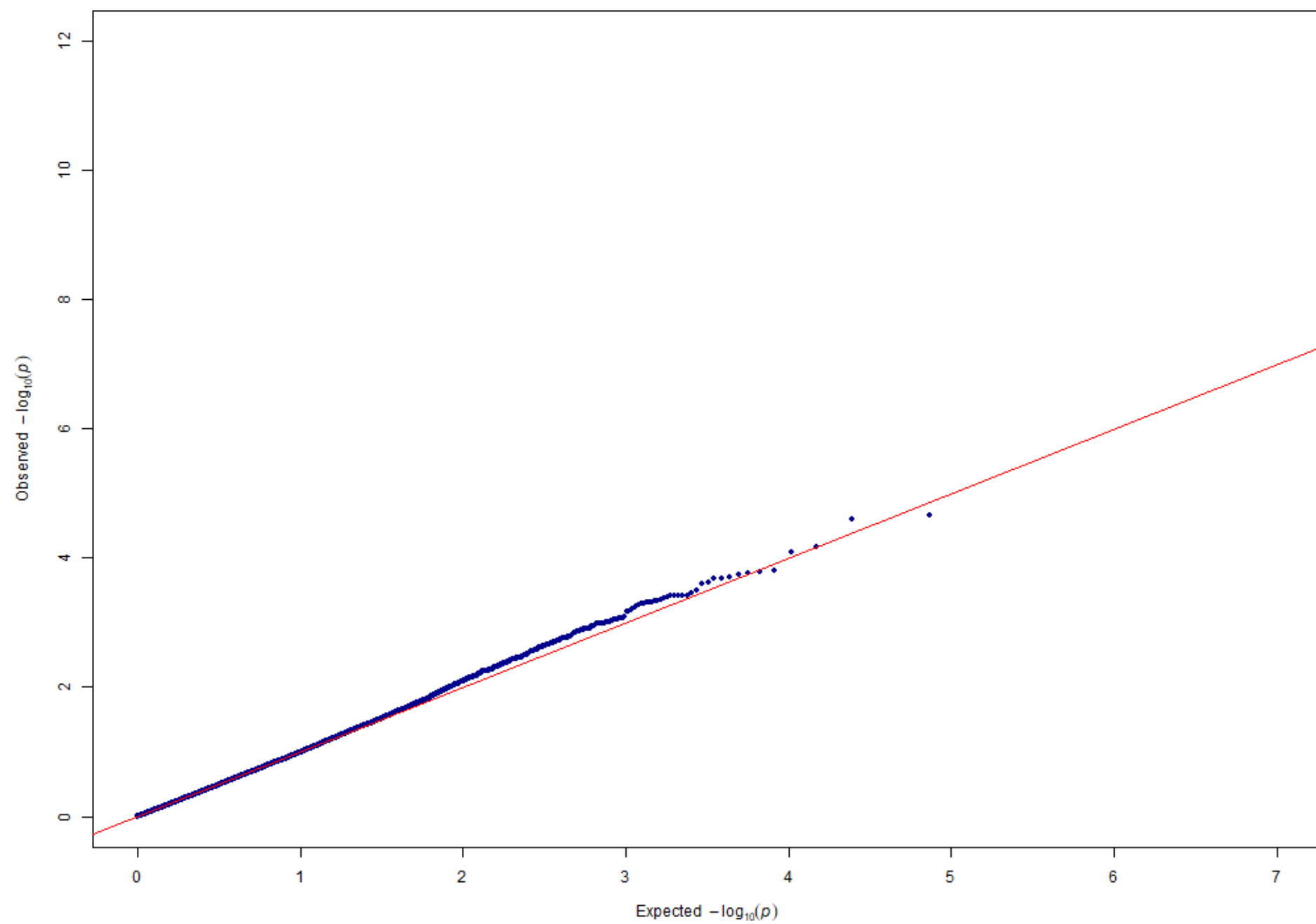

Supplement: Supplementary Materials — Supplementary Material 1: the detailed protocol of PPMI cohort. Supplementary Material 2: Q-Q plot and Manhattan plot of right putamen, right caudate, right anterior putamen, left putamen, left caudate, and left anterior putamen DaTscan EWAS. Supplementary Material 3: Q-Q plot and Manhattan plot of right putamen, right caudate, right anterior putamen, left putamen, left caudate, and left anterior putamen DaTscan GWAS. [file 2893662.f1.zip › Supplementary Material 3/LP_qq_color.pdf]

Manhattan plot of right anterior putamen DaTscan GWAS

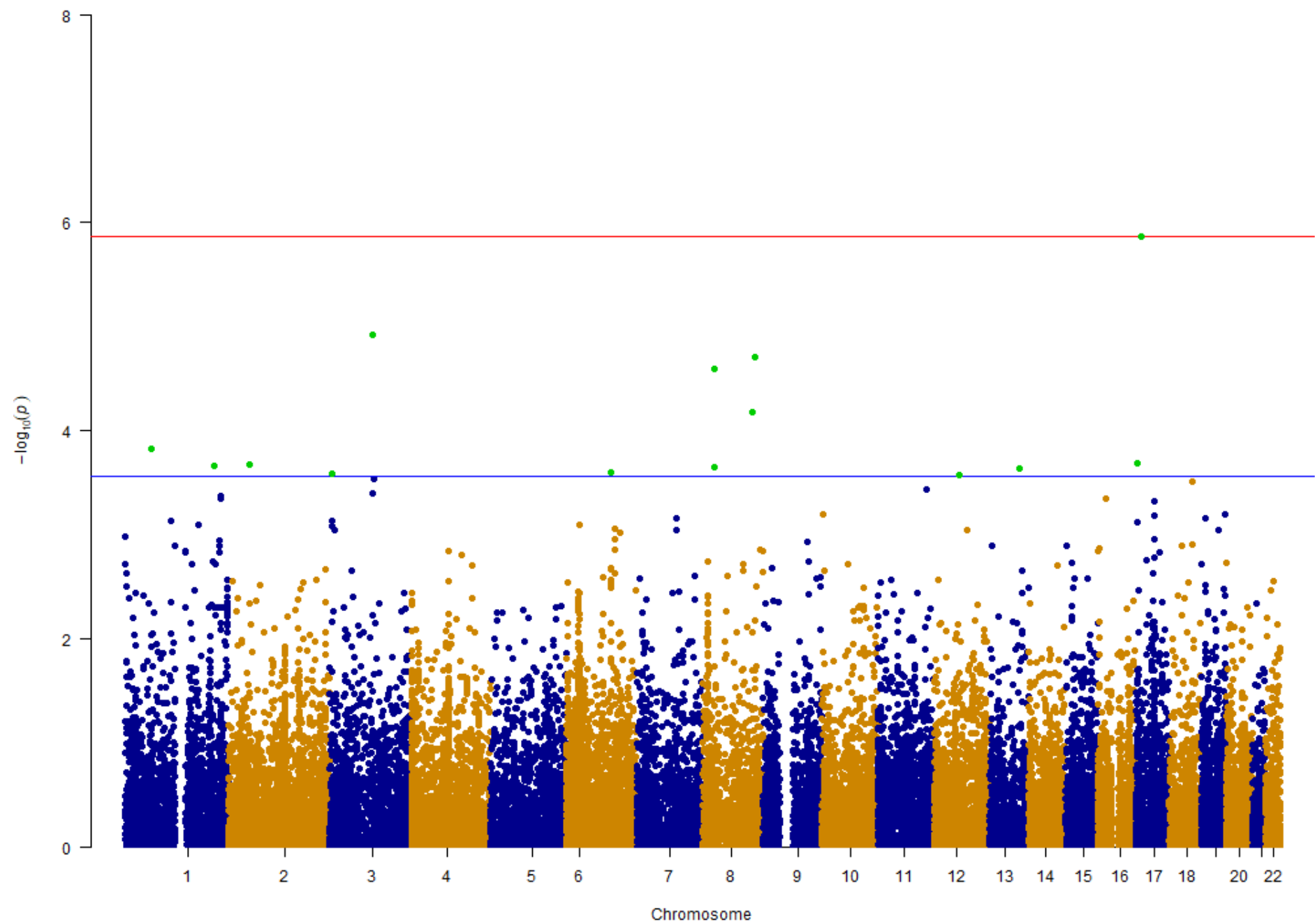

Supplement: Supplementary Materials — Supplementary Material 1: the detailed protocol of PPMI cohort. Supplementary Material 2: Q-Q plot and Manhattan plot of right putamen, right caudate, right anterior putamen, left putamen, left caudate, and left anterior putamen DaTscan EWAS. Supplementary Material 3: Q-Q plot and Manhattan plot of right putamen, right caudate, right anterior putamen, left putamen, left caudate, and left anterior putamen DaTscan GWAS. [file 2893662.f1.zip › Supplementary Material 3/RAP_manhattan_color.pdf]

Q-Q plot of right anterior putamen DaTscan GWAS

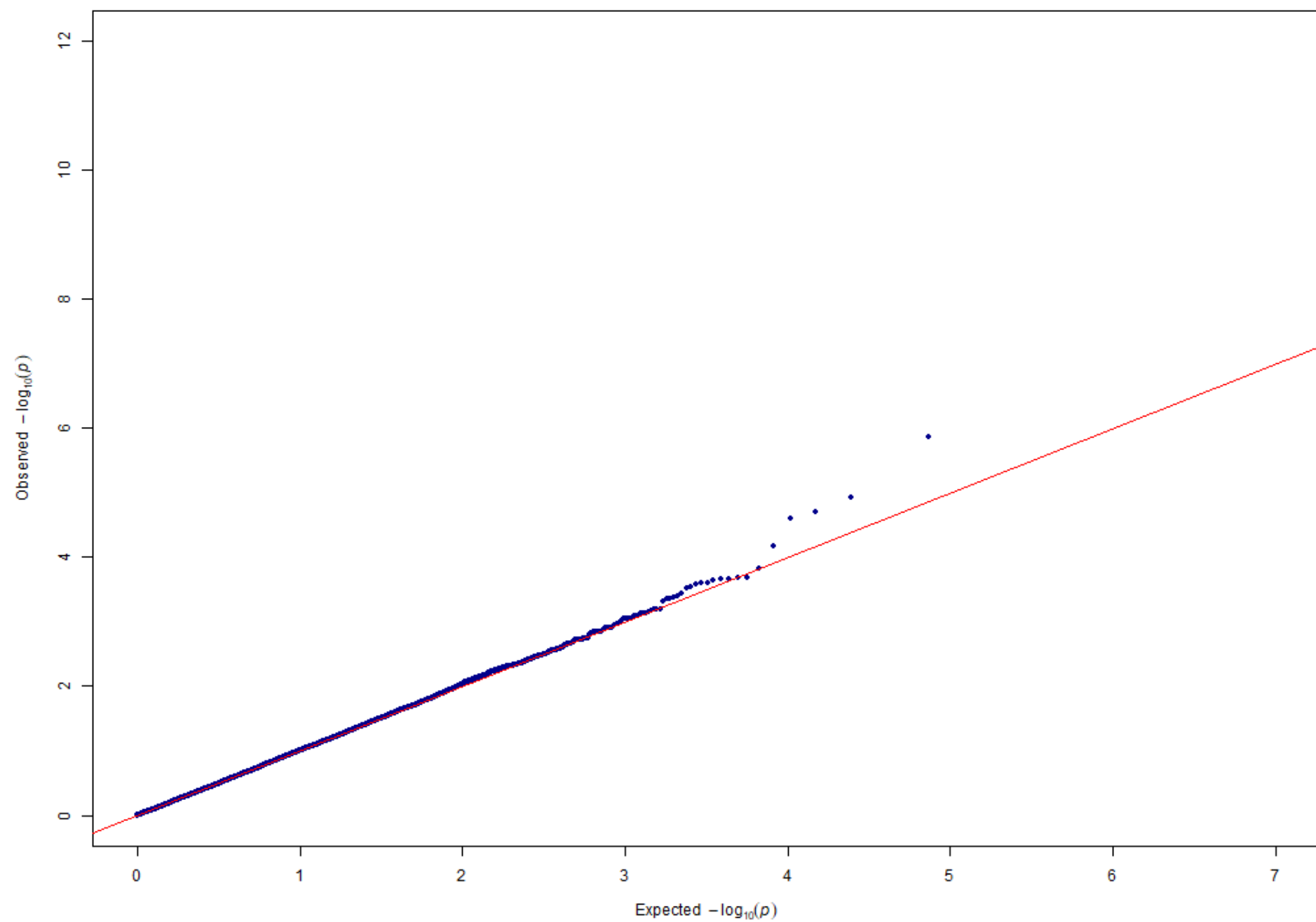

Supplement: Supplementary Materials — Supplementary Material 1: the detailed protocol of PPMI cohort. Supplementary Material 2: Q-Q plot and Manhattan plot of right putamen, right caudate, right anterior putamen, left putamen, left caudate, and left anterior putamen DaTscan EWAS. Supplementary Material 3: Q-Q plot and Manhattan plot of right putamen, right caudate, right anterior putamen, left putamen, left caudate, and left anterior putamen DaTscan GWAS. [file 2893662.f1.zip › Supplementary Material 3/RAP_qq_color.pdf]

### Manhattan plot of right caudate DaTscan GWAS

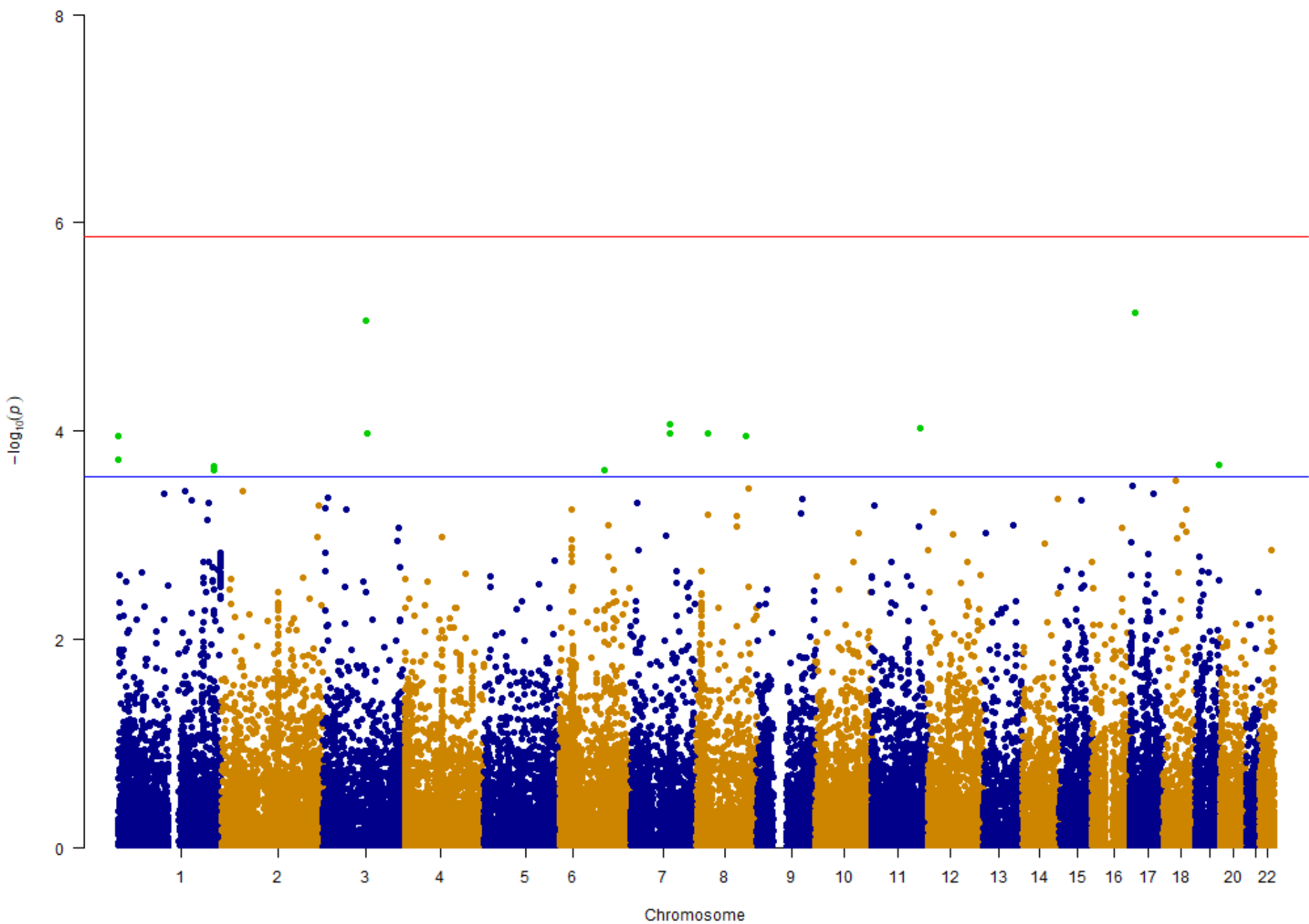

Supplement: Supplementary Materials — Supplementary Material 1: the detailed protocol of PPMI cohort. Supplementary Material 2: Q-Q plot and Manhattan plot of right putamen, right caudate, right anterior putamen, left putamen, left caudate, and left anterior putamen DaTscan EWAS. Supplementary Material 3: Q-Q plot and Manhattan plot of right putamen, right caudate, right anterior putamen, left putamen, left caudate, and left anterior putamen DaTscan GWAS. [file 2893662.f1.zip › Supplementary Material 3/RC_manhattan_color.pdf]

Q-Q plot of right caudate DaTscan GWAS

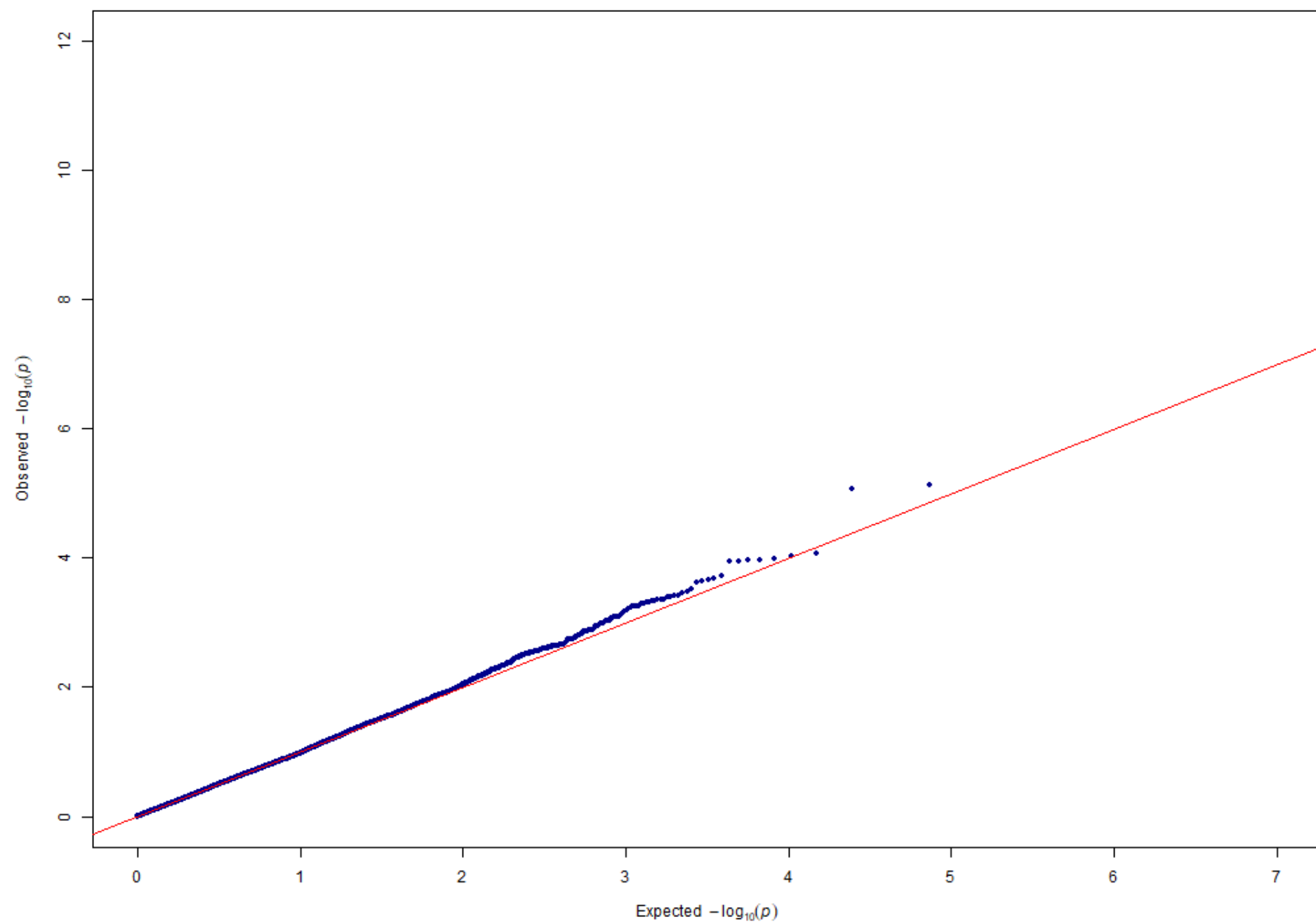

Supplement: Supplementary Materials — Supplementary Material 1: the detailed protocol of PPMI cohort. Supplementary Material 2: Q-Q plot and Manhattan plot of right putamen, right caudate, right anterior putamen, left putamen, left caudate, and left anterior putamen DaTscan EWAS. Supplementary Material 3: Q-Q plot and Manhattan plot of right putamen, right caudate, right anterior putamen, left putamen, left caudate, and left anterior putamen DaTscan GWAS. [file 2893662.f1.zip › Supplementary Material 3/RC_qq_color.pdf]

Manhattan plot of right putamen DaTscan GWAS

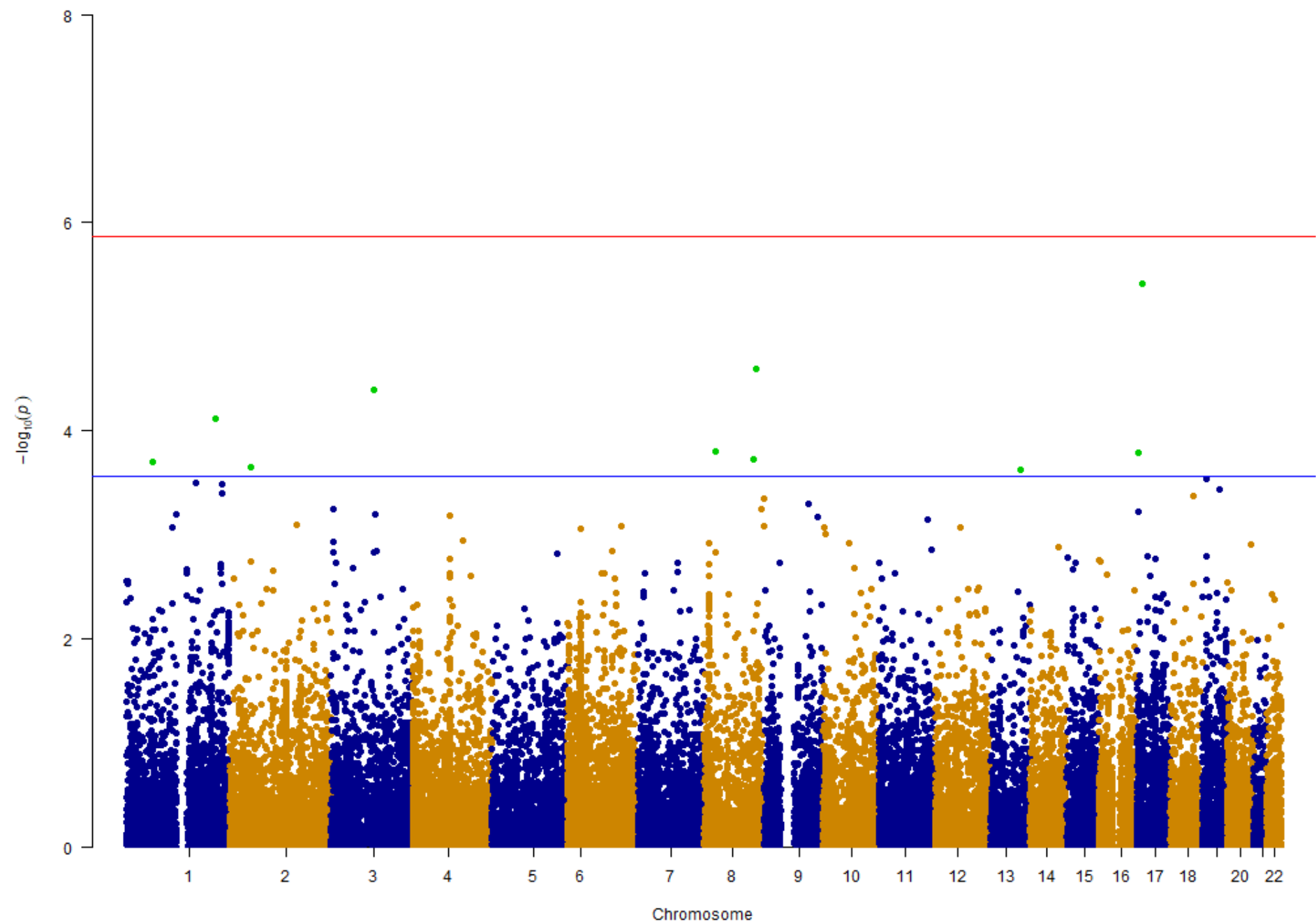

Supplement: Supplementary Materials — Supplementary Material 1: the detailed protocol of PPMI cohort. Supplementary Material 2: Q-Q plot and Manhattan plot of right putamen, right caudate, right anterior putamen, left putamen, left caudate, and left anterior putamen DaTscan EWAS. Supplementary Material 3: Q-Q plot and Manhattan plot of right putamen, right caudate, right anterior putamen, left putamen, left caudate, and left anterior putamen DaTscan GWAS. [file 2893662.f1.zip › Supplementary Material 3/RP_manhattan_color.pdf]

Q-Q plot of right putamen DaTscan GWAS

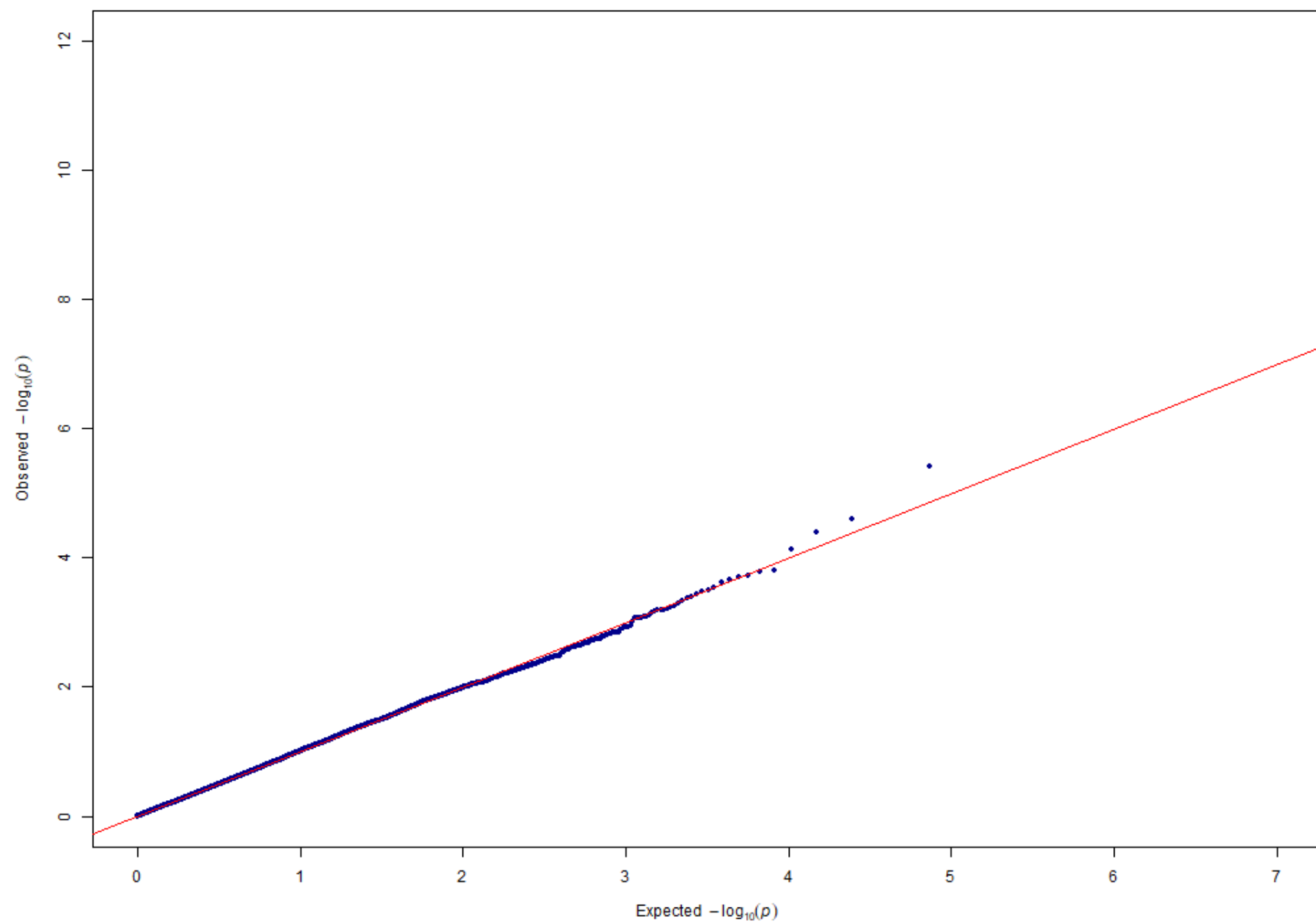

Supplement: Supplementary Materials — Supplementary Material 1: the detailed protocol of PPMI cohort. Supplementary Material 2: Q-Q plot and Manhattan plot of right putamen, right caudate, right anterior putamen, left putamen, left caudate, and left anterior putamen DaTscan EWAS. Supplementary Material 3: Q-Q plot and Manhattan plot of right putamen, right caudate, right anterior putamen, left putamen, left caudate, and left anterior putamen DaTscan GWAS. [file 2893662.f1.zip › Supplementary Material 3/RP_qq_color.pdf]
